# Long-Range Autocorrelations of CpG Islands in the Human Genome

**DOI:** 10.1371/journal.pone.0029889

**Published:** 2012-01-11

**Authors:** Benjamin Koester, Thomas J. Rea, Alan R. Templeton, Alexander S. Szalay, Charles F. Sing

**Affiliations:** 1 Department of Human Genetics, University of Michigan, Ann Arbor, Michigan, United States of America; 2 Department of Physics and Astronomy, Center for Astrophysical Sciences, Johns Hopkins University, Baltimore, Maryland, United States of America; 3 Department of Biology, Washington University, St Louis, Missouri, United States of America; Bellvitge Biomedical Research Institute (IDIBELL), Spain

## Abstract

In this paper, we use a statistical estimator developed in astrophysics to study the distribution and organization of features of the human genome. Using the human reference sequence we quantify the global distribution of CpG islands (CGI) in each chromosome and demonstrate that the organization of the CGI across a chromosome is non-random, exhibits surprisingly long range correlations (10 Mb) and varies significantly among chromosomes. These correlations of CGI summarize functional properties of the genome that are not captured when considering variation in any particular separate (and local) feature. The demonstration of the proposed methods to quantify the organization of CGI in the human genome forms the basis of future studies. The most illuminating of these will assess the potential impact on phenotypic variation of inter-individual variation in the organization of the functional features of the genome within and among chromosomes, and among individuals for particular chromosomes.

## Introduction

Our understanding of the structure, organization and function of the human genome has increased exponentially over the past 60 years. Molecular studies of the central, gene to protein, dogma have revealed an unimagined richness in structural DNA variation [1] that highlights the difficulty in defining a gene effect [2] and the complexity of the involvement of DNA sequence variation in determining phenotypic variation [3]–[5]. We have also known for over 70 years that a gene's function can be changed by altering its physical location within the genome [6]. A chromosomal neighborhood influences function through structural relationships between numerous components [7], [8]. They include the protein coding sequences, regulatory sequences such as non-coding RNAs, and epigenetic markings whose interactions with chromatin influence the higher order folding of the genome [9].

An appreciation of the functional impact of variation in the organization of these components on variation in gene activity [10] and human health [11]–[12] has emerged in the last decade. Consequently, it has become widely appreciated that the separate components do not influence phenotypes independently of variations in the micro-cellular or macro-organismal environments. Nowhere is this more evident than in the relationship between environment, epigenetic patterns, and phenotype [13], and this complexity has likewise been recognized in the interplay between evolution, development, and the organization of the genome [14]. As Noble [3] and Lewontin [15] have so clearly summarized, the DNA sequence is only part of the material basis of heredity: the biological functions of a sequence that behaves as a gene are determined by the interactions of its effect with the effects of other genes and environmental agents. These agents can be internal or external to the organism, and their coordinated effects occur throughout the life cycle from fertilization until death. This is elegantly displayed by the relationship between body-mass index, variably methylated regions, and environment [16]. Interaction among these agents can alter characteristics of the organization of the genome, such as patterns of methylation, that are manifest as effects on traits in the hierarchy that connects the genome to clinically relevant endpoints.

The communication of the environment with the genome takes several forms. Histone modifications and DNA methylation are two common epigenetic (“above the genome”) mechanisms that influence the impact of the information coded in the DNA sequence on the development and expression of a phenotype [17]. It is well-known that these mechanisms also act more globally in tissue differentiation [18] or in various human diseases [19]. Particularly in the latter case, the distribution of these processes across the genome determines which genes are influenced and variation in this distribution among individuals may be associated with inter-individual phenotypic variation [16], [20].

DNA methylation in mammals is thought to occur predominantly, but not exclusively, at CpG dimmers [10], [21]. CpG islands (CGI), which are especially rich in CpG dimers, have drawn attention as sites of differential methylation. They have demanded special attention because approximately 40% of CGI are found in promoter regions [22], [23]. While they are predominantly unmethylated, in certain instances CGI can become methylated. For instance, genome-wide differences in the methylation state of the CGI in tumor tissue are known to occur in comparison to normal tissue [24]. It has also emerged that unmethylated CGI influence histones and thereby modify the local chromatin state [25]–[27]. Beyond the epigenetic considerations, CGI have also been associated with numerous other functionally-relevant genomic features including: recombination hotspots and the presence of transposable elements [28]–[33], domain organization and nuclear lamina interactions [34], origins of replication [35], [36], and local mutational processes [37].

Traditional statistical approaches have been regularly employed in studies of CGI to estimate the total number, the number and density per chromosome or genome, and the number and density as a function of the region on a chromosome [22]. Among other things, they have been shown to exhibit a non-uniform distribution across different regions of the genome [38] and to vary in frequency across many species [39]. Intriguingly, Illingworth et al [40] recently investigated CGI frequency and position in humans and mice in more detail and found that the abundance and positions of CGI relative to genes are in fact conserved between humans and mice. The frequency-based statistics inherent in these methods are all first moments of the CpG island distribution and their information content about organization across the genome is limited. FISH analysis of karyotypes [38] provides a low resolution look at clustering, while density profiles likewise offer only a qualitative view of moments beyond the mean. Perhaps more importantly, the treatment of biases in the measurement of the DNA sequence is unclear. For instance, the impact of large portions of missing sequence on any of these statistics is not rigorously addressed. It follows that a challenge for the study of genome organization is to develop metrics that quantify the higher order moments of the CGI distribution while simultaneously minimizing the effects of such biases on the statistical characterization of the distribution of CGI across a chromosome. The combination of the functional relevance of CGI, the relative ease of detection, and the abundance of full genome sequences on the horizon make CGI an ideal test bed for an approach that aims to quantify large scale distributions of genomic features.

In this paper we initiate studies to quantify the organization of the human genome which will form the basis of future studies of the impact of inter-individual variation in the organization of the functional features of the genome on inter-individual phenotypic variation. To this end, we adapt and apply the two-point correlation function (TPCF) used widely in astrophysics to characterize the organization of the Universe to the organization of CGI within and across chromosomes of the human genome using the publicly available reference DNA sequence. We quantitatively establish that the distribution of CpG islands is non-random across each chromosome and that the organization of the CpG islands across a chromosome varies significantly among chromosomes. In doing so, we outline a quantitative framework that includes an account of uncertainties and thereby facilitates statistical comparisons of variability in organizational characteristics of genomic features among chromosomes, individuals, or species.

## Methods

### Background

The analytical challenge that we face is not unique to studies of the organization of the human genome. Many complex natural phenomena exhibit long-range correlations, from molecular and biological systems on one end to the distribution of galaxies in the Universe [41] on the other end of physical scales. While gravity is a long-range force, even systems with very short range interactions can develop long-range correlations near the so-called critical point, like the famous Ising model of ferromagnetic systems. For such stationary point processes spatial autocorrelation functions are the method of choice to quantify this behavior. The simplest of these is the two-point correlation function (hereafter TPCF), that corresponds to the “excess” probability over random of finding single objects in two infinitesimal volumes of elements. There are several different estimators for such statistics, namely the cumulative Ripley K- and L-functions [42] and other differential estimators [43]. These are all quite simple, when the stationary random process is described by a constant intensity and has a simple, continuous support (in our case the linear sequence of the genome).

In practice, this is never the case, the support has many “gaps” and internal edges, i.e. holes in the data (missing sequences) and ends of the chromosome. It is clear that points far from the edges have a different probability of having neighbors than the ones close to the edges, where neighbors can only be on one side. Constructing unbiased estimators requires an appropriate “edge correction,” which has been the practical challenge in computing the TPCF. Ripley [44] has proposed an edge-corrected variant of the K-function. Ohser [45], Baddeley [46] and Davis and Peebles [47] have proposed alternative approaches. Each of the proposed estimators provide a first-order correction for the bias associated with the edge effect.

Motivated by galaxy clustering, Hamilton [48] and Landy and Szalay [49] published a more systematic approach (hereafter LS), which is applicable to the cumulative estimators as well. Using an appropriately weighted difference of two different first-order estimators, the LS approach selects weights that cancel the first order errors resulting in an estimator that is accurate to second order. The price of the improved accuracy is an increase in the computational cost. The LS approach was later generalized by Stoyan and Stoyan [50]. They showed that among the proposed edge-corrected estimators the LS estimator has the practical advantages of handling missing data and having minimum variance. In this paper we will use the LS estimator of the TCPF because of its computational simplicity and smaller variance.

### Definition of the TPCF Estimator

We present a brief overview of the LS estimator and refer the reader to details of its derivation in Landy and Szalay [49]. In the case of one-dimensional geometry, as is the case of the genome, the TPCF is formally defined as
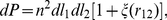
(1)where *dl_1_* and *dl_2_* are line elements at a separation *r_12_*, *n* is the expected number of objects per unit length in the one-dimensional space, *L*, and *dP* is the dimensionless probability of finding two objects in this configuration. In the absence of true correlations ξ is zero. In our computation of the TPCF used here, we will aggregate the pairs at a given separation into a set of logarithmic bins, labeled with the center distance *r*. Letting *x_i_* = 1 designate a data point and *x_j_* = 1, and a second data point with a distance from *x_i_* within the radial bin *r*, we can define the quantity *DD(r)* as
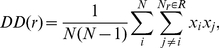
(2)where *N* is the total number of data points, and *N_r_* is the number of data points *x_j_* within the bin *r* from the data point *x_i_*. DD is normalized such, that the integral over all bins adds up to 1. In a similar fashion, we can also compute the quantities *DR* and *RR* as
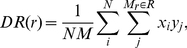
(3)

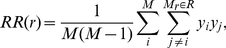
(4)similar to *DD*, except that *y_i_* designates a randomly placed point at location *I*, and the number of random points is denoted by *M*. Thus *DR* represents a cross-correlation between the data points and randomly placed points, while *RR* is the auto-correlation of the random points. The random points are drawn to have the same gaps and edge effects as the ones present in the real data. These quantities are a kernel estimator of the auto- and cross pair counts, using a rectangular kernel (pairs are either in or out of a given bin). The LS estimator can be written as
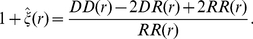
(5)More specifically, the DR term means that we go to each data point in the region *r* and count the number of randomly distributed CGI around each data point in that region. The fact that the random points populate only the regions in which we could have found data ensures that edges and holes are properly taken into consideration in the estimation of *ξ*. The number of random points is chosen to be large enough (we chose 10 times the number of observed data points i.e. *M* = 10 *N*) so that the contribution of the number of random points chosen to the final standard error is negligible.

It is important to note that this estimator of *ξ(r)* treats CGI as point-like, which is not strictly true. The distance measurement could be between CGI centers or between 5′ or 3′ termini. Our exploratory studies of these alternatives using a longer chromosome (4) and a shorter chromosome (19) revealed that the reference point has a negligible effect on scale >1000 bp, so the convention adopted in our analyses of the distribution of CGI was to measure from the 5′ end. In general, difficulties are minimized when the separations considered are larger than the length of the feature under consideration. A comprehensive analysis of this issue involving a range of genomic features in all chromosomes is currently in progress.

A second concern is that the random point distribution must theoretically obey the distribution of the CGI. In addition to avoiding missing sequence, random CGI must not overlap with one another. In placing random CGI in our studies we selected them to have lengths that are randomly drawn from the observed data, and that they do not overlap with one another. We anticipate the application of the strategy proposed here to also have utility in studying the organization and distribution of the many other features beyond CGI structure that have been defined and are currently being investigated by molecular studies of the genome [51].

For this first study, we chose the full chromosome to be the unit of genetic inference. To compute the TPCF and its standard error for each chromosome, resampling methods such as the jackknife and bootstrap are preferable. We chose bootstrap estimates based on 250 resamples with replacement, where each resample consists of the same number CGI as measured on the chromosome. For each resample we compute 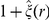 as we would in the real data. An estimate of mean and standard error,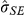, of 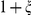 was computed from the resulting distribution of estimates of 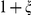 among the 250 resamples. There is no clear prescription for the number of bootstrap resamples required. We determined that 250 was a sufficient number by computing the mean and standard error of bootstrap samples of various sizes until the mean and standard error of the mean asymptote. Moreover, it is encouraging that the “bootstrap bias” (not shown), which quantifies the difference between the bootstrap estimate of the mean and the native estimator in equation (4), is of the order of 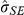. In the results of the analyses of the human reference sequence presented below we plot the mean of the bootstrap estimates and the error bars are given as 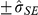.

Finally, the detailed statistical properties of the estimator of the TPCF we employ here and other estimators have been explored exhaustively in the astrophysical literature [49], [52] for their behavior on different distance scales and in different density environments. The estimator we use is among the most stable in applications in astrophysics. We relegate a full exploration of the effects of scale and density on the estimators of the TPCF in genomic data to a future work.

### A Chromosomal Metric for the TPCF

To create a simple metric of the TPCF for a chromosome as the unit of genetic inference we recognize three distinct regions of the TPCF. First, separations of order <1000 bp should be viewed with caution, as they are similar in size to CGI. Algorithmic effects are more likely to leave an imprint on the spatial distribution of CGI at these scales. Moreover, we arbitrarily defined the location of the CGI as the first 5′ base, but we could have just as well picked an alternative position. This choice also has a slight effect on close (<1000 bp) pairs. Second, at scales of a few Mb or more within a chromosome the TPCF yields values consistent with random, indicating that CGI have little or no structure on large scales. Third, on intermediate scales, each chromosome exhibits an approximately linear relation on a log-log plot. It is these intermediate scales that are essentially free of small scale CGI algorithmic effects, and informative with respect to non-randomness. With these considerations in mind, we fit power laws of the form
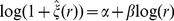
(6)to the intermediate range of data using a χ^2^-minimization procedure [53]. This prescription minimizes the weighted sums of squares, where the weights are given by the inverse of the measurement errors on each point. When the errors are Gaussian the χ^2^-minimization procedure yields the maximum likelihood solution [54]. As this fit is performed on a log-transformation of the data, we are careful to transform the measurement errors used in the weighting. Errors on the parameter estimates are given as well, and are taken from the diagonal elements of the covariance matrix given by the maximum likelihood solution.

We employed a Monte Carlo approach to estimate the confidence interval for the regression line computed for each chromosome.For each chromosome, we create a realization, *i*, of the 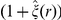 at each separation by randomly drawing from a Gaussian distribution centered on 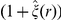, with a second moment given by the estimator of the variance of 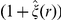, 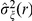 given by equation (7) below. This realization is then fit using equation (6). This procedure is then replicated 10,000 times. We then sort the resulting 10,000 Monte Carlo values of 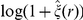 from lowest to highest and denote the upper value of the confidence interval at an arbitrary separation, *r*, as the value of realization 9,950 and the lower value of the confidence interval as the value of realization 50.

Landy and Szalay [49] derive estimators of σ_ξ_^2^ that are complex in their implementation, and specific to the astrophysical context in which they are used. To derive an approximate estimate of the variance, we proceed under the assumption that the underlying random processes that drive the distribution of CGI are Poisson in nature. Using basic error propagation techniques [53], the resulting expression for the variance reduces to a simple form in the limit when *M≫N*, i.e.
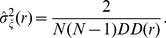
(7)This expression for the variance in the Poisson situation has been discussed elsewhere [49]. Other sources of error that are not explicitly Poisson will influence the true variance, so this procedure results in an approximation. The most obvious source of non-Poisson uncertainty is the mere existence of significant clustering in the data, which is not present in a Poisson process: the stronger the clustering, the poorer the approximation. The detection algorithm or the biochemical assay used can also impart more subtle sources of non-Poisson. A full treatment of this topic will be considered in a future work.

## Results

### Source of DNA sequence data

We illustrate the application of the TPCF to data available from the latest haploid assembly build of the human genome from the Human Genome Reference Consortium, “GRCh37.” Investigation of the organization of CGI starts with algorithmic identification of the sequences that define them. We acknowledge that the identification of CGI depends on the properties of the algorithm employed. CGI were first defined systematically by Gardiner-Garden and Frommer [55] through their elevated GC content and association with 5′ ends of vertebrate genes. Han et al. [56] review a range of commonly used algorithms that have since improved on this basic theme. More recently Irizarry et al. [57] have presented a more generalized CGI detection algorithm built upon a hidden Markov model. For our study we have used the algorithm suggested by Takai and Jones [22] optimized to detect CGI in promoter regions while minimizing contamination from *Alu* repeats. We revisit the importance of the definition of CGI in the discussion.

### Descriptive Statistics

**Figure 1:**
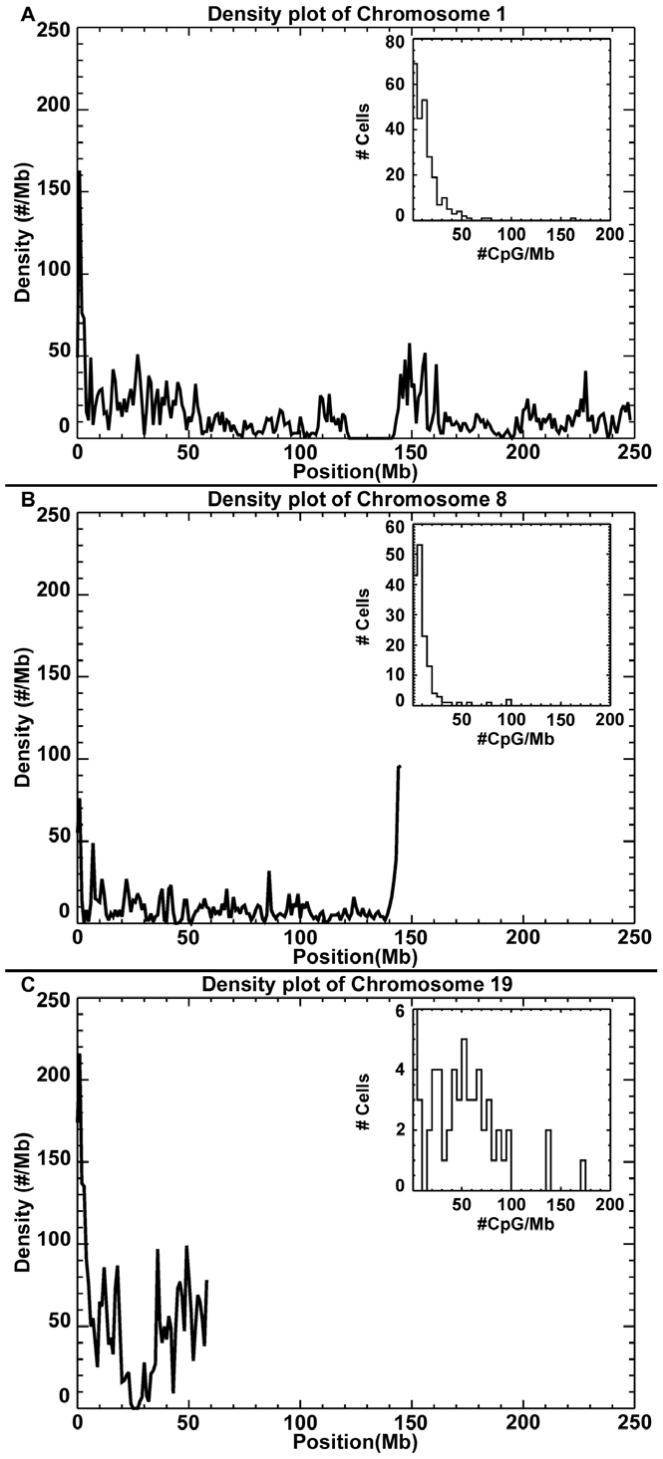
Density plots for chromosomes 1, 8 and 19. Densities are defined as the number of CGI per 1 Mb window. Note the especially high density at the 5′ telomere and the missing sequence at position ∼130 Mb. Inset shows the distribution of densities in the 249 1 Mb windows that comprise the chromosome. The distribution is skewed and demonstrates that simple estimators of the centroid and dispersion are insufficient. Plots for all chromosomes are given in Text S1.

The densities of CGI in one Mb windows across each of the 22 autosomes and the X and Y sex chromosomes are given in Text S1 (see online access). For the purpose of illustrating the variability of the intra-chromosomal local variation in the CpG densities among chromosomes, data on chromosomes 1, 8 and the shorter more gene dense chromosome 19 are presented in Figure 1a–c. In general, the local variation within a chromosome is not uniformly distributed. Typically, the largest coherent fluctuations appear on scales of ∼10 Mb, while smaller scales are lost in the resolution of the window. Gaps in the sequence appear (for instance in the middle of chromosomes 1 and 19, Figure 1a and 1c) as do regions of highly enhanced density, especially near the telomeres for all chromosomes. The higher density of CGI across chromosome 19 corresponds to the increased density of protein coding genes in this chromosome.

In general, the frequency distribution of the density of CGI per Mb is positively skewed (see inserts in Figures 1a–c for chromosomes 1, 8 and 19 and for all chromosomes in Text S1). The shorter the chromosome the more uniform the frequency distribution of the density of CGI is per Mb (e.g. chromosome 19, Figure 1c). Statistics that summarize the frequency distribution of CGI densities per MB for each chromosome are presented in Table 1. Column 2 gives the approximate number of one Mb windows for each chromosome. Column 3 gives the number of Mb that have no data and column 4 lists the total number of CGI that has been detected for each chromosome. We present both the average density of CGI per Mb of assayed data (column 5) and the average density of CGI per MB for the total length of the chromosome including the regions that were not assayed (column 6). The similarity of these values across chromosomes suggests that the missing regions of the genome that do not have CpG island information available in the reference sequence are randomly distributed across local regions of each chromosome.

**Table 1:** Summary statistics for the CGI densities for each chromosome.

Chromosome	Total Length (Mb)	Missing (Mb)	N_CGI_	Average Density per Mb assayed*	Average density per Mb of Chr	Standard deviation of the distribution of density per Mb of Chr	Skewness of the distribution of density per Mb of Chr
1	249.3	24.0	3430	15.2	15.1	16.0	4.3
2	243.2	5.0	2553	10.7	10.8	9.5	1.8
3	198.0	3.2	1814	9.3	9.3	8.1	2.0
4	191.2	3.5	1664	8.9	9.0	11.8	4.9
5	180.9	3.2	1884	10.6	10.6	13.8	4.0
6	171.1	3.7	1954	11.7	12.0	12.0	2.1
7	159.1	3.8	2256	14.5	14.7	19.2	3.3
8	146.4	3.5	1562	10.9	10.9	14.2	4.0
9	141.2	21.1	1814	15.1	14.7	15.3	3.2
10	135.5	4.2	1733	13.2	12.6	13.5	4.4
11	135.0	3.9	1776	13.5	13.9	14.8	2.8
12	133.9	3.4	1832	14.0	13.7	13.6	2.1
13	115.2	19.6	959	10.0	10.3	14.4	4.2
14	107.3	19.1	1180	13.4	13.2	13.0	2.3
15	102.5	20.8	1187	14.5	14.2	9.3	0.9
16	90.4	11.5	1894	24.0	23.5	28.5	2.3
17	81.2	3.4	2210	28.4	28.0	22.3	1.4
18	78.1	3.4	805	10.8	11.2	15.0	4.8
19	59.1	3.3	3147	56.4	55.9	40.6	1.6
20	63.0	3.5	1111	18.7	18.5	20.5	3.2
21	48.1	13.0	502	14.3	13.1	16.2	2.0
22	51.3	16.4	976	28.0	26.6	17.8	1.2
X	155.3	4.2	1541	10.2	10.5	12.6	3.7
Y	59.4	33.7	311	12.1	11.3	20.6	3.4

Column 1: Chromosome.

Column 2: Length in Mb (including missing sequence that was not assayed).

Column 3: Ambiguous or missing sequence in Mb not assayed.

Column 4: Number of CGI detected.

Column 5*: Density = N_CpG_/(Total Mb – missing Mb not assayed).

Column 6:* Mean number CGI per Mb for entire chromosome.

Column 7: Standard deviation of number of CGI per Mb for entire chromosome.

Column 8: Skewness of number of CGI per Mb.for entire chromosome.

*The density is simply computed as the total number of CGI/chromosome length in Mb that have been assayed. This is formally *not* the same as the mean number of CGI per Mb of chromosome ignoring the missing Mb, which we compute by counting CGI in windows of 1 Mb and computing the mean, standard deviation and skewness of the resulting distribution.

When missing regions are included in the calculations, there are several fold differences among chromosomes in the average density of CGI over all windows of one Mb in size (column 6), the standard deviation (column 7) of the density among those windows and the positive skewness (column 8) of the frequency distribution of the densities. The average density of CGI per Mb ranges from 9.0 to 55.9 and the standard deviation among windows ranges from 8.1 to 40.6. The frequency distribution of CGI per Mb for every chromosome is significantly skewed to the higher values (p<0.001). As expected, larger average numbers of CGI per Mb window are associated with greater variability among windows. There is a statistically significant negative rank correlation between chromosome length and average density (Rho = −0.50, p = 0.01) and between length and standard deviation of the density (Rho = −0.62, p = 0.001). There is no evidence for a significant correlation between the skewness of the distribution of CGI per Mb and chromosome length (Rho = 0.35, p = 0.09).

### Two-Point Statistics

The two point correlation function described above provides a means to quantify the spatial distribution of CGI. We first generated the random points for the regions where sequence is missing (Column 3, Table 1). For each chromosome we generated 10 simulated chromosomes of the same length containing the same number of CGI with spatial distributions that avoid the masked (ambiguous) sequence to represent the space in which we could have discovered CGI. The factor of 10 oversampling of random points ensures that our results have a negligible contribution from statistical error associated with the number of random points used.

**Figure 2:**
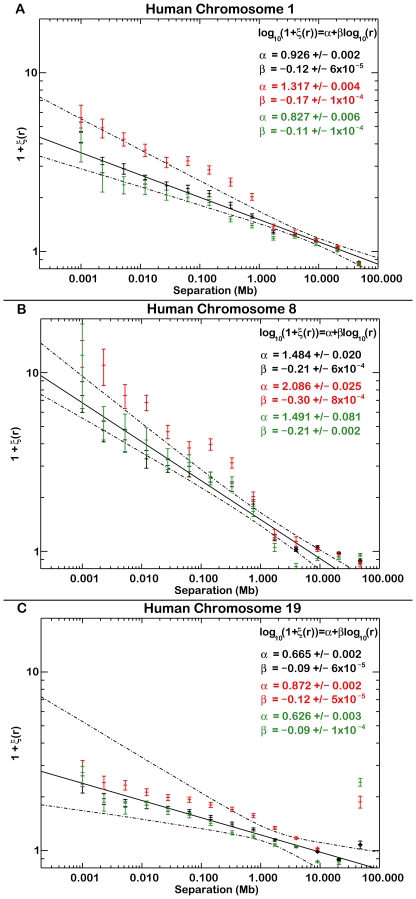
The Two Point Correlation Functions of CGI in Chromosomes 1, 8 and 19. The vertical axis shows value of the two-point correlation function, estimated using the bootstrap mean 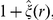 (see methods), and error bars are 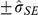. The expectation in the absence of clustering is 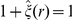. CGI using the Takai and Jones (2002) algorithm are shown in black, as are the best-fit power law models. Dotted lines show an approximate 3σ confidence intervals derived from a Monte Carlo based on the bootstrap estimate of ξ and our estimate of its variance (see Methods). Also shown in red (green) are the TPCF for the CGI given by Irizarry et al [56] (Illingworth et al [40]) and the associated regression coefficients also in red (green). Remaining chromosomes can be found in Text S2.

We binned the pairs of CGI by separation in 15 logarithmically spaced bins between 1000 bp (the median CGI size) and 249 Mb (the length of chromosome 1). The same bins are used for all chromosomes. The results of our analyses of the autosomes and the X and Y chromosomes are presented in the Text S2 (see online access). Representative examples are given in Figure 2a–c. On log-log axes, we plot 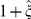 on the vertical axis against the separation *r*. The value of 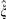 reflects the excess correlation above random. For instance, 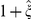 = 2.5 at a separation of 0.01 Mb means that one is 2.5 times more likely than random expectation to find a CpG island. For each chromosome there is significant evidence for “clustering.” The estimate of 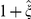 is much greater than one out to nearly 10 Mb. The clustering is strongest at small separations and weakest at large separations, where it decreases to the random expectation. The density plots in Figure 1 show typical fluctuation at scales of a few Mb, which suggests CGI tend to cluster together, but the detailed structure is washed out in the windowing process. Indeed, it can be shown that these density plots are just integrals over the two point correlation functions.

In addition to quantifying the CGI distribution for its own sake, we ultimately seek to describe and to compare chromosomes or sub-chromosomal regions between and among individuals. For each TPCF, which are linear to first approximation on the log-log plots, we fit power-laws (see Methods). For each chromosome, we identify the first point that is consistent with random and then only consider points from shorter separations in the fit. The legend in each plot shows the results of this fit. Confidence intervals (see Methods) on each regression line are indicated by dashed lines.

While hints of a non-linear relationship between 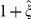 and separation exist for various chromosomes, the data typically do not deviate from the model by more than a standard error. The simplicity of the power law motivates this choice of model, but where large deviations from the power-law are found, models with more degrees of freedom and with a stronger biological motivation may reveal novel insights about both CGI detection methods and the processes driving the placement of CGI in the genome. For the power-law fits, in all cases, the traditional χ^2^-goodness of fit
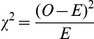
yields a χ^2^ with p>0.99. The error-weighted goodness-of-fit (e.g. Press et al. 1992) [53]

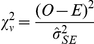
also suggests that the power law-model is a good fit. χ^2^_ν_∼1 for all but the Y chromosome.

**Figure 3:**
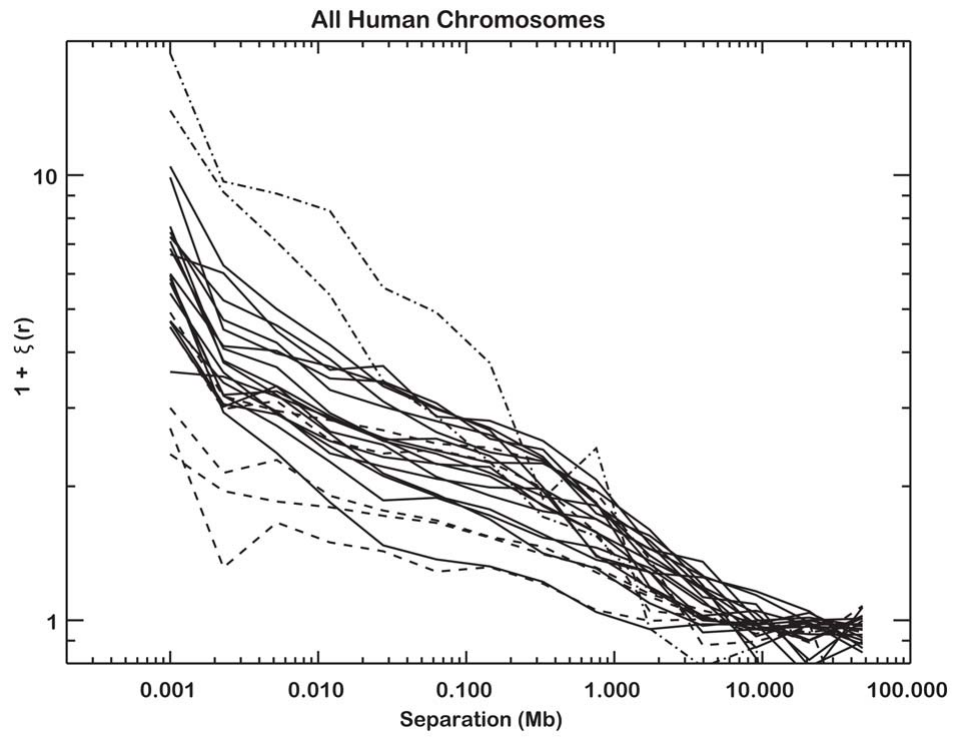
Summary of TPCF for all human chromosomes. Dashed lines show high CGI density chromosomes, dash-dot lines represent (top to bottom) the Y and X chromosomes. Inter-chromosomal variation is clear, and in general all chromosomes show random clustering by ∼10 Mb. Large separations likewise produce significant clustering, due to the high density of CGI in telomeres. Typical error bars for a given 1+ξ are shown to the right. Individual profiles for each chromosome can be found in Figure 2 and the Supplemental Figures.

Finally, Figure 3 shows the two point functions of all autosomes over-plotted, with typical error bars at the right. The variation in clustering of CGI between chromosomes is marked. For reference, chromosome 4 shows the most extreme clustering (a factor of 10, uppermost line) while 19 shows the weakest clustering (lowermost line). Interestingly, a few chromosomes exhibit significant clustering on the largest scales. These scales are of the order of each chromosomes length and reflect the excess clustering of CGI near telomeres that is revealed by the density plots. The detailed profiles for each chromosome can be found in Text S2 (see online).

## Discussion

### Relevance of the proposed analytical strategy

A search for the understanding of the role of each of the multiple features of the human genome has dominated the research agenda of molecular biology ever since the discovery of DNA. However, measurements of global genome features such as chromatin modulation, chromosome inactivation, stability, repair, imprinting, transposition, repetitive DNA dynamics, transcriptional activation and repression, as experimentally daunting and productive as they have been, have suffered from the absence of a global genome metric that summarizes the vast informational content they represent within and among chromosomes. Our study is the first to rigorously measure human whole genome sequence organization of biologically relevant motifs by establishing a genome wide metric based upon a two point correlation function originally optimized for astrophysical research on the organization of the Universe.

Earlier studies to detect correlated structures in genomic data were carried out by the signal processing and information theory communities in the late 1950's [58], [59]. At that time researchers had access to only small amounts of data on chromosome composition and effectively no sequence data. Some thirty years later the availability of partial DNA sequence data and the initiation of online shared databases fostered research [60]–[63] that suggested that a power-law, and possibly fractal patterns, were characteristics of the DNA sequence (see Knoch et al. 2009 [64] for a review). Li [65] addressed the applicability of such mathematical models as a means to study the organization of genomes, but cautioned against making generalizations because available DNA sequence information was sparse and very local. Recent work by Chapeau-Blondeau [66] and Knoch et al. [64] adds further support for a power law structure in the distribution of DNA bases.

These efforts to measure and model the organization of nucleotides on large scales parallel our work, but there are important differences. First, our analytical method involves a statistical strategy for dealing with missing sequence. It is obvious that even in the large whole-genome scale sequencing era, a full sequence is not guaranteed, and that statistical methods must be employed to correct for this experimental reality. Second, and perhaps most important, our strategy for estimating measures of genome organization and their standard errors establishes a quantitative basis for future studies of the impact of variability in patterns of DNA sequence organization. Variability can then be assessed within and among chromosomes, among individuals for regions of chromosomes, whole chromosomes, or even the whole genome. Third, for illustrative purposes, we use CGI, which is a biologically well-motivated choice, both for their proximity to other functional sequences and their central role in facilitating epigenomic modifications. Importantly, while future studies may show that the spatial CGI distribution does vary among individuals, the approach we demonstrate here can be adapted to other genomic features whose distributions may vary among individuals.

Summary statistics of the distribution of CGI provide a snapshot of the whole genome as it is defined by a set of markers whose computational selection has the features of uniformity and reproducibility. As markers of the organization of the genome, CGI have another particularly attractive feature: their presence is not inferred by association. For instance, in studies of higher organisms that use known candidate genes, promoter regions of those genes, only exome sequence variations or randomly placed marker variations, a very biased and poorly understood subset of the genome is employed to infer its large-scale structure. The representativeness of the subset of the genome studied using selected regions is very difficult to quantify. Although CGI algorithms using the complete DNA sequence differ in their detail and are not completely free of bias, the algorithmic nature of the detection mechanism ensures that their ascertainment is well-understood up to errors in the underlying sequence. CGI also enjoy a peculiar feature not typically associated with DNA: they do not have a preferred orientation. That is, in principle, a CGI will be detected regardless of the strand under consideration, and regardless of whether one's reading frame proceeds 5′ to 3′ or vice versa. This fact is especially important to keep in mind when considering clustering scales comparable to the size of the object under scrutiny.

### Biological implications of the distribution of CGI clustering

We have explicitly demonstrated that CGI cluster together in a manner that depends on both their physical separation and the context of the chromosome of the reference human DNA sequence. Given a CGI, one is more likely than random to find additional CGI nearby. The average clustering is non-zero to ∼10 Mb in all chromosomes and varies between chromosomes by 5 fold or more at distances on the order of 0.1 Mb or less. That CGI cluster should not come as a surprise. It is well known that genes cluster and that CGI are largely found in gene-associated promoters. Variation of clustering of CGI among chromosomes is consistent with the work of Knoch et al. [64] that suggests that the variability in the organization of chromosomes among species is tightly controlled by evolutionary forces. Most important, the distributional properties of CGI summarize functional properties of the genome that are not captured when considering separate variable sites in the primary DNA sequence. While the causes of clustering at any scale is yet unknown, clues come from the functionally-relevant sequences that are physically associated with CGI. Evidence that CGI co-aggregate with promoter regions, transcriptional start sites, or recombination hotspots [67], [68], suggests a role in regulation as well as in biological processes that act to replicate and reshuffle elements of the genome.

As a very basic example of a mechanistic interpretation of the TPCF, the non-random CGI-CGI clustering on scales <10 Mb is suggestive of the notion that tissue-specific genes cluster (e.g. Lercher et al. [69]), and perhaps more intriguingly, is consistent with hierarchical packing found in chromatin structure. At large scales, Bornfleth et al. [70] and Cremer et al. [71] note that subchromatin domains in human chromosomes of ∼1 Mb in size show temporal displacement, both through self organizing Brownian motion [51], [72] and perhaps, to some degree, via undefined directed mechanisms. This packing hierarchy includes the 30 nm chromatin fibers (a few kb of DNA) and extends to smaller scales, where nucleosomes consisting of sequences of ∼147 bp [73], [74] are wrapped around histone octamers and regulate local access of transcription machinery to DNA, in the traditional “beads-on-a-string” configuration. If CGI are typically found in promoter regions of coding sequences, this clustering measurement points to a possible global organizational principle of the human genome, namely, that genes are positioned in the genome so as to exploit the chromatin packing machinery that in part governs transcription. Misteli [75] reviews the organization of chromatin that spans these large and small scales. The fact that the clustering strength CGI scales with distance could be a manifestation of this hierarchical packing and may yield further organizational insights. The notion that measurements like ours couple to the 3-D chromatin architecture has also been invoked in the interpretation of long-range correlations in sequence structure reviewed in Knoch et al. 2009 [64].

The emergent relationship between distribution, clustering and function suggests a framework for the interpretation of methylation data. A recent study in Arabidopsis found that nucleosomal regions are sites of differential methylation [12]. If this extends to humans, where there is increasing evidence for differential methylation at CGI [75], we might expect variation in the clustering of methylated CGI (mCGI-mCGI clustering) between tissues or between diseased and health individuals, even though the locations of the CGI themselves are highly conserved. The data now exist to test for non-random long range patterns of methylation in humans [76], and for the existence of variabililty among tissues. Work is underway to adapt our statistical formalism to these data. The growing list of diseases arising from chromatin packing defects [75] further supports this global approach as genome and methylome data become available for more than a handful of individuals.

The applicability of the organizational information also extends to studies of the molecular genetic mechanisms that drive the distribution of CGI. This in part depends on the presumed function of CGI, which in turns depends on the working definition of CGI (e.g. Hackenberg et al. 2010 [77]). Because of the widespread use of the algorithm of Takai and Jones [22], an algorithm designed to detect CGI in promoter regions, we chose to use it as part of the proof-of-concept in this paper. However it has been pointed out that this algorithm is restrictive, and that only ∼35% of the CGI are associated with promoters [23]. While it is not the goal of this paper to reconcile differing conventions for CGI, it is nevertheless instructive to reconsider the inclusion of the Takai and Jones algorithm in presenting our results. With the availability of a full genomic sequence the definition of CGI can be improved to create more complete lists of CGI near transcriptional start sites (TSS). Irizarry et al. [56] and Wu et al. [78] describe a detection algorithm built upon a hidden Markov model that they demonstrate more completely detects CGI near TSSs. This algorithm can be adapted to different species and has the additional attractive feature that it assigns probabilities to putative CGI. In Figure 2, and for each chromosome in Text S2, we plot in red the TPCF for the publicly-available CGI made available by Irizarry et al [57]. The same general trend in deviation from random clustering with separation is present in the power-law indices, but at a generally higher amplitude in most chromosomes with some evidence for deviations from linearity. The higher abundance of CGI detected by the Irizzary algorithm is one possible explanation for the increased amplitude. Cases where the amplitudes differ and β agrees between the two algorithms suggest that the additional objects are themselves distributed similar to detected by the Takai and Jones algorithm. In this sense vastly different β values (e.g. chromosome 13) may be indicative of the different definitions of CGI employed by the two algorithms, and possibly that biologically distinct populations of objects are being mixed.

CGI detected by biochemical means show the same general trends. A set derived from CAP-seq and made public by Illingworth et al [40] are overplotted on Figure 2 in green. Again, the amplitudes are generally lower then the Irizarry et al. [57], and the slope of the power laws are in general agreement with the computationally detected CGI.

The features present in the TPCF for any of the definitions of CGI may arise from one of several underlying factors associated with the nature of the CGI distribution. The global population of CGI may be comprised of several inherently different sub-populations of CGI with different functional properties whose clustering contributes to the observed TPCF at different scales. The example application of the TPCF presented here takes the whole chromosome to be the unit of inference. This definition ignores the possibility of variation in the strength of clustering at smaller scales within and among chromosome arms. Variation in the TPCF with separation among chromosomes (Figure 3) may thus reflect underlying organizational variability among regions of a chromosome, or more problematically, large scale sequencing errors that are not random with respect to position on the chromosome. Finally, the reference sequence analyzed in this work is known to be an aggregation of sequence from many individuals. Should the distribution of CGI and clustering among individuals vary significantly among chromosomes, the observed organizational variability among chromosomes of the reference sequence would be a combination of contributions from both chromosomal differences and differences among individuals. While the true scope of inter-individual variation in measurements like these remains unknown, and is likely minimal for highly-conserved sequences like CGI, the observed range of variation among chromosomes suggests to us that measurements like this may have utility in measuring variation in the distribution of features of the genome among individuals.

Studies of inter-chromosomal variation in genome organization offers a new perspective for measuring the mechanisms of chromosomal evolution. Zhang [79] highlights the importance of gene duplication (e.g. Bridges 1936 [6]) as a means for generating raw genetic material via unequal crossing over or retroposition, among other mechanisms. As CpG island densities track gene densities in the human genome (e.g. Lander et al. 2001 [29]), it may be that the decreased organizational structure (i.e. the decreased two point correlation function amplitude) in higher density chromosomes reflects a suppression of gene duplication mechanisms that have a higher chance of interfering with neighboring sequence, and thus a greater likelihood creating a deleterious phenotypic effect.

### Biological relevance of proposed strategy beyond CGI

The methods presented are easily extended to include any sequence motif. With the promise of routine full genome sequencing just around the corner, computationally identified markers like CGI are well-suited to exploit the wealth of data derived from next-generation sequencing technologies. The next generation of genotype-phenotype studies will require new metrics of organization to evaluate the contribution of variation in organization of the genome to variation in phenotypic effects. The proposed TCPF strategy for measuring organization is only a first step in developing the statistical strategies for evaluating the impact of genome variation on phenotype variation as a conceptual alternative to the SNP based association study paradigm that currently pervades genetic studies. While we use CGI as a demonstration, this strategy may also be applied to studying inter-individual variability in the collective number and spatial organization of other genomic features which play a coordinated role in determining genome function, including methylation patterns and the distributions and clustering of copy number variants, transposons, pseudogenes, mutational hotspots, recombinational hotspots, segmental duplications, short tandem repeats, indels and functional non-translated RNAs.

In conclusion, we recall that the statistical methods presented here were first developed for characterizing and extracting information from the three dimensional relationships among bodies in the physical Universe. In years to come, the next logical step beyond using them for the study of organization of the features of the genome will be to consider their application and utility in measuring three dimensional relationships in cellular space and investigating the role of variation in those relationships in understanding and predicting phenotypic variation.

## Supporting Information

Text S1 **Density plots of CGI for All Human Chromosomes.** As in Figure 1, density is simply defined as the number of Takai and Jones CGI per non-overlapping 1 Mb window.
(PDF)

Text S2 **The Two Point Correlation Functions of CGI in All Human Chromosomes.** As in Figure 2, the TPCF points and standard errors are given in black for the Takai and Jones CGI, red for the Irizarry et al [56] CGI, and green for the Illingworth et al [40] CGI. Best-fit power laws are over-plotted, and the best-fit power law amplitude and index are given in the legend, all using the same color scheme. Note that the fitting procedure converges for all but the CGIs from Illingworth et al [40] on the Y-chromosome, where it fails to locate a stable minimum. Thus the fit parameters are omitted in the legend in this single instance.
(PDF)
