# Supplementary material for: Long-Range Autocorrelations of CpG Islands in the Human Genome
Source: PLoS One. 2012 Jan 11;7(1):e29889. doi: 10.1371/journal.pone.0029889 (PMC3256200; doi:10.1371/journal.pone.0029889)

Fig S1a. Density plot of Chromosome 1

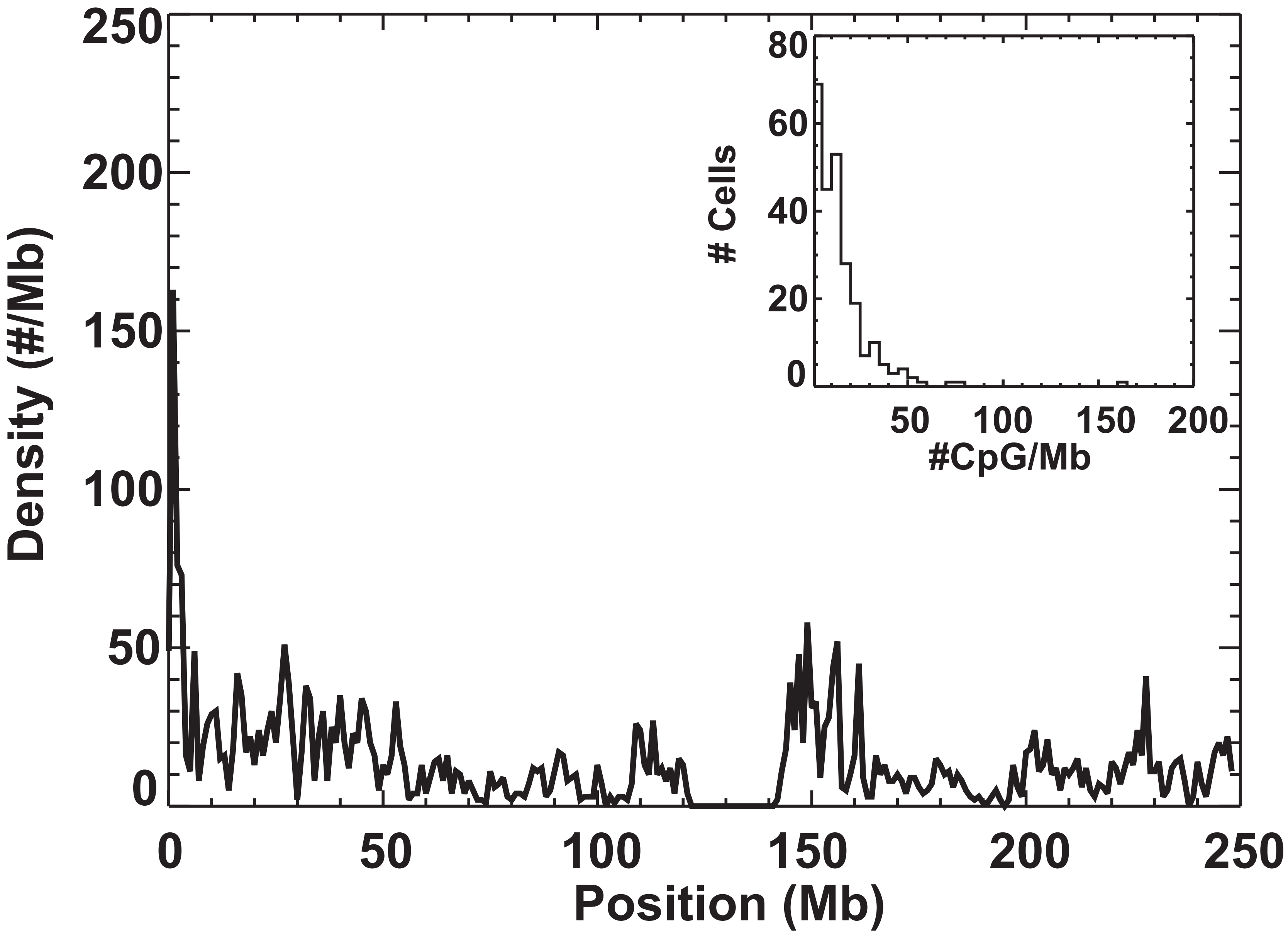

Fig S1b. Density plot of Chromosome 2

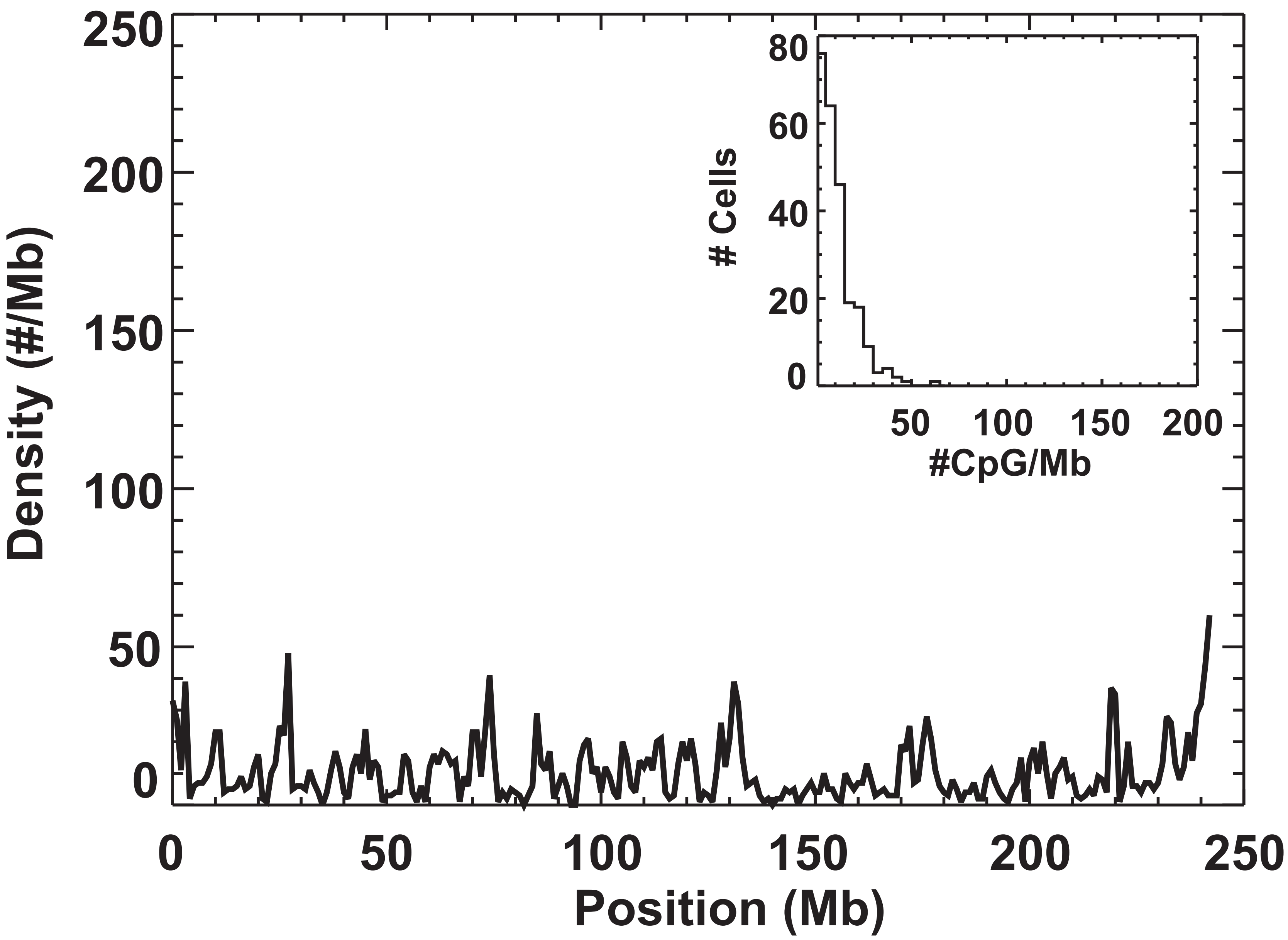

Fig S1c. Density plot of Chromosome 3

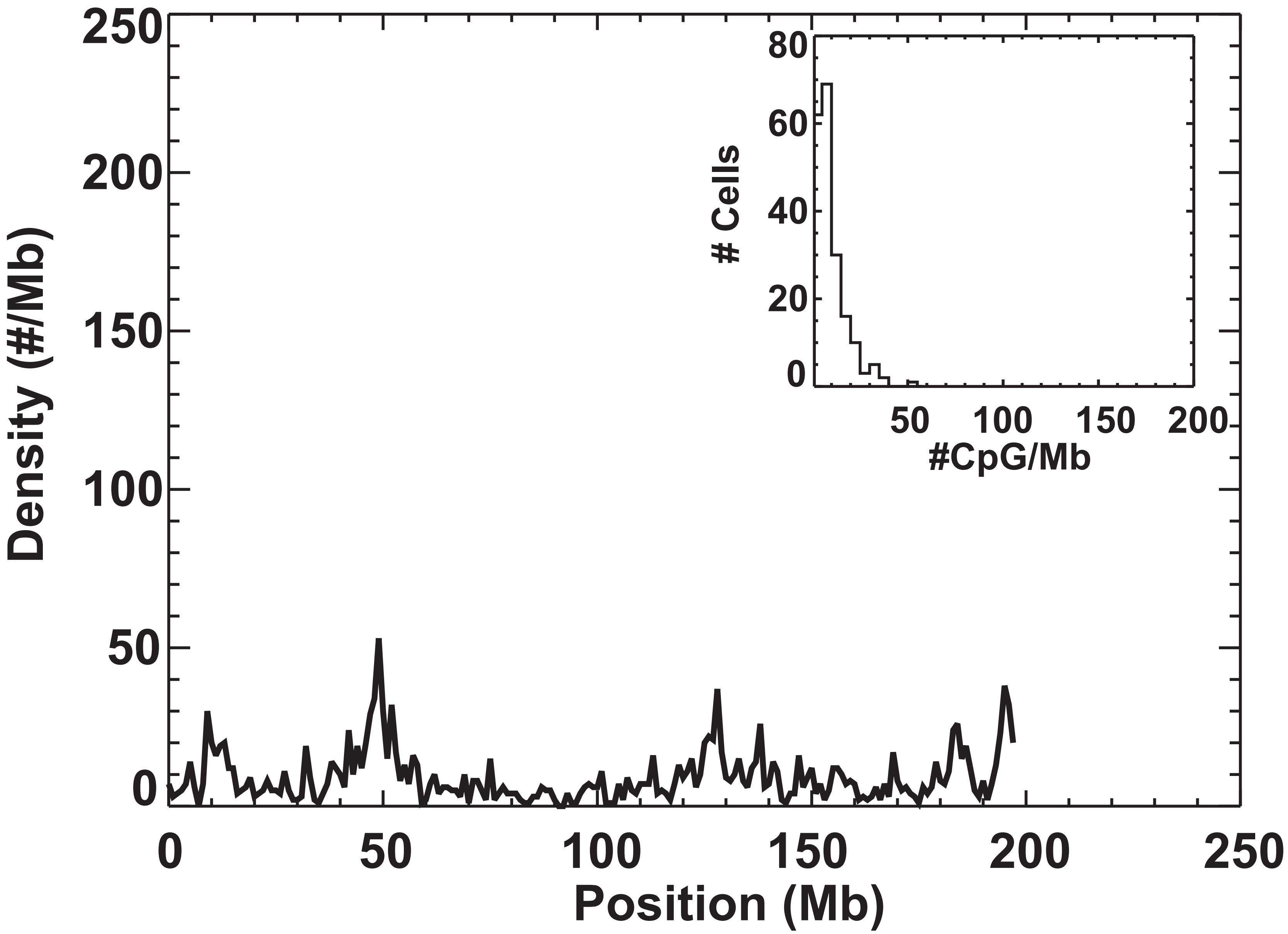

Fig S1d. Density plot of Chromosome 4

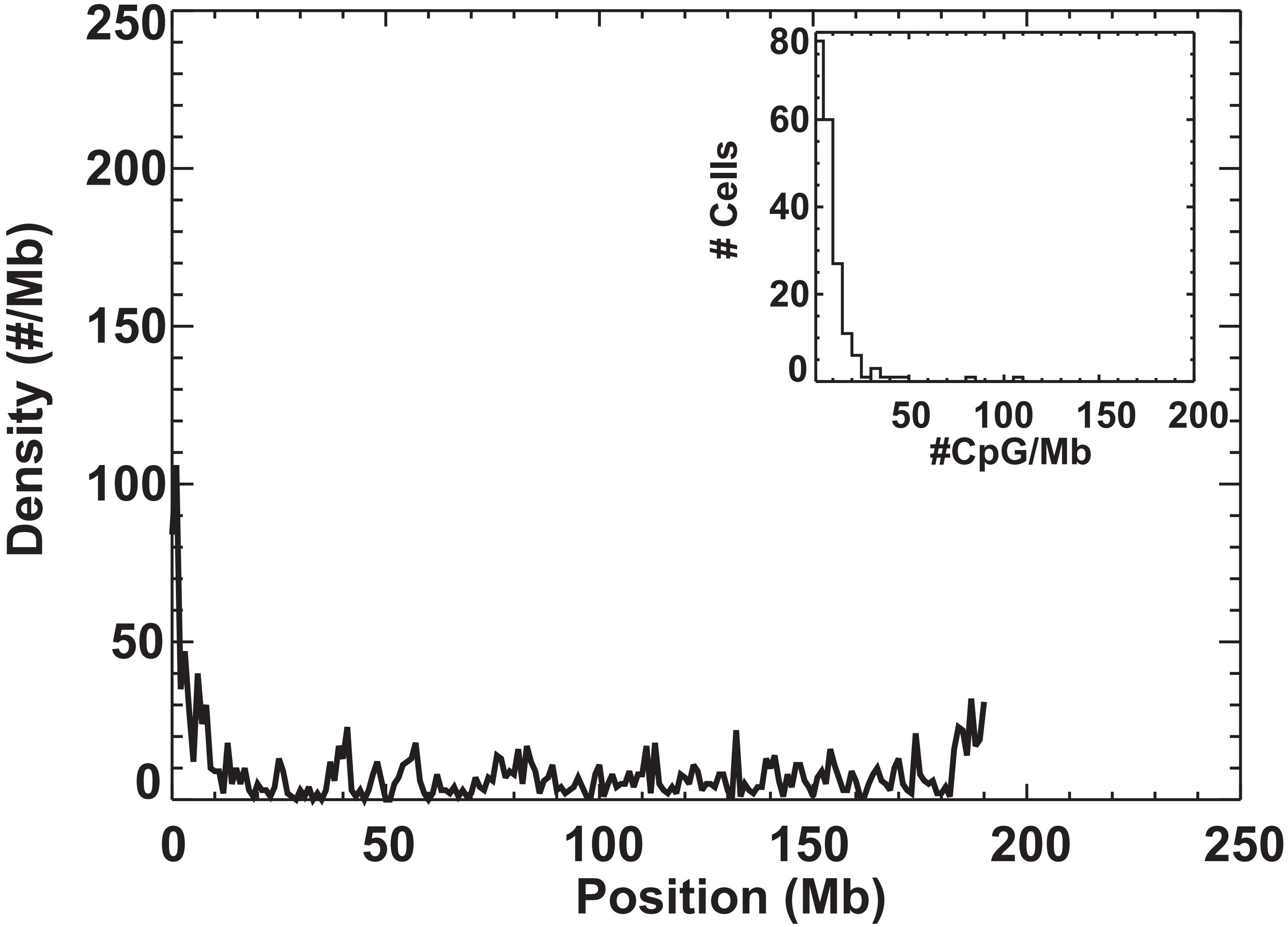

Fig S1e. Density plot of Chromosome 5

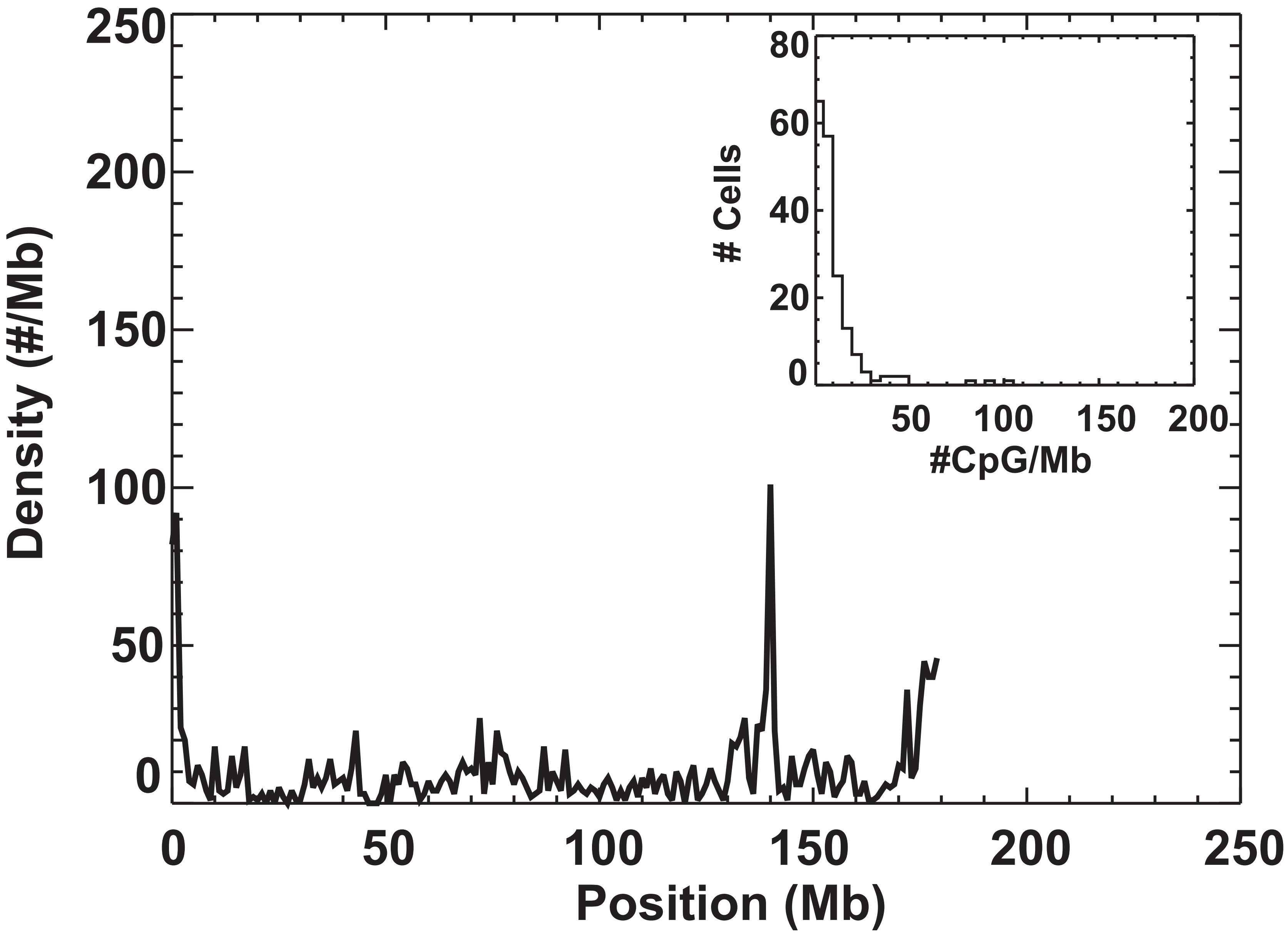

Fig S1f. Density plot of Chromosome 6

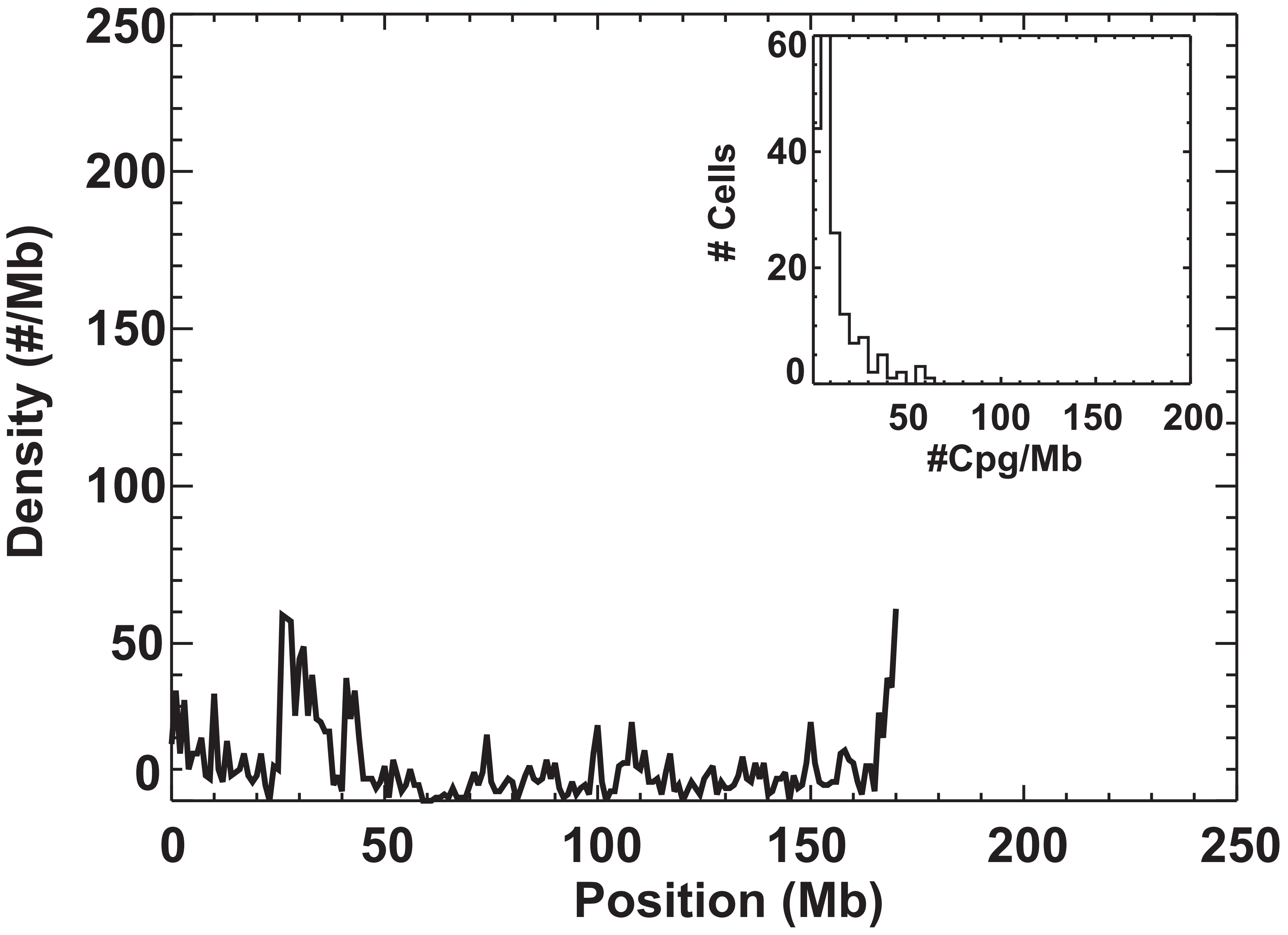

Fig S1g. Density plot of Chromosome 7

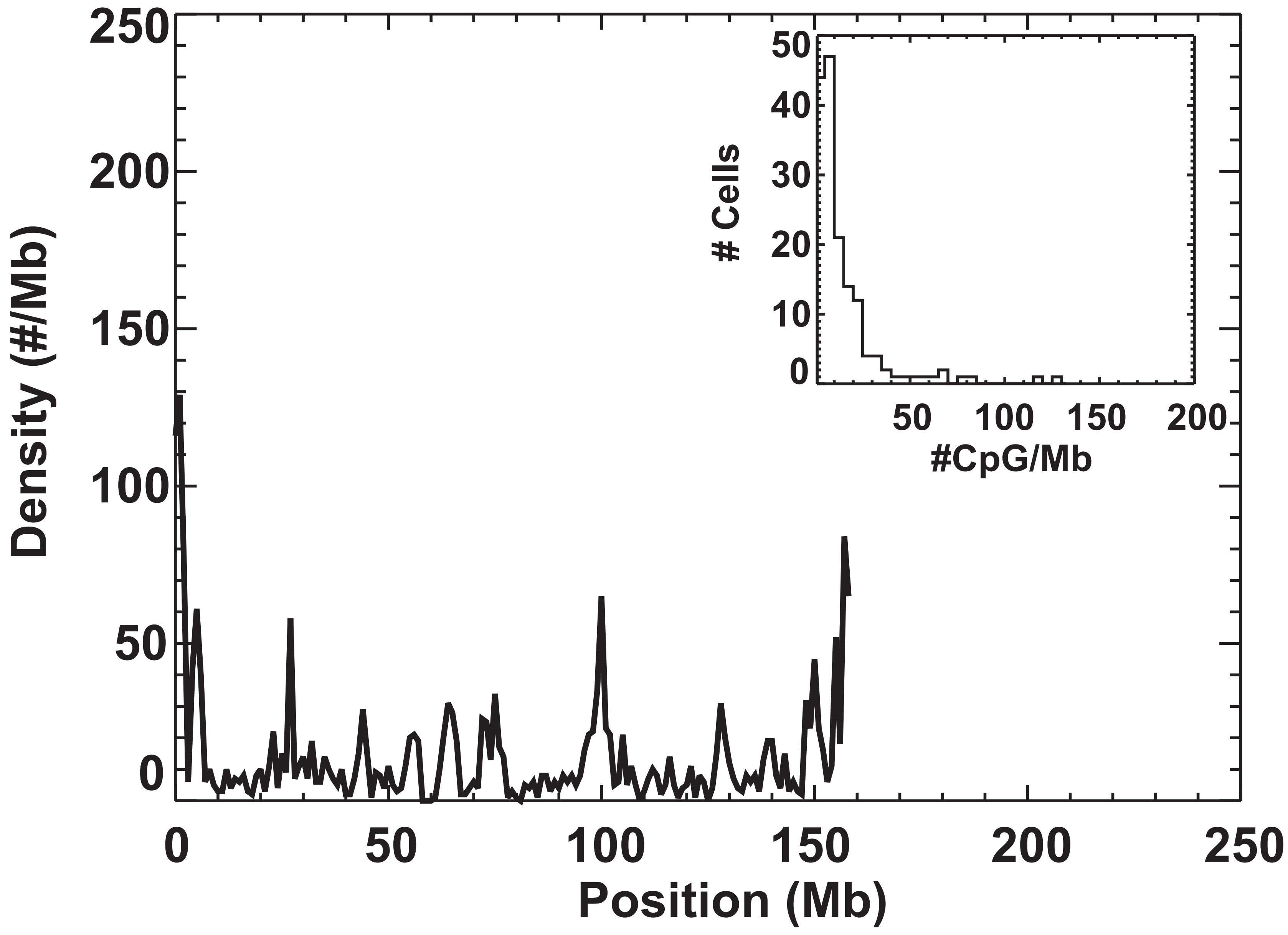

Fig S1h. Density plot of Chromosome 8

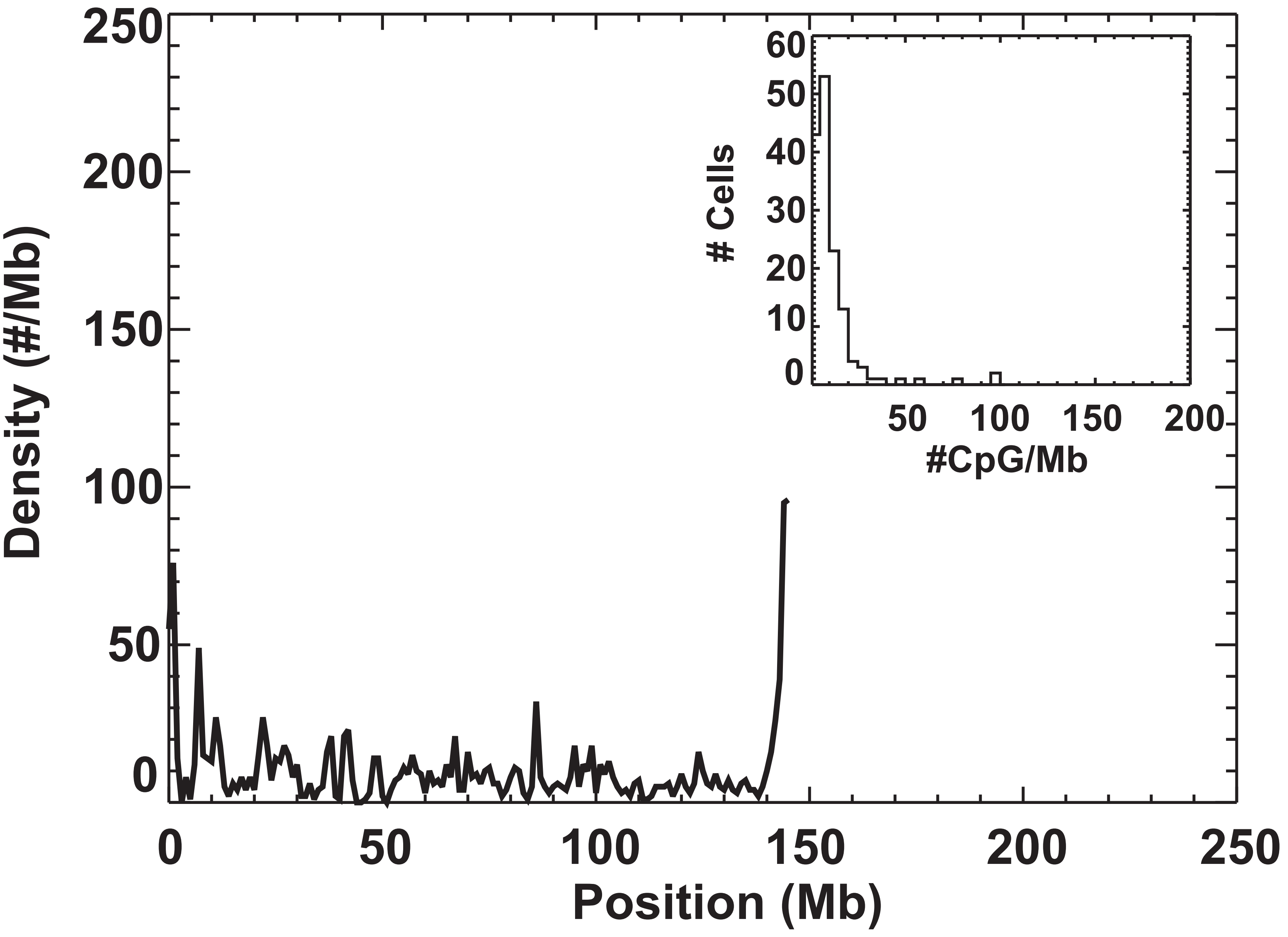

Fig S1i. Density plot of Chromosome 9

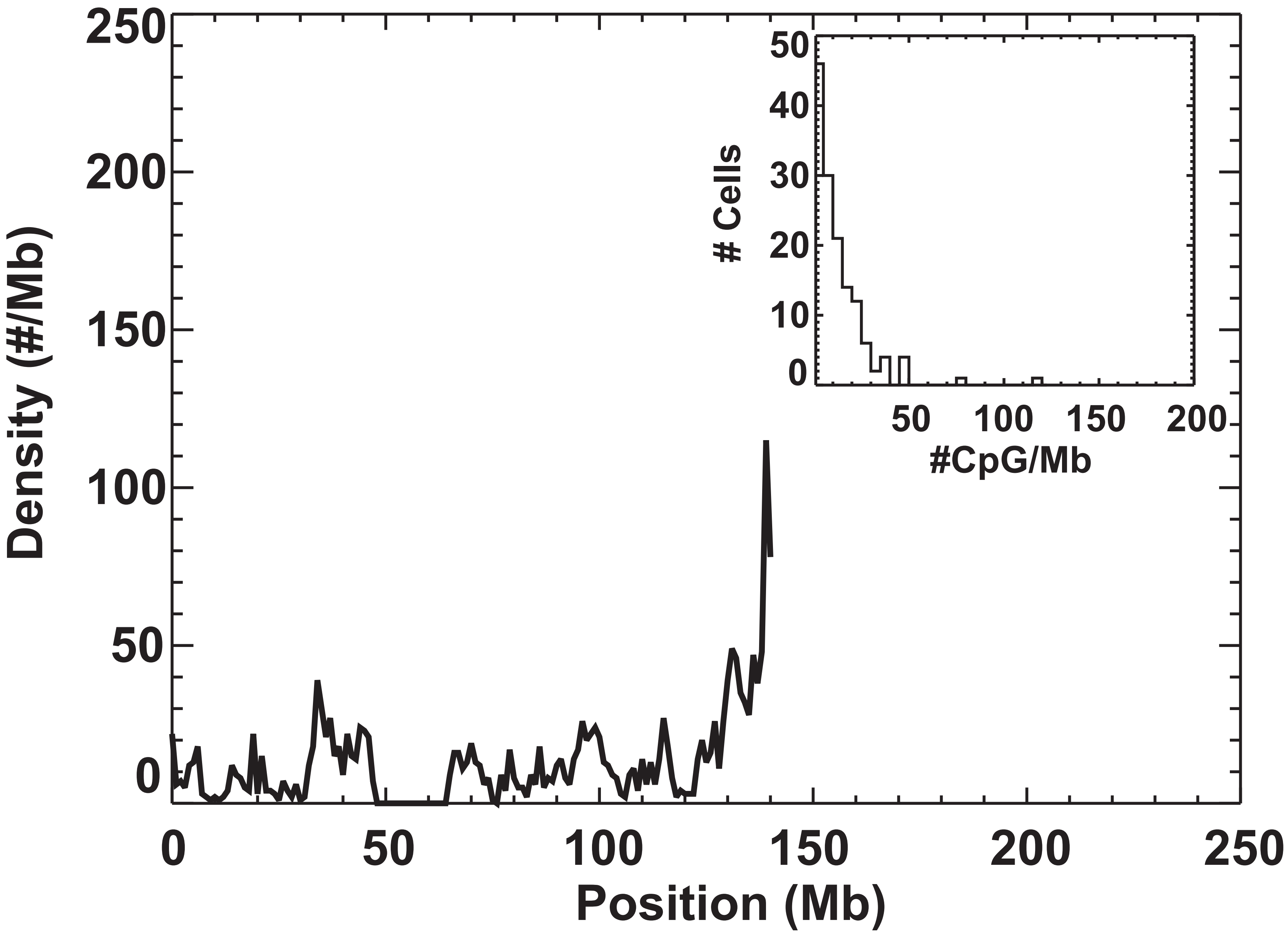

Fig S1j. Density plot of Chromosome 10

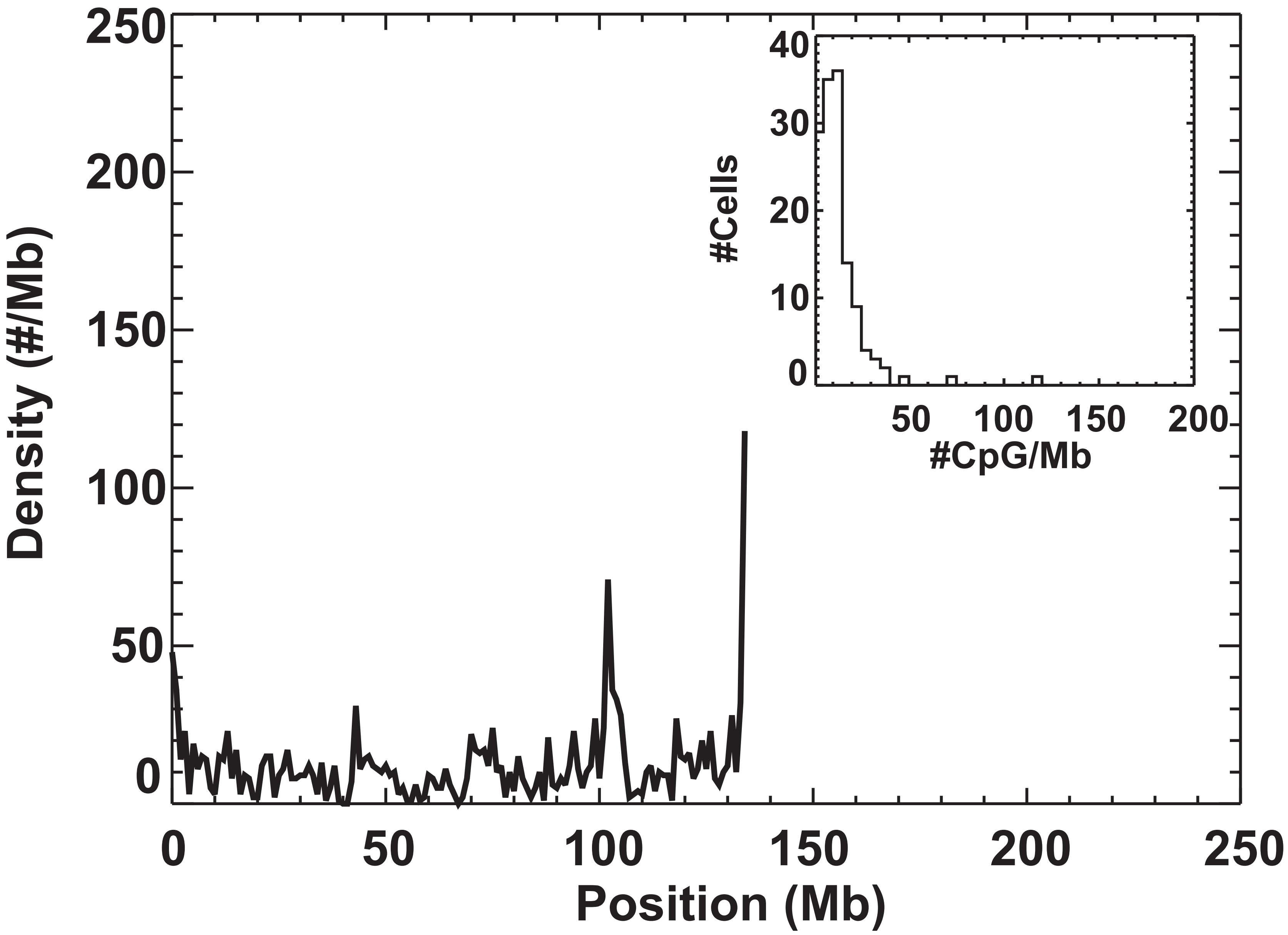

Fig S1k. Density plot of Chromosome 11

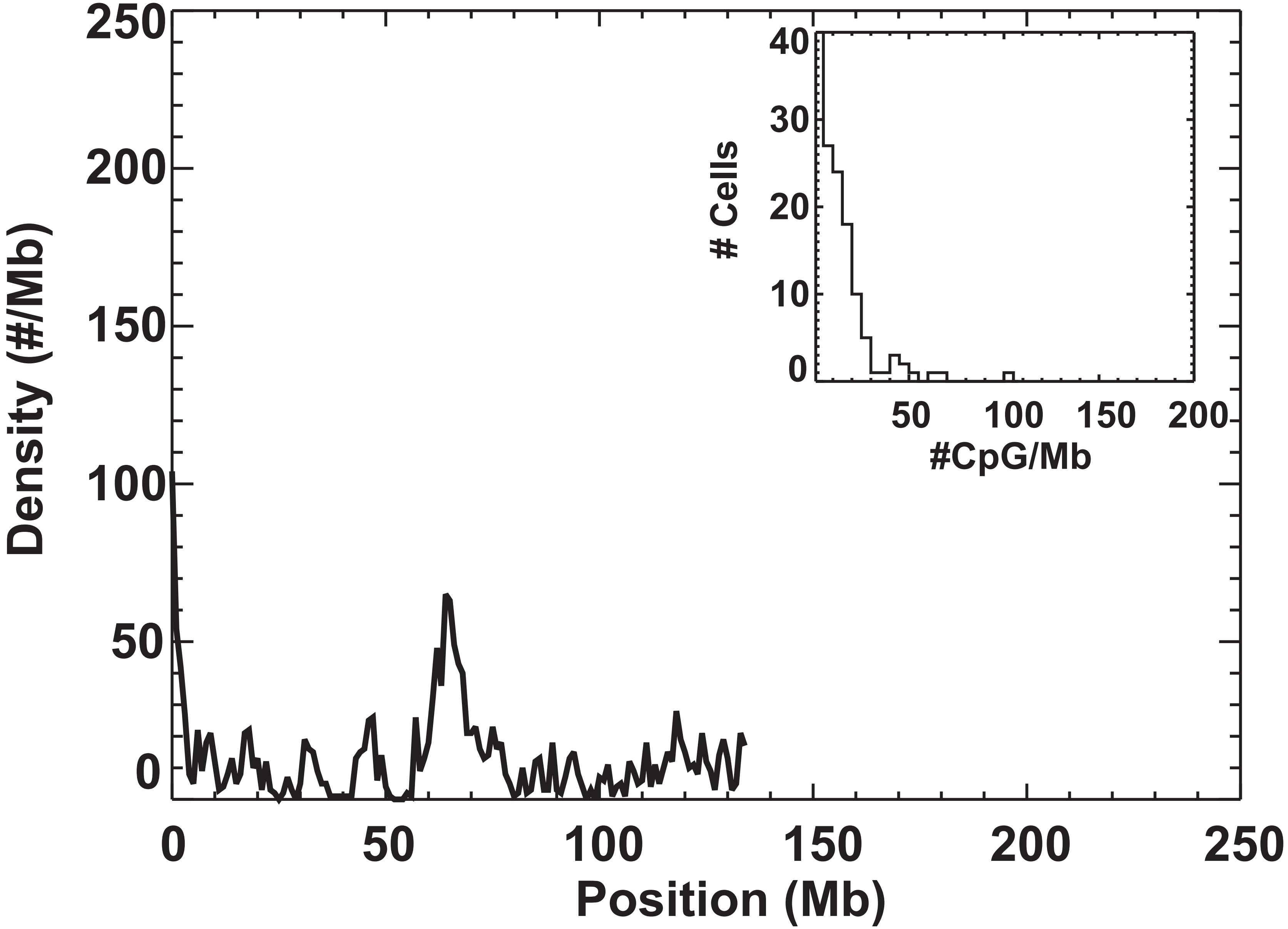

Fig S1I. Density plot of Chromosome 12

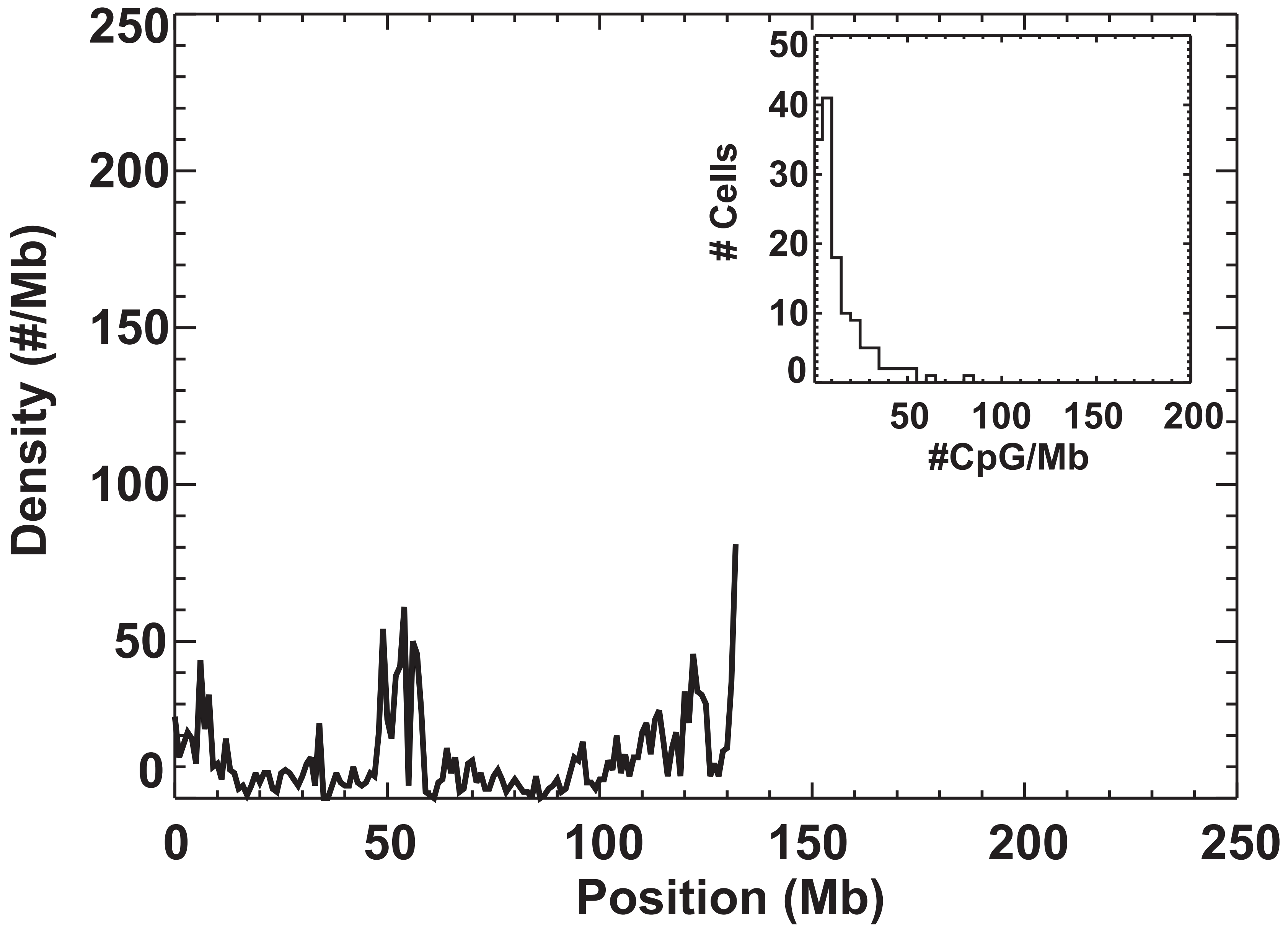

Fig S1m. Density plot of Chromosome 13

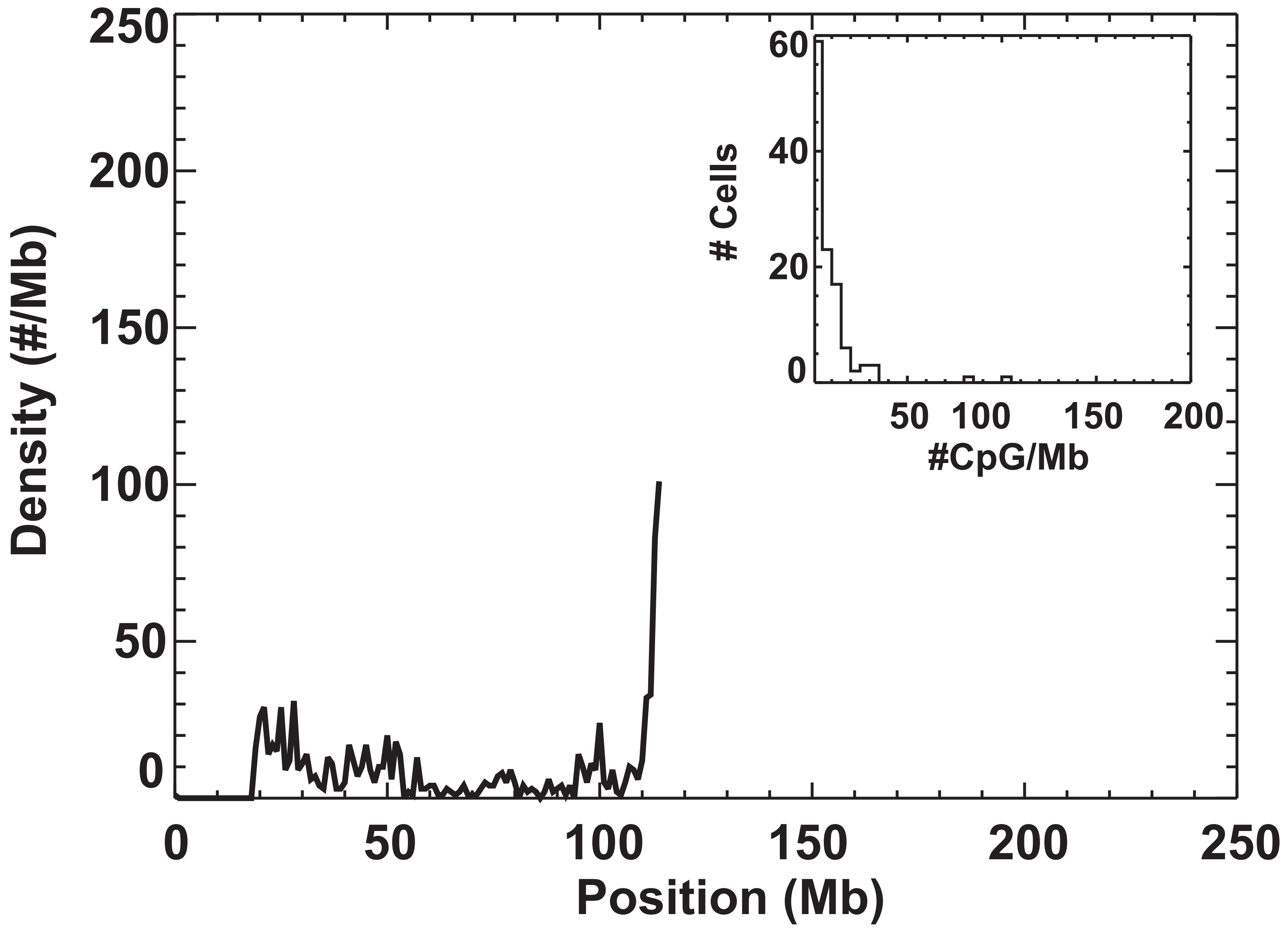

Fig S1n. Density plot of Chromosome 14

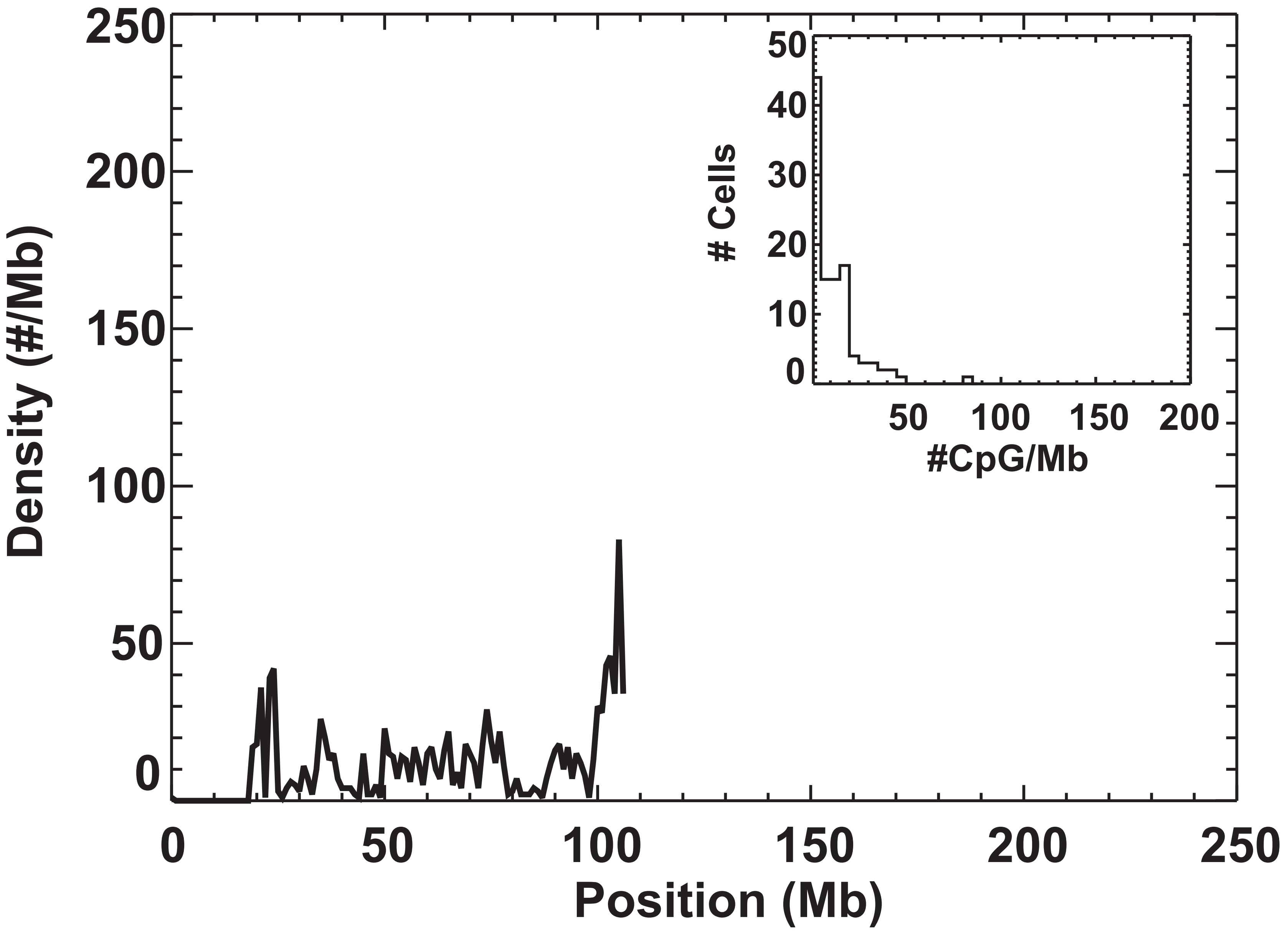

Fig S1o. Density plot of Chromosome 15

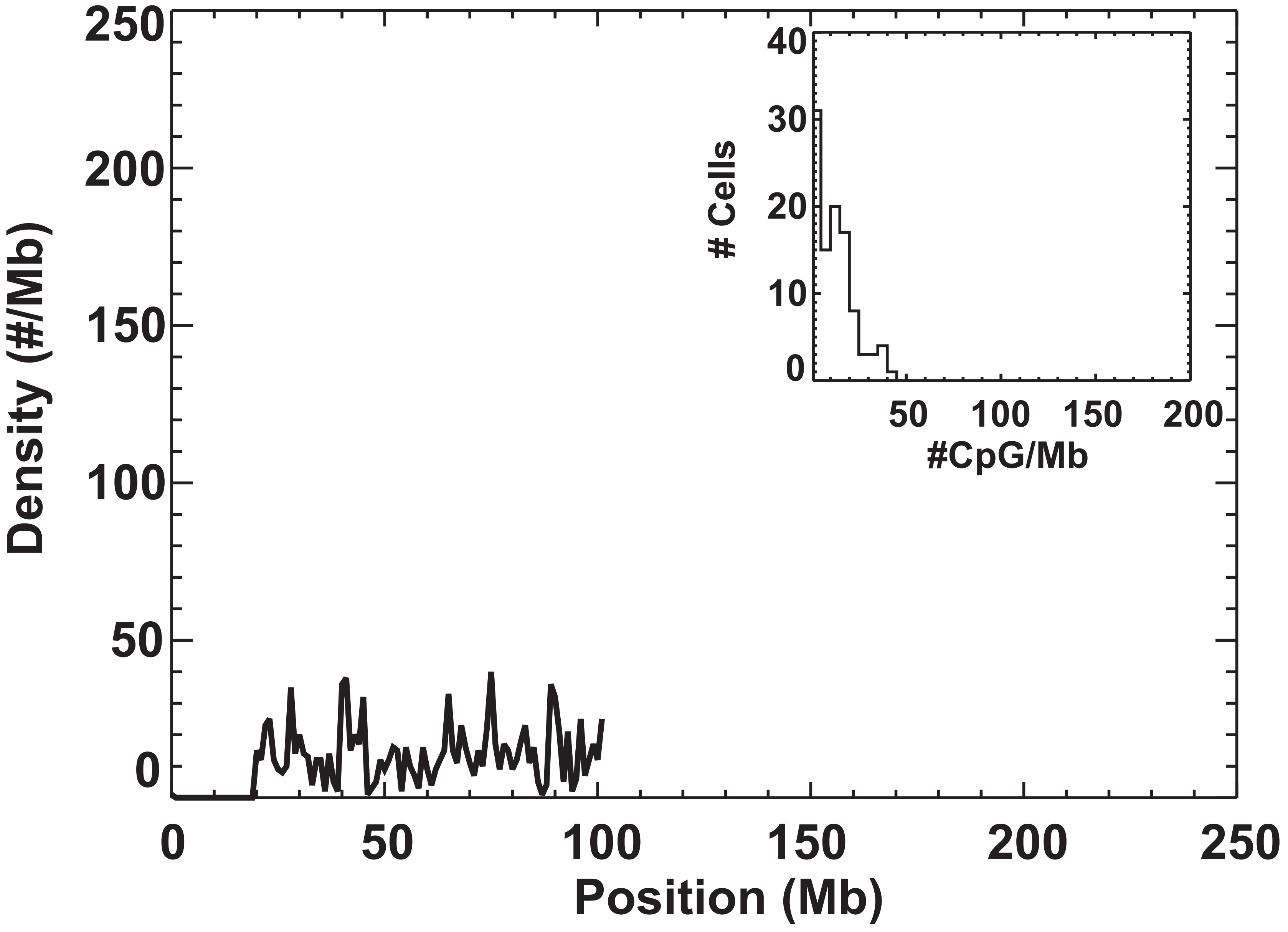

Fig S1p. Density plot of Chromosome 16

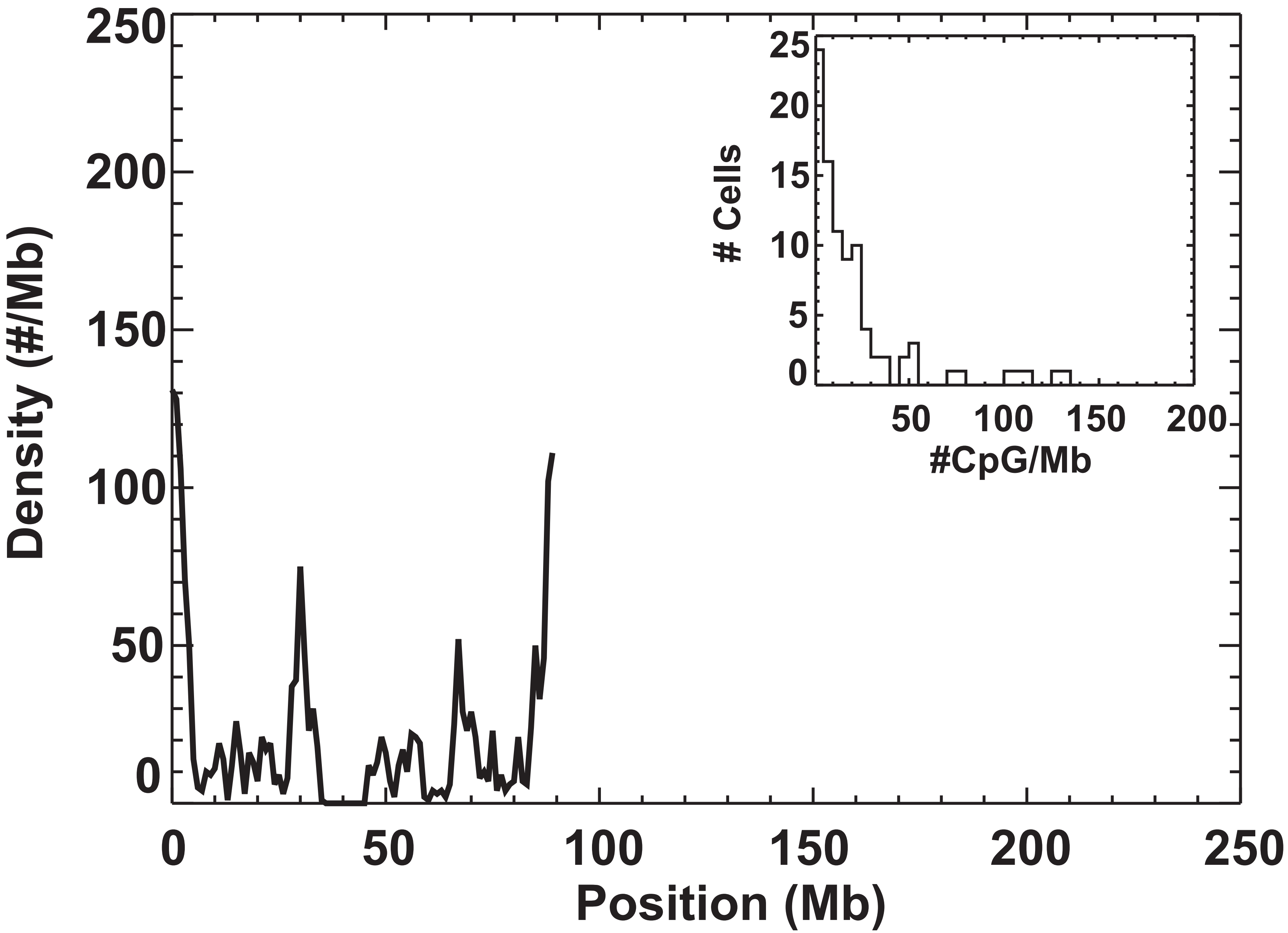

Fig S1q. Density plot of Chromosome 17

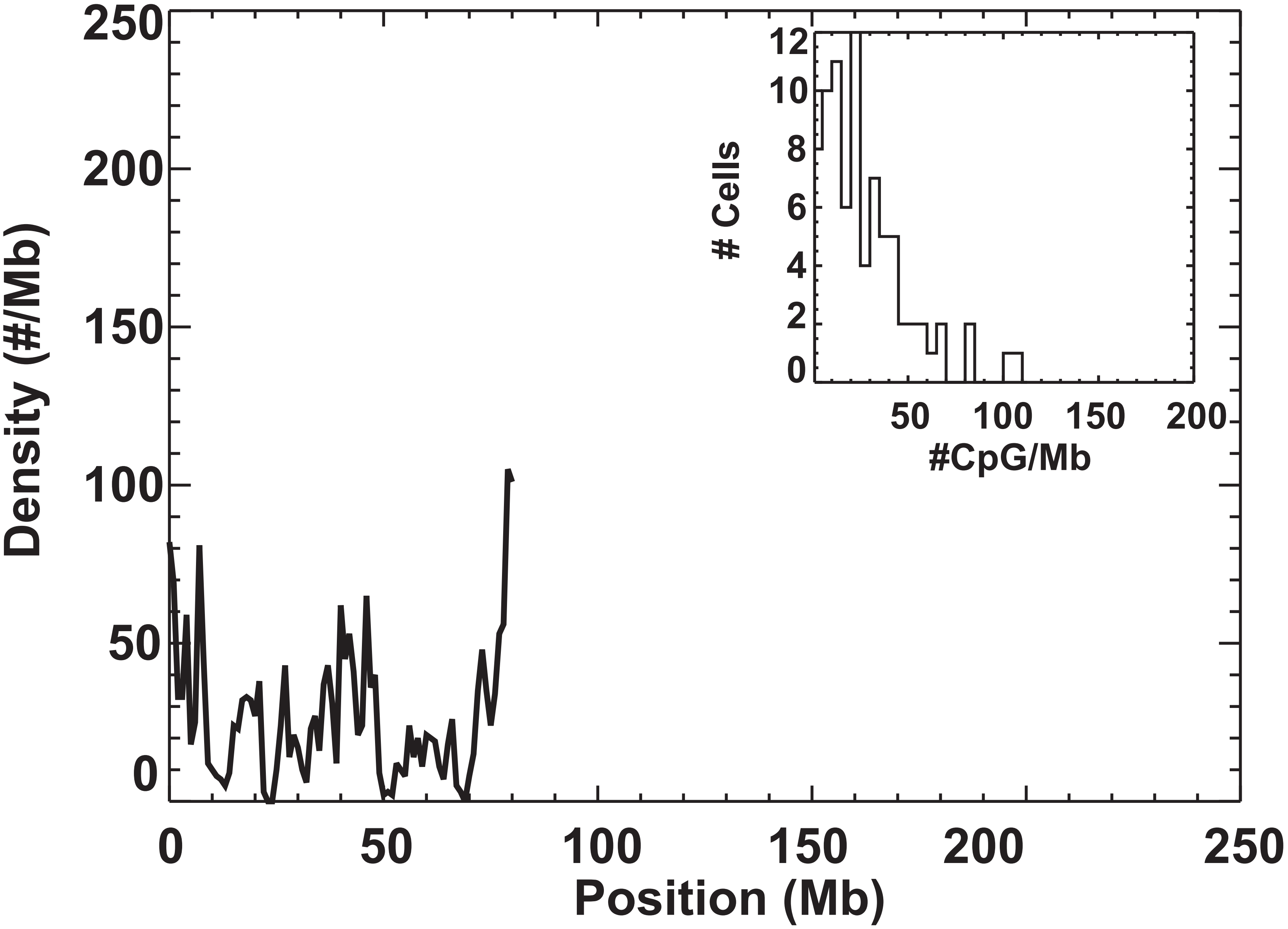

Fig S1r. Density plot of Chromosome 18

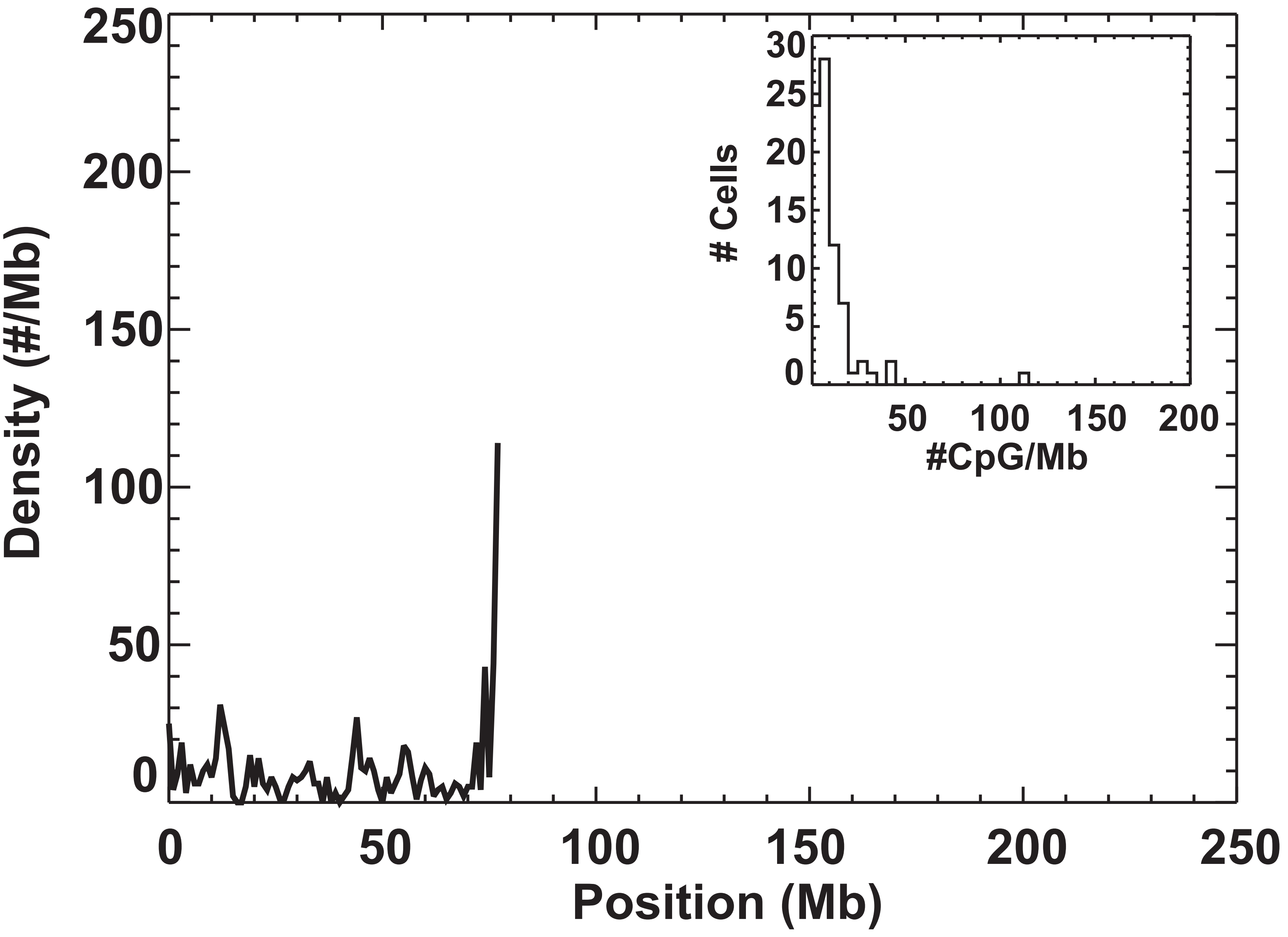

Fig S1s. Density plot of Chromosome 19

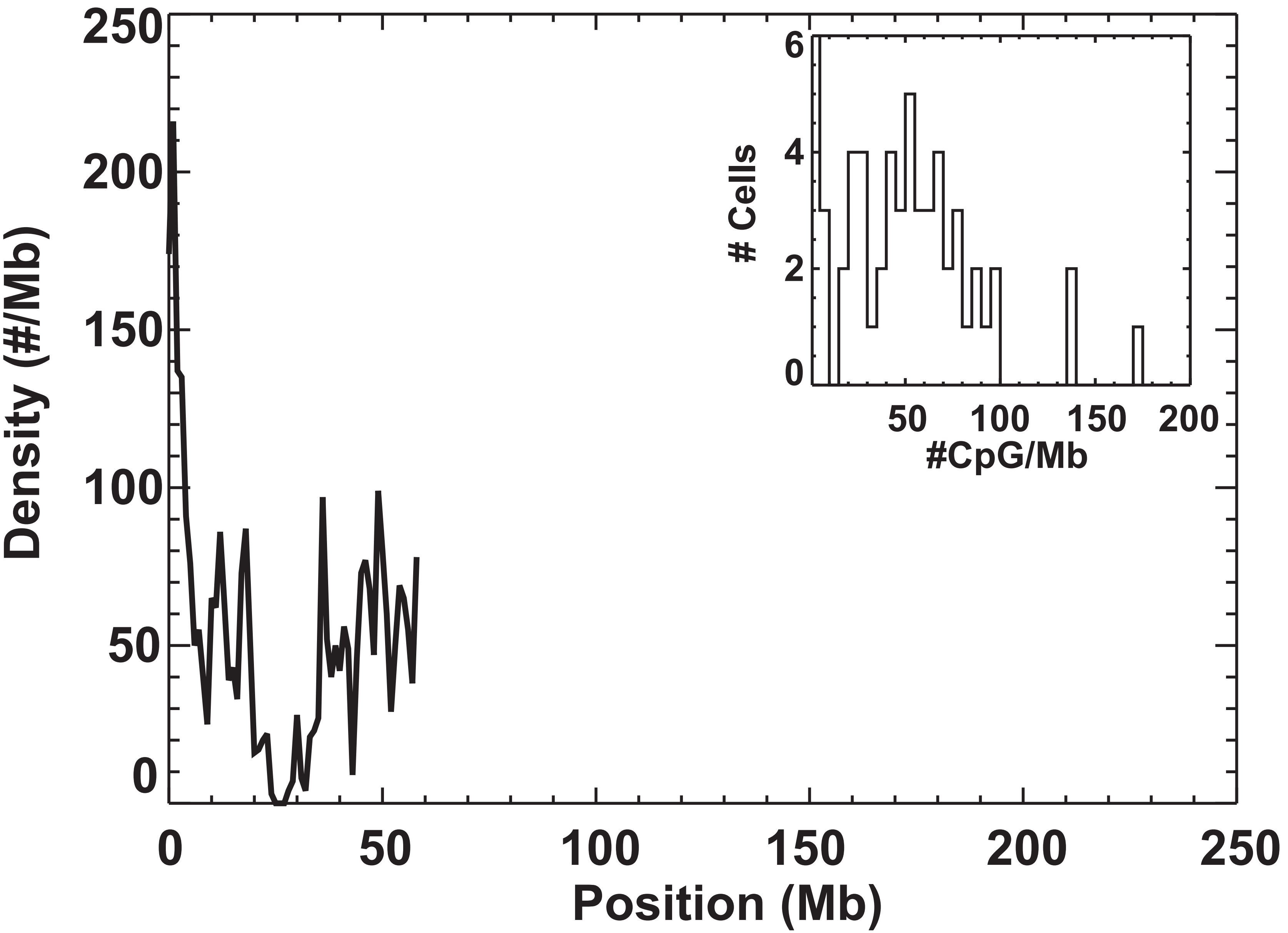

Fig S1t. Density plot of Chromosome 20

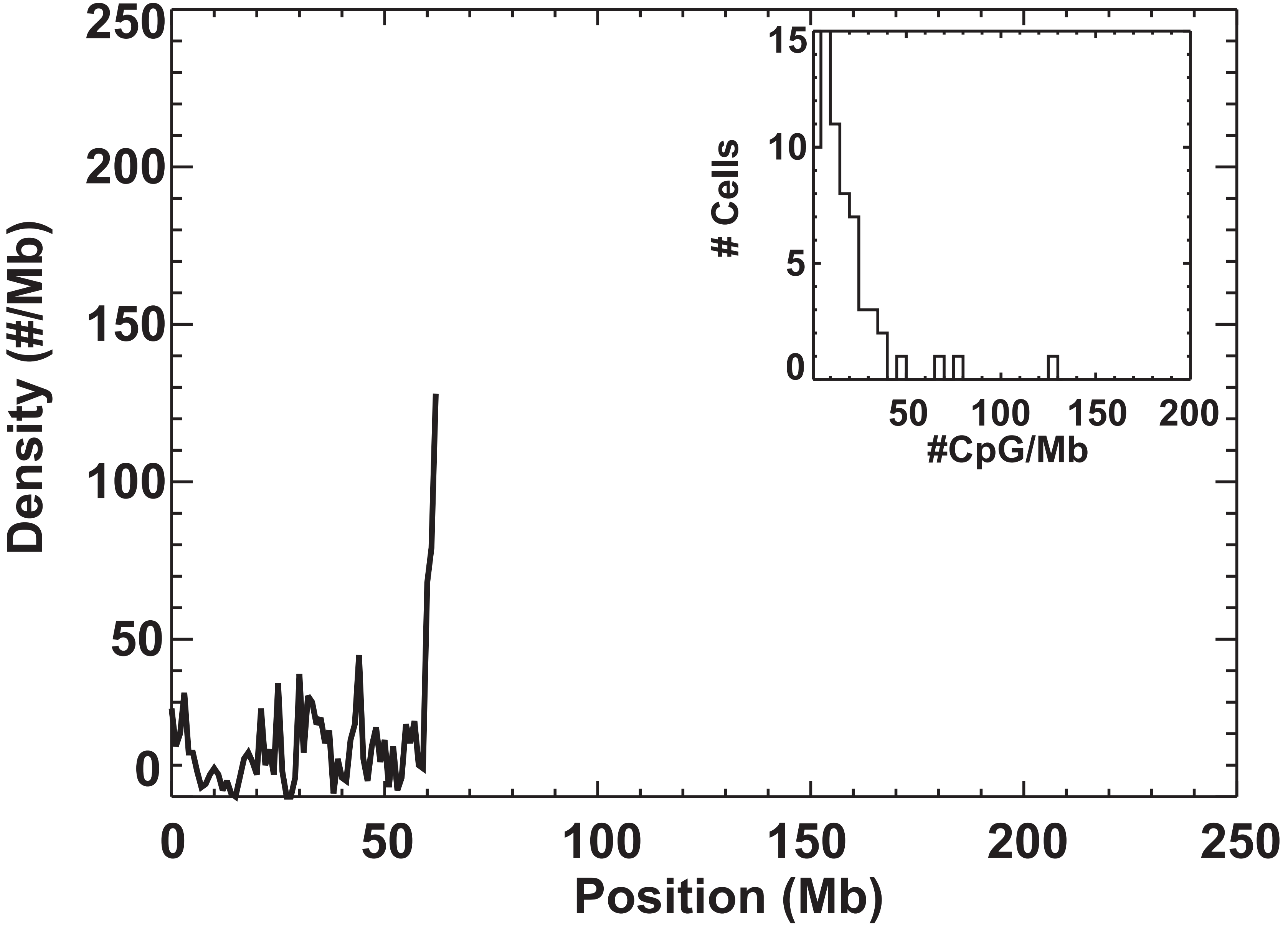

Fig S1u. Density plot of Chromosome 21

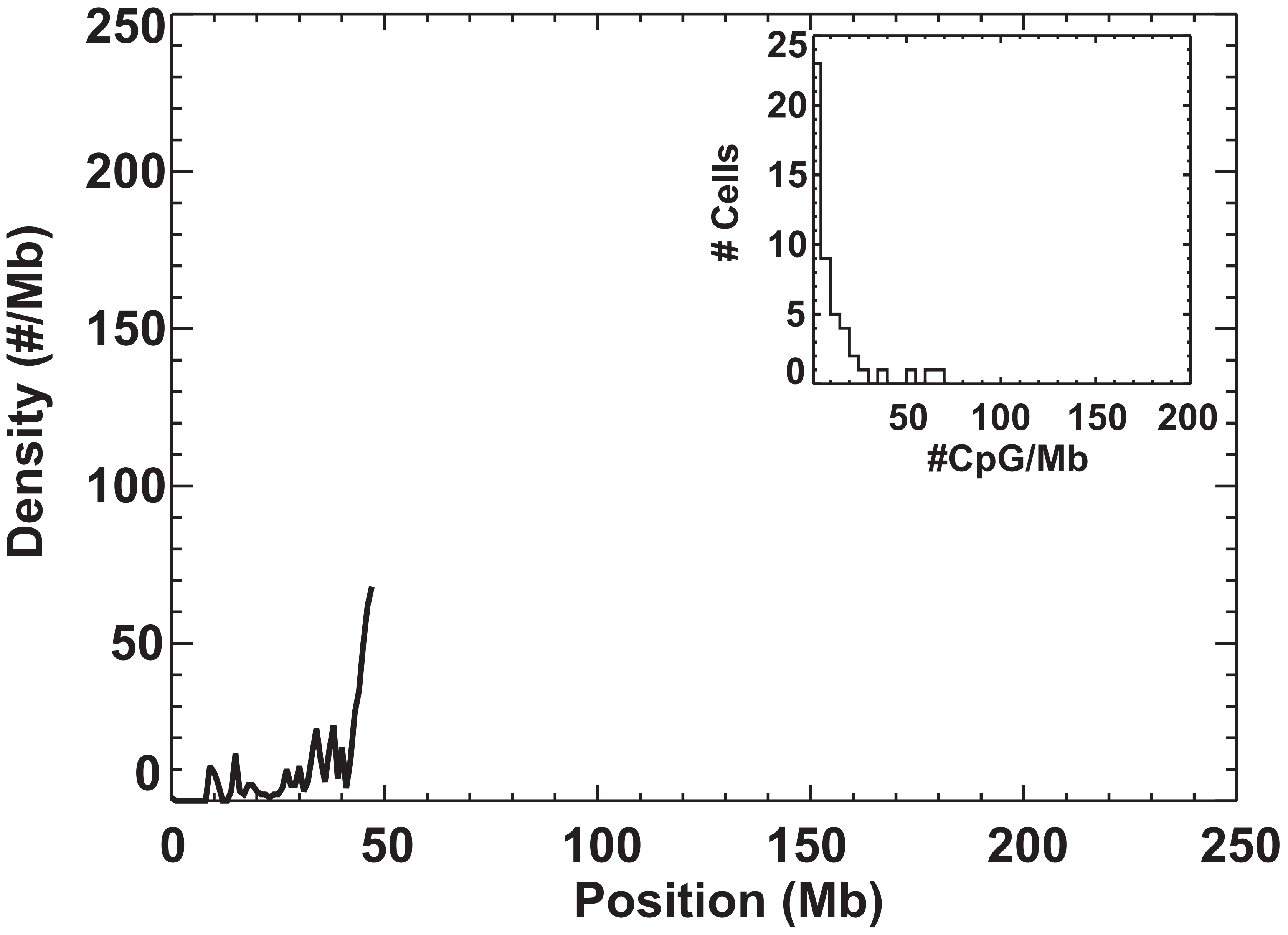

Fig S1v. Density plot of Chromosome 22

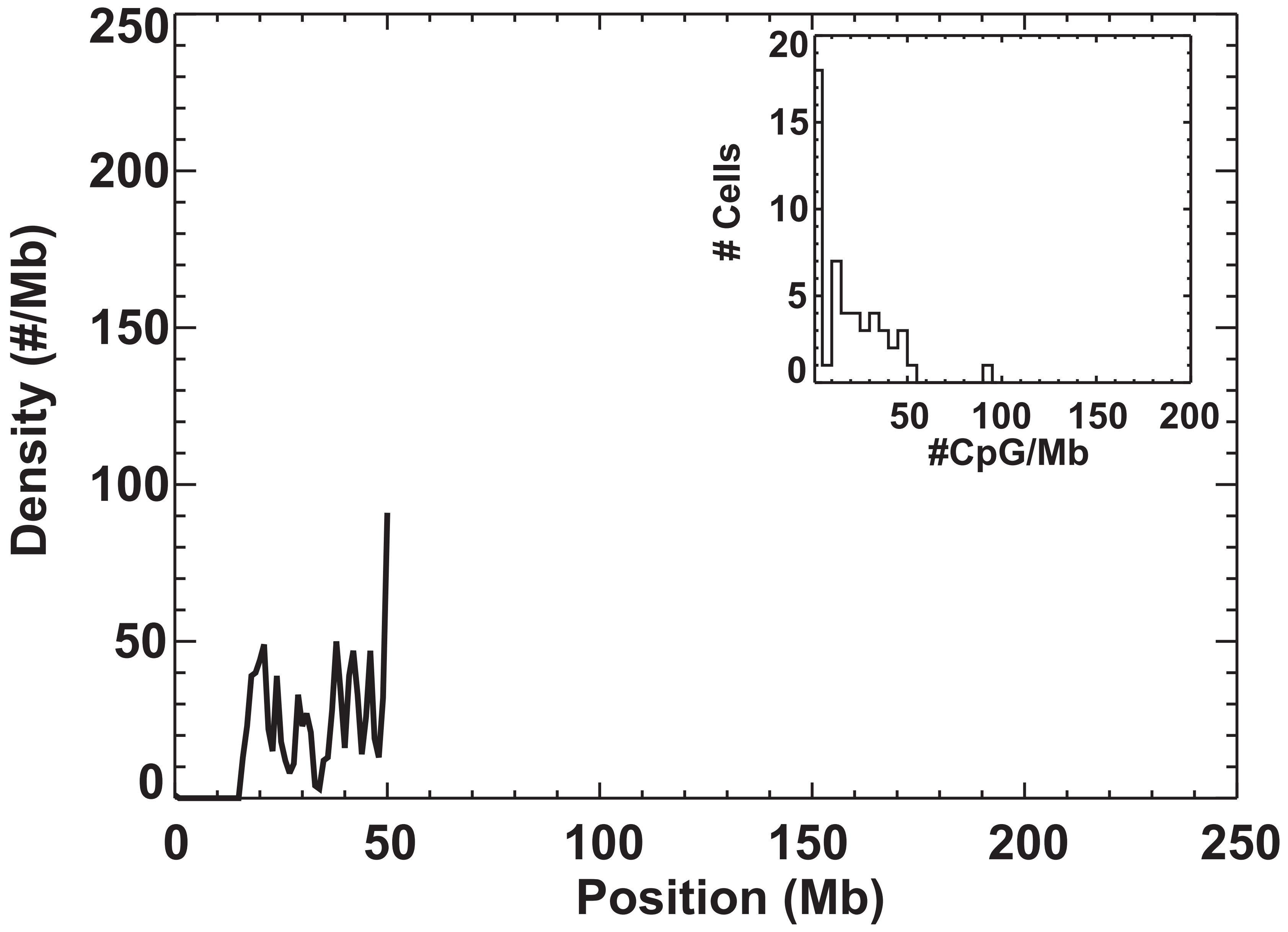

Fig S1w. Density plot of Chromosome X

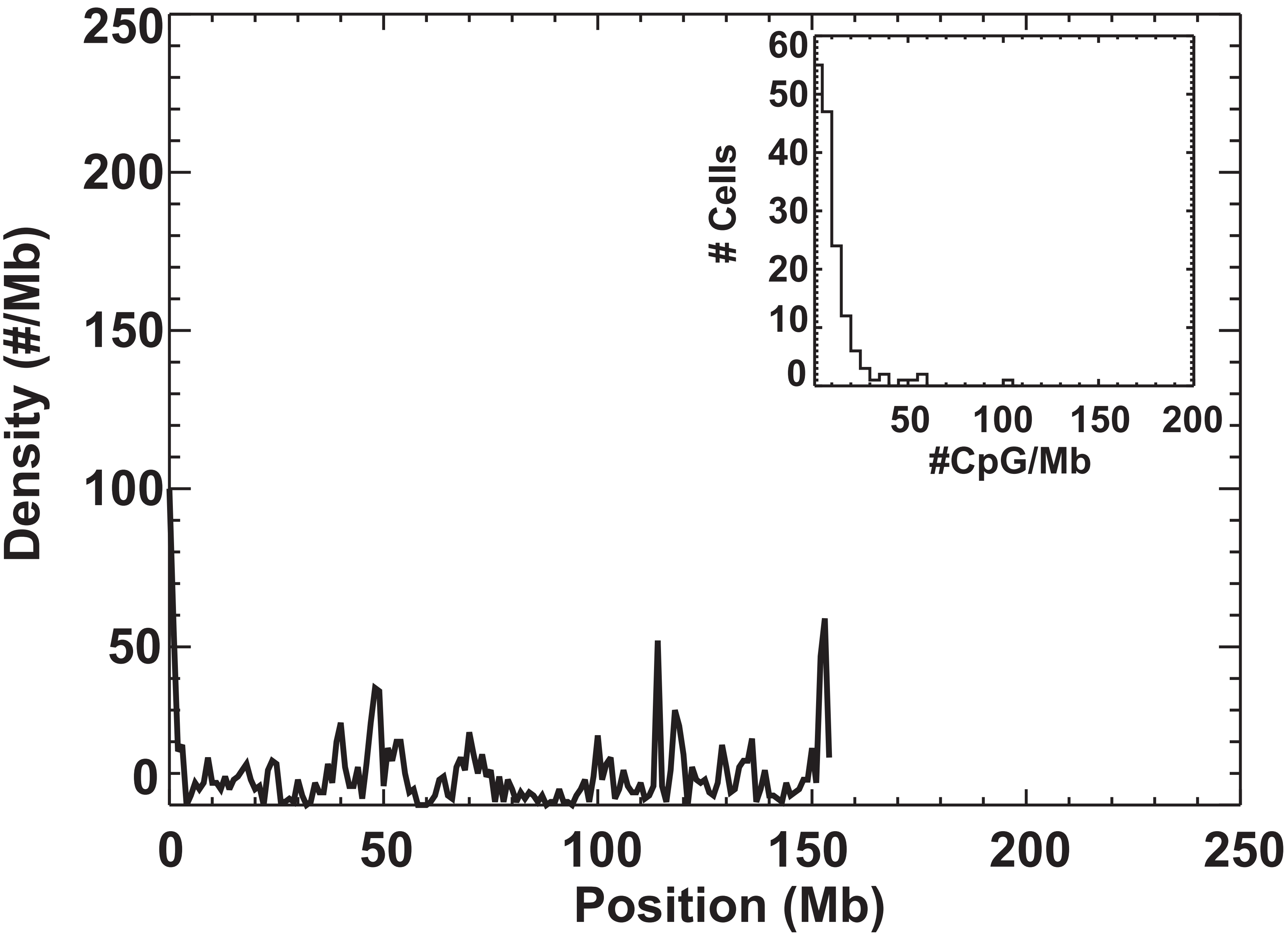

Fig S1x. Density plot of Chromosome Y

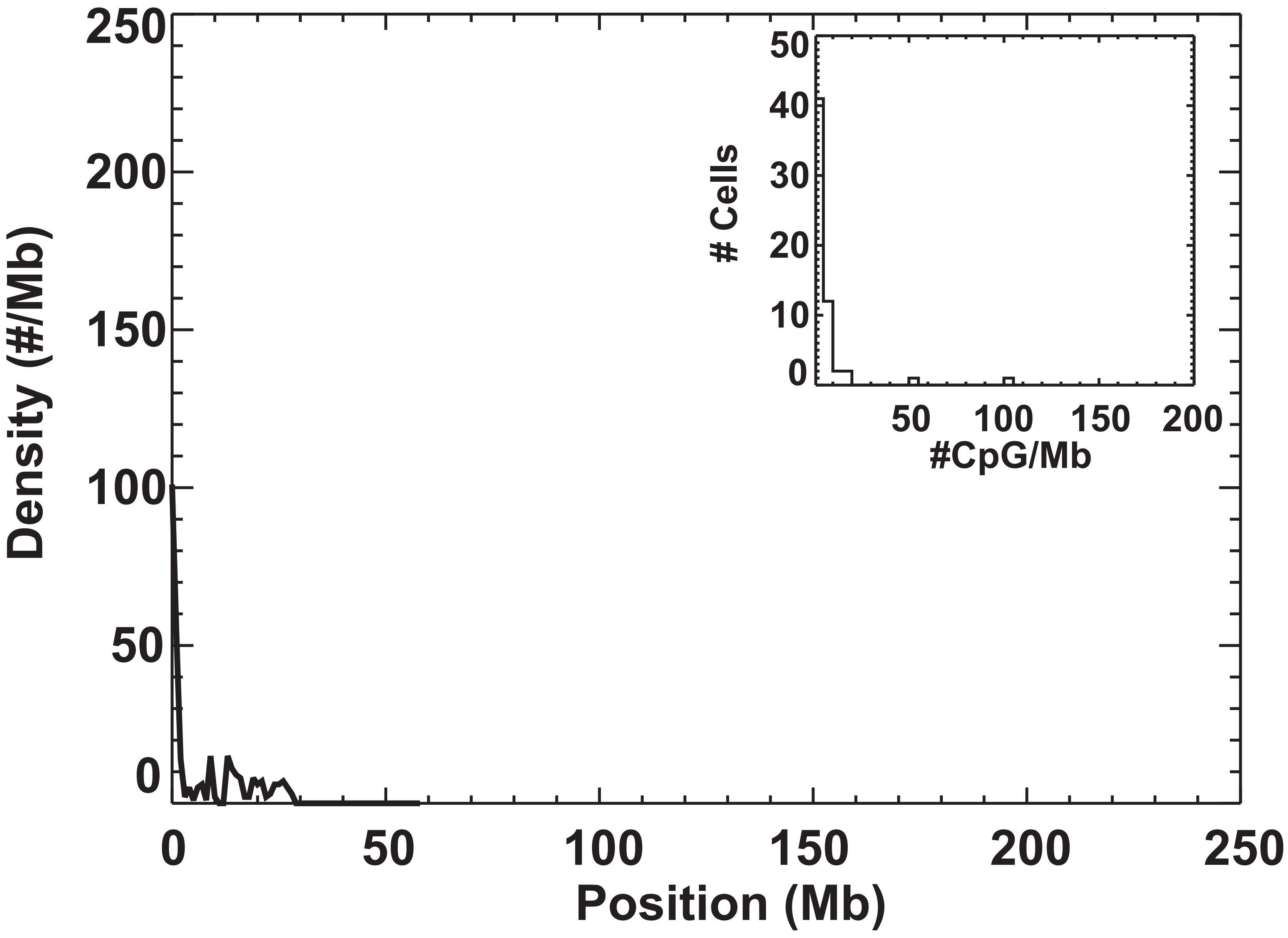

Supplement: Text S1 — Density plots of CGI for All Human Chromosomes. As in Figure 1, density is simply defined as the number of Takai and Jones CGI per non-overlapping 1 Mb window. (PDF) [file pone.0029889.s001.pdf]
