# Supplementary material for: Long-Range Autocorrelations of CpG Islands in the Human Genome
Source: PLoS One. 2012 Jan 11;7(1):e29889. doi: 10.1371/journal.pone.0029889 (PMC3256200; doi:10.1371/journal.pone.0029889)
Supplement: Text S2 — The Two Point Correlation Functions of CGI in All Human Chromosomes. As in Figure 2, the TPCF points and standard errors are given in black for the Takai and Jones CGI, red for the Irizarry et al [56] CGI, and green for the Illingworth et al [40] CGI. Best-fit power laws are over-plotted, and the best-fit power law amplitude and index are given in the legend, all using the same color scheme. Note that the fitting procedure converges for all but the CGIs from Illingworth et al [40] on the Y-chromosome, where it fails to locate a stable minimum. Thus the fit parameters are omitted in the legend in this single instance. (PDF) [file pone.0029889.s002.pdf]

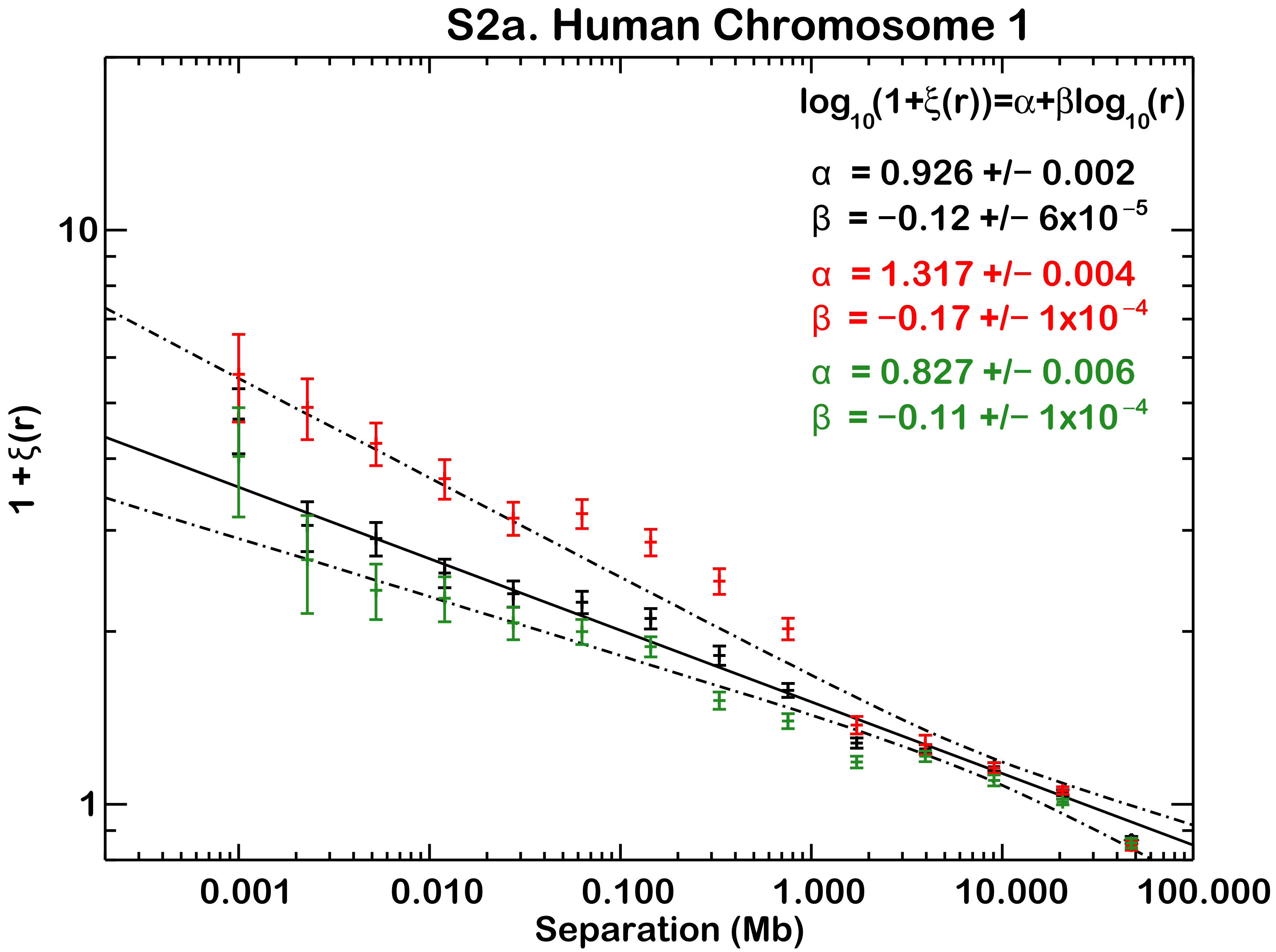

S2a. Human Chromosome 1
log10(1+ξ(r))=α+βlog10(r)
α = 0.926 +/− 0.002
β = −0.12 +/− 6x10−5
α = 1.317 +/− 0.004
β = −0.17 +/− 1x10−4
α = 0.827 +/− 0.006
β = −0.11 +/− 1x10−4
1 + ξ(r)
10
1
0.001
0.010
0.100
1.000
10.000
100.000
Separation (Mb)

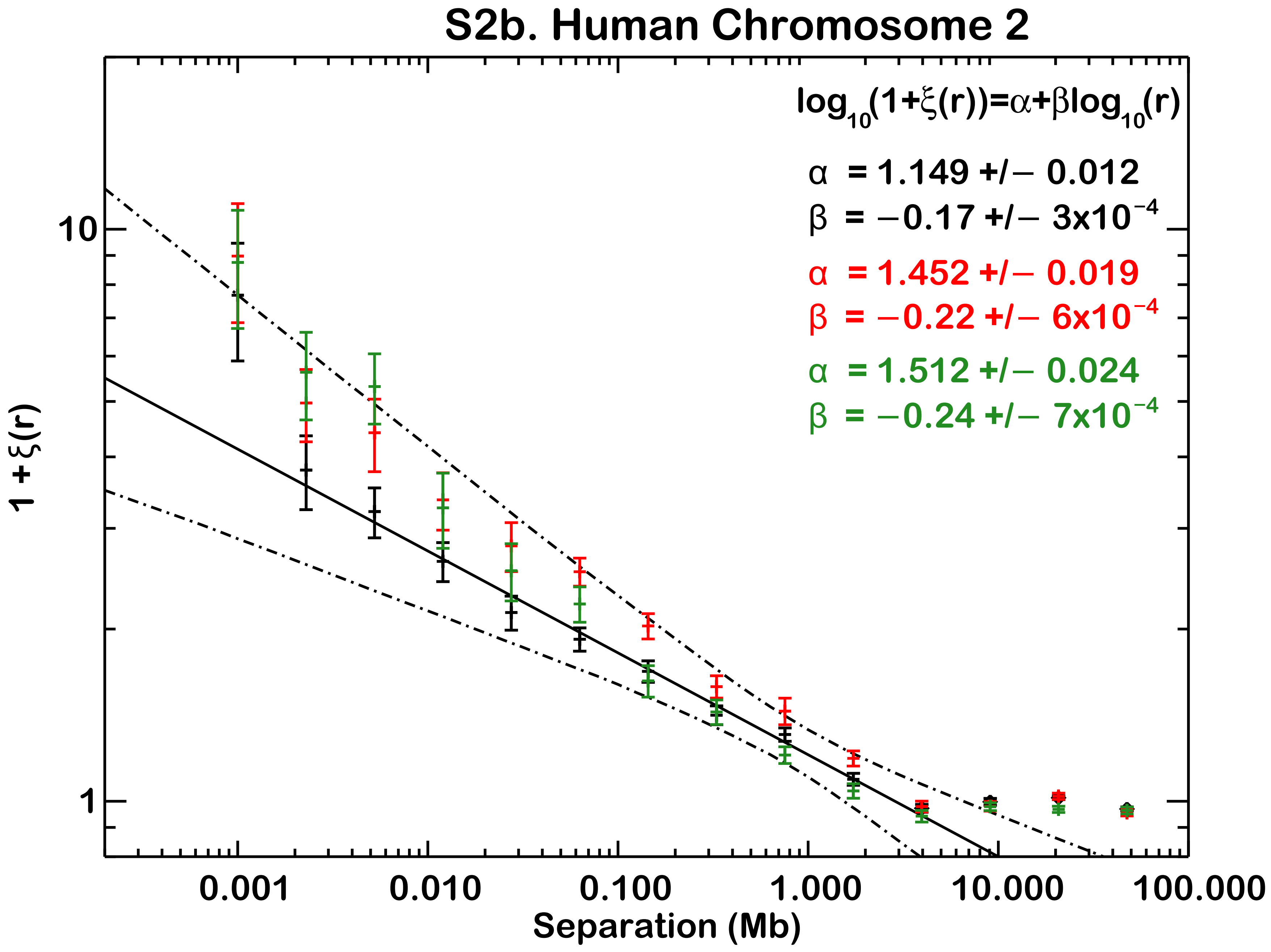

S2b. Human Chromosome 2
$\log_{10}(1+\xi(r))=\alpha+\beta\log_{10}(r)$
α = 1.149 +/− 0.012
β = −0.17 +/− 3x10^{-4}
α = 1.452 +/− 0.019
β = −0.22 +/− 6x10^{-4}
α = 1.512 +/− 0.024
β = −0.24 +/− 7x10^{-4}
1 + ξ(r)
10
1
0.001
0.010
0.100
1.000
10.000
100.000
Separation (Mb)

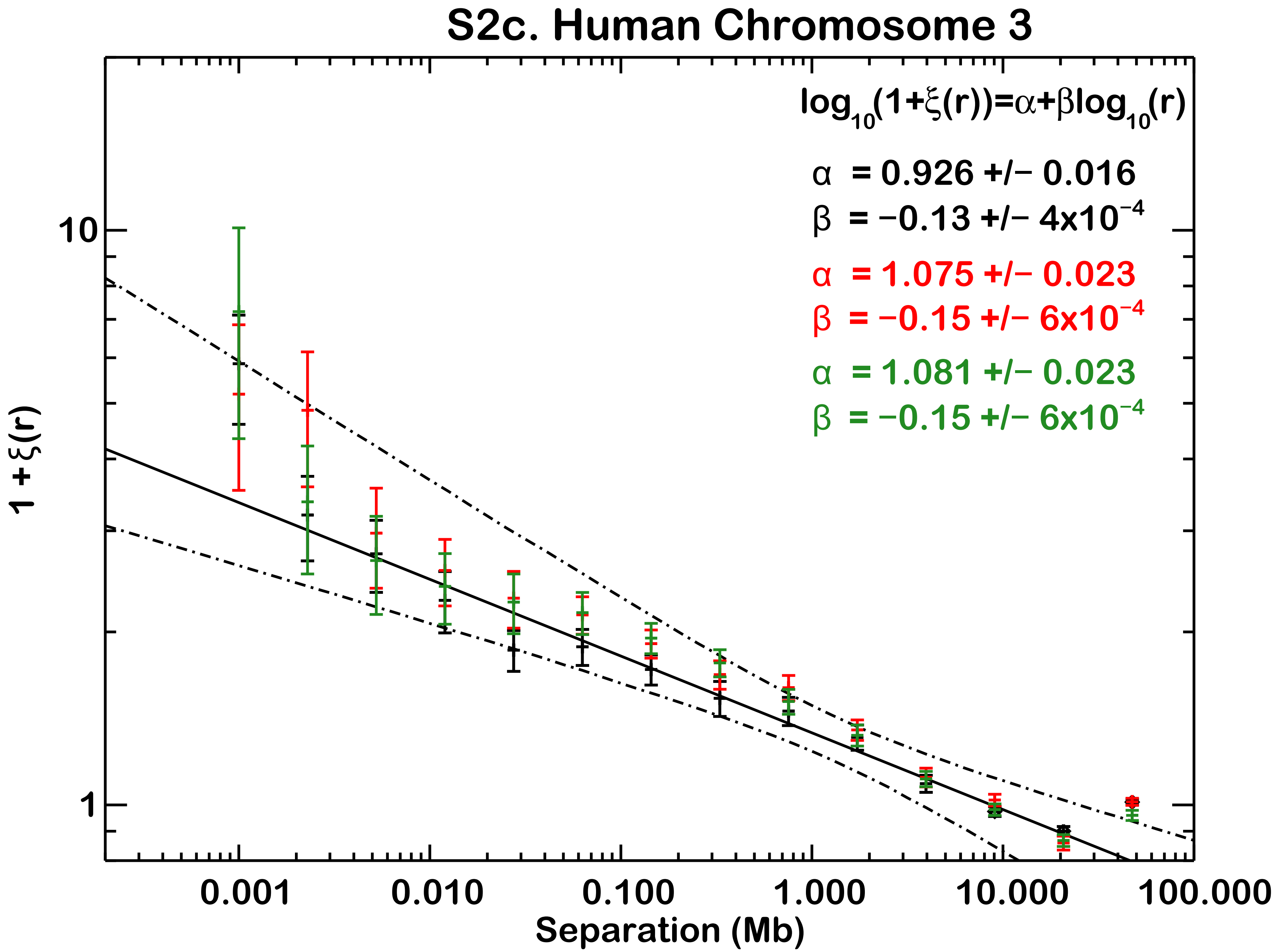

S2c. Human Chromosome 3
log10(1+ξ(r))=α+βlog10(r)
α = 0.926 +/− 0.016
β = −0.13 +/− 4x10−4
α = 1.075 +/− 0.023
β = −0.15 +/− 6x10−4
α = 1.081 +/− 0.023
β = −0.15 +/− 6x10−4
10
1
1 + ξ(r)
0.001
0.010
0.100
1.000
10.000
100.000
Separation (Mb)

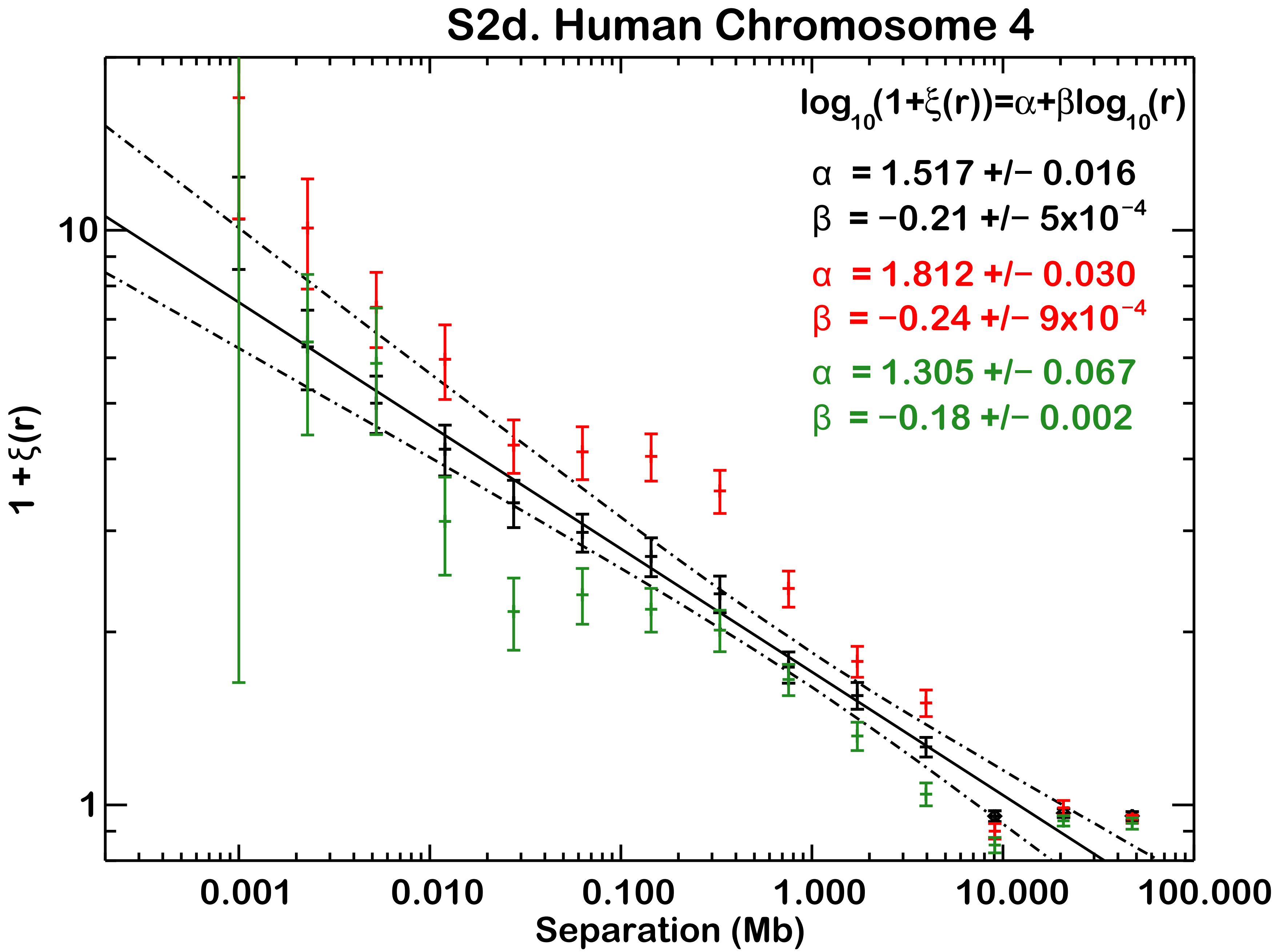
S2d. Human Chromosome 4
log10(1+ξ(r))=α+βlog10(r)
α = 1.517 +/− 0.016
β = −0.21 +/− 5x10−4
α = 1.812 +/− 0.030
β = −0.24 +/− 9x10−4
α = 1.305 +/− 0.067
β = −0.18 +/− 0.002
10
1
1 + ξ(r)
0.001
0.010
0.100
1.000
10.000
100.000
Separation (Mb)

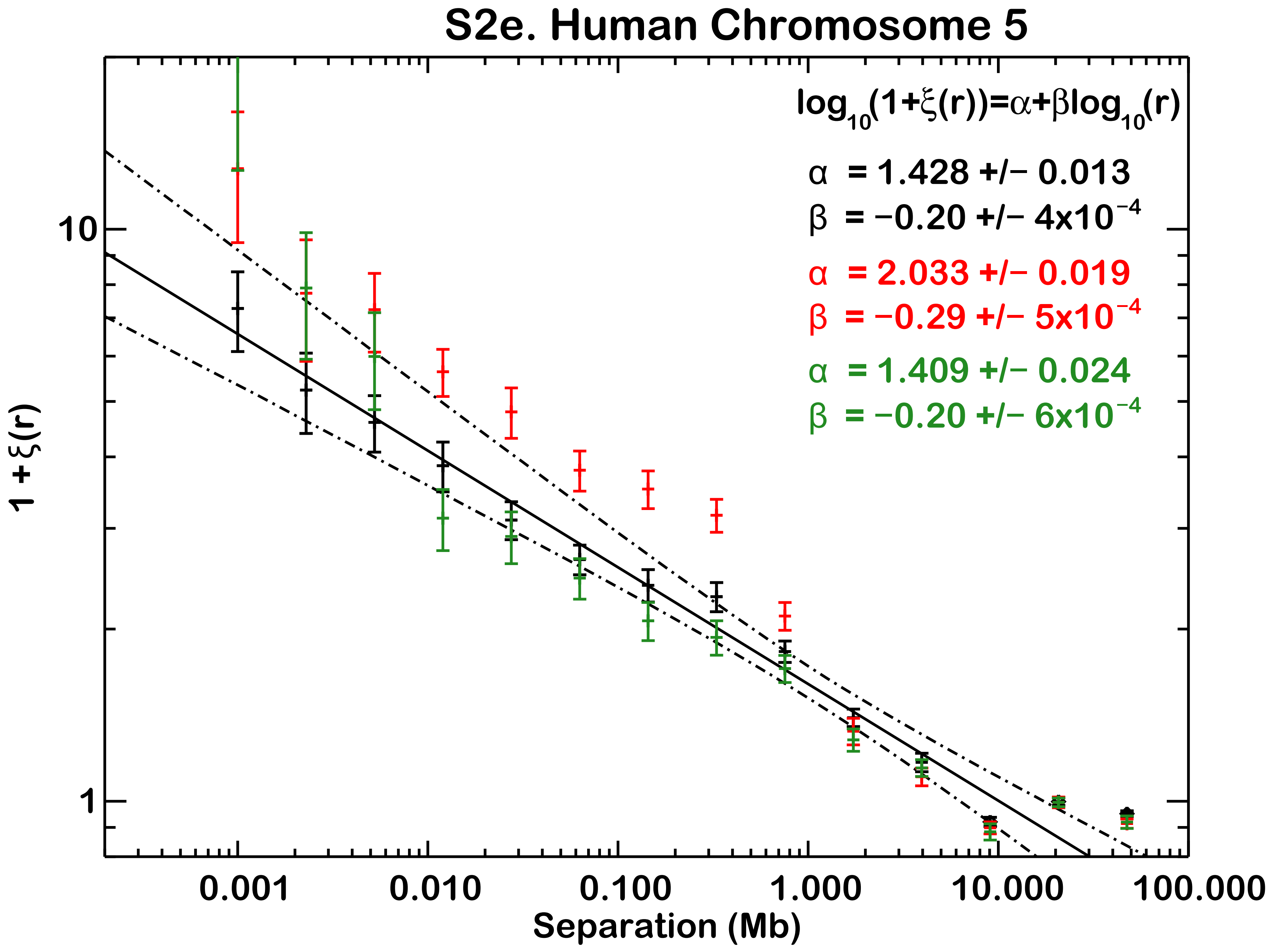

S2e. Human Chromosome 5
$\log_{10}(1+\xi(r))=\alpha+\beta\log_{10}(r)$
α = 1.428 +/− 0.013
β = −0.20 +/− 4x10^{-4}
α = 2.033 +/− 0.019
β = −0.29 +/− 5x10^{-4}
α = 1.409 +/− 0.024
β = −0.20 +/− 6x10^{-4}
10
1
1 + ξ(r)
0.001
0.010
0.100
1.000
10.000
100.000
Separation (Mb)

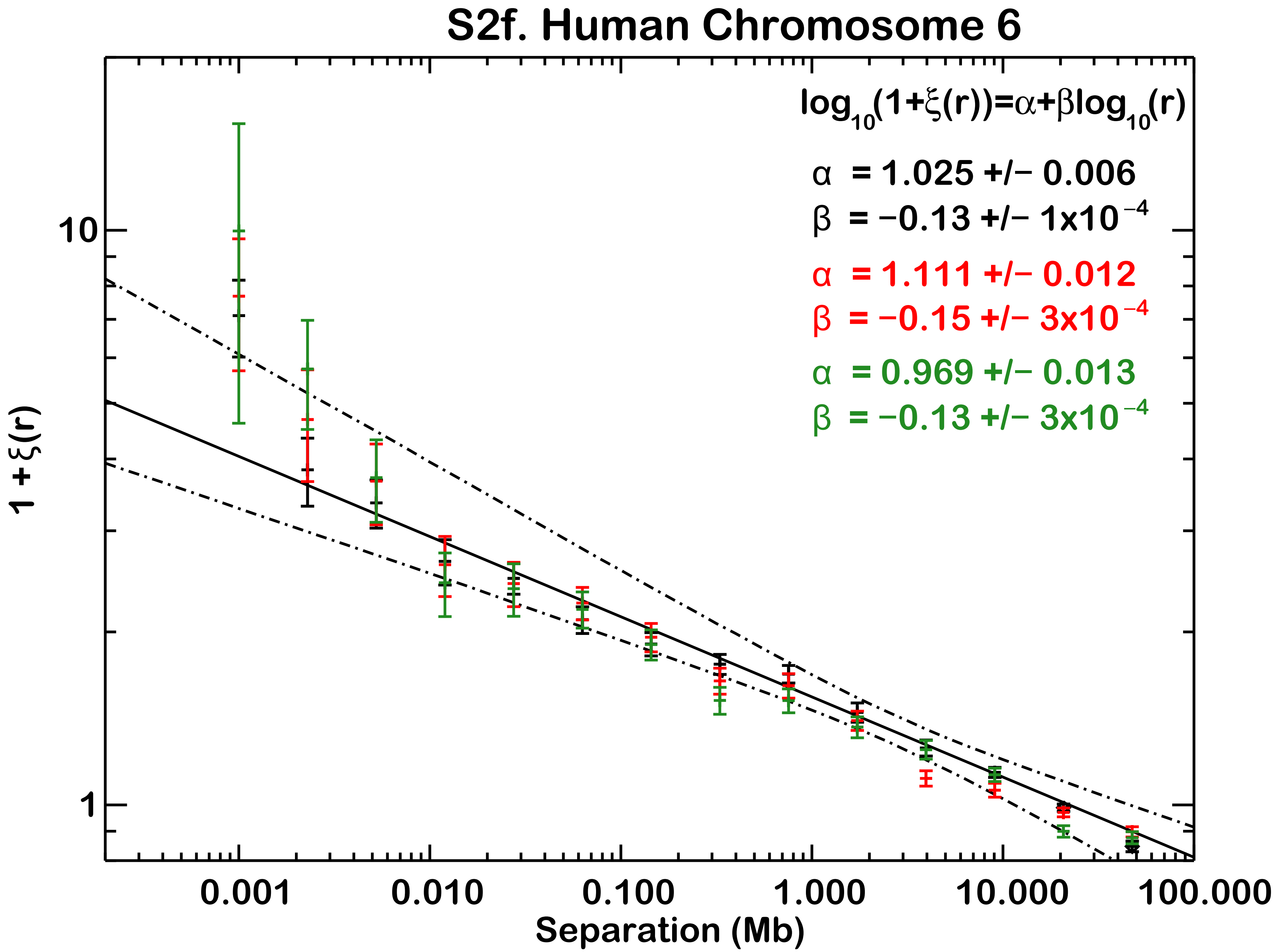
S2f. Human Chromosome 6
$\log_{10}(1+\xi(r))=\alpha+\beta\log_{10}(r)$
α = 1.025 +/− 0.006
β = −0.13 +/− 1×10^{-4}
α = 1.111 +/− 0.012
β = −0.15 +/− 3×10^{-4}
α = 0.969 +/− 0.013
β = −0.13 +/− 3×10^{-4}
10
1
$1+\xi(r)$
0.001
0.010
0.100
1.000
10.000
100.000
Separation (Mb)

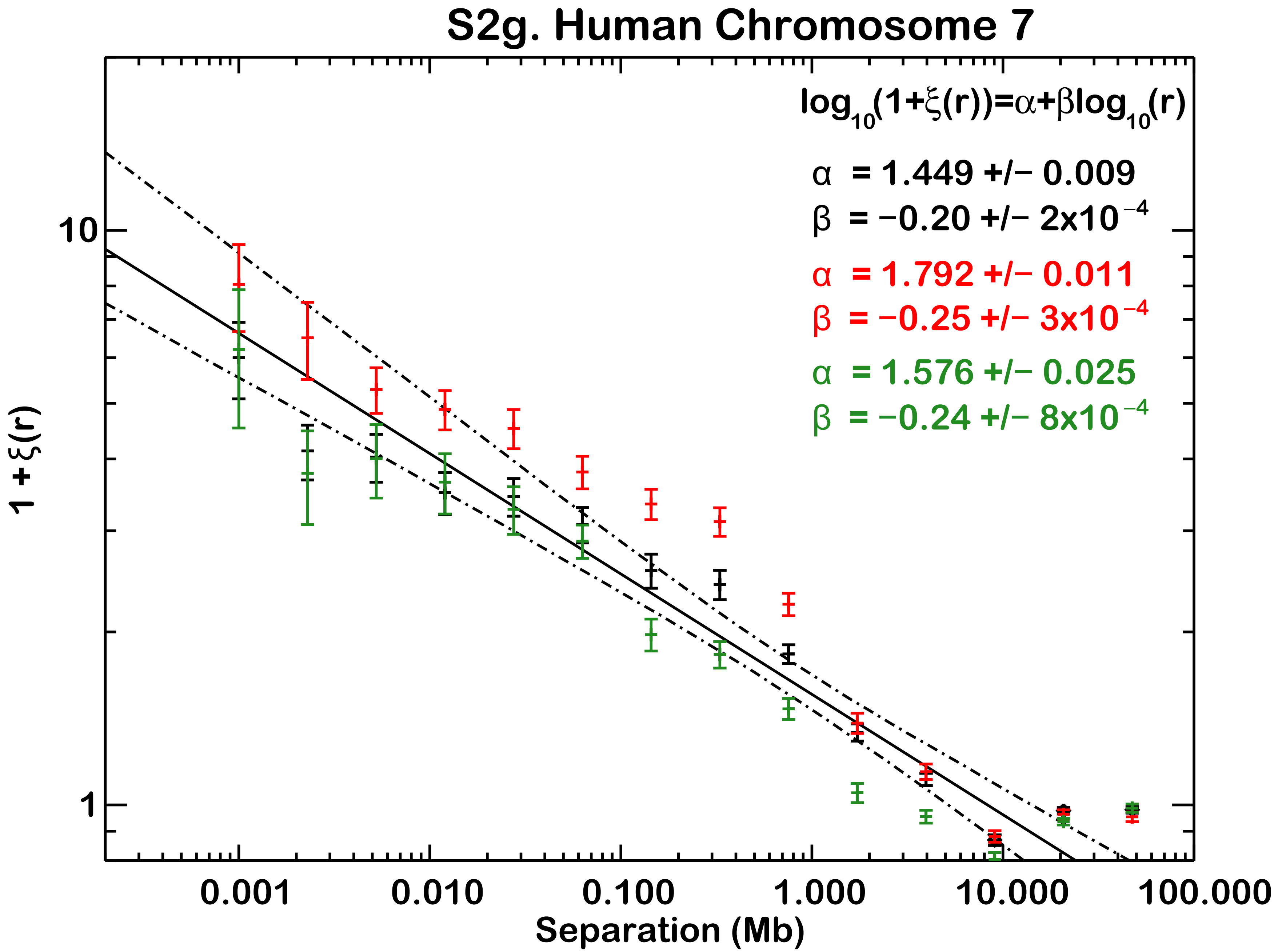

S2g. Human Chromosome 7
log10(1+ξ(r))=α+βlog10(r)
α = 1.449 +/− 0.009
β = −0.20 +/− 2x10−4
α = 1.792 +/− 0.011
β = −0.25 +/− 3x10−4
α = 1.576 +/− 0.025
β = −0.24 +/− 8x10−4
10
1
1 + ξ(r)
0.001
0.010
0.100
1.000
10.000
100.000
Separation (Mb)

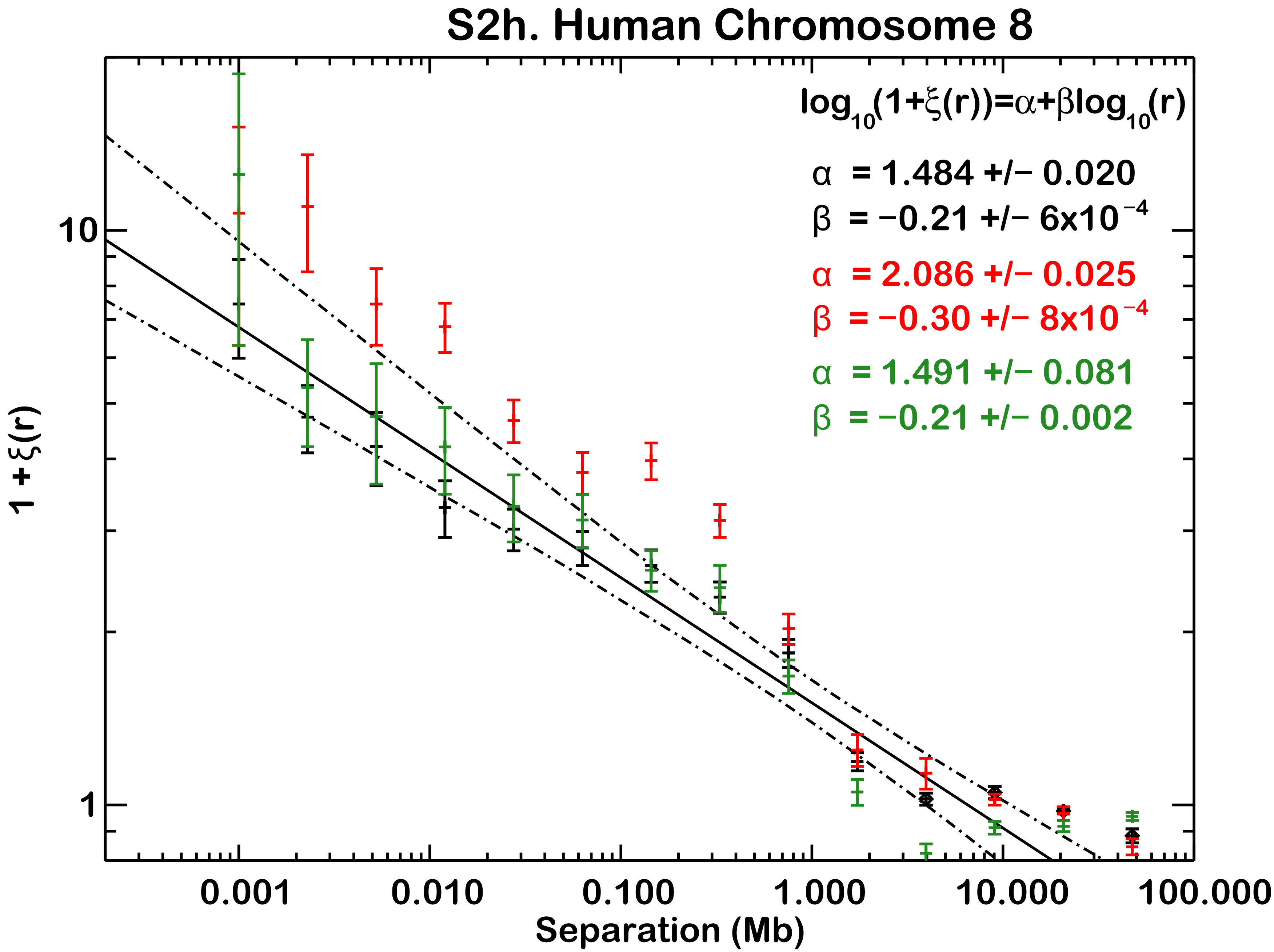

S2h. Human Chromosome 8
log10(1+ξ(r))=α+βlog10(r)
α = 1.484 +/− 0.020
β = −0.21 +/− 6x10−4
α = 2.086 +/− 0.025
β = −0.30 +/− 8x10−4
α = 1.491 +/− 0.081
β = −0.21 +/− 0.002
10
1
1 + ξ(r)
0.001
0.010
0.100
1.000
10.000
100.000
Separation (Mb)

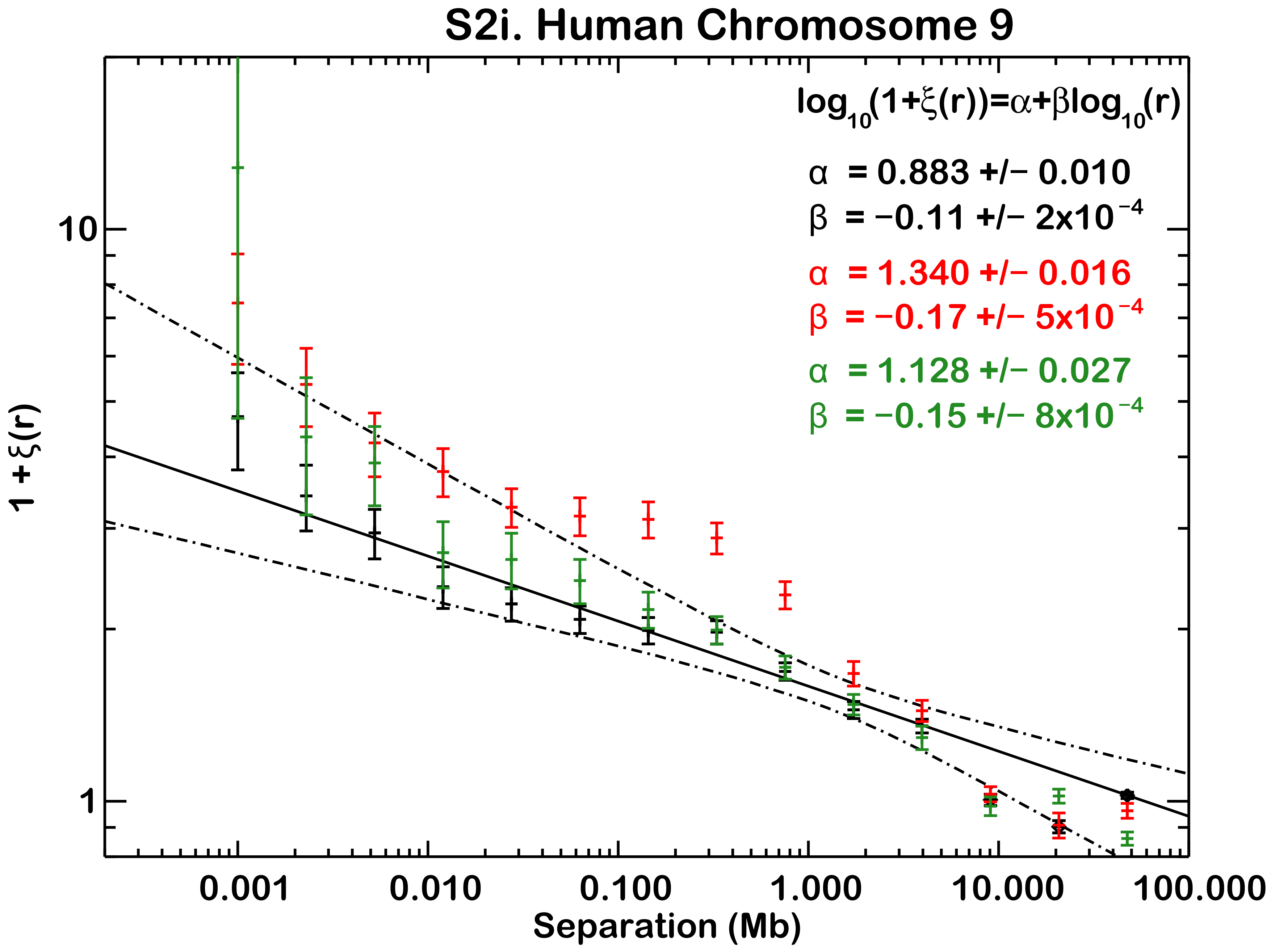

S2i. Human Chromosome 9
$\log_{10}(1+\xi(r))=\alpha+\beta\log_{10}(r)$
α = 0.883 +/− 0.010
β = −0.11 +/− 2×10^{-4}
α = 1.340 +/− 0.016
β = −0.17 +/− 5×10^{-4}
α = 1.128 +/− 0.027
β = −0.15 +/− 8×10^{-4}
10
1
$1+\xi(r)$
0.001
0.010
0.100
1.000
10.000
100.000
Separation (Mb)

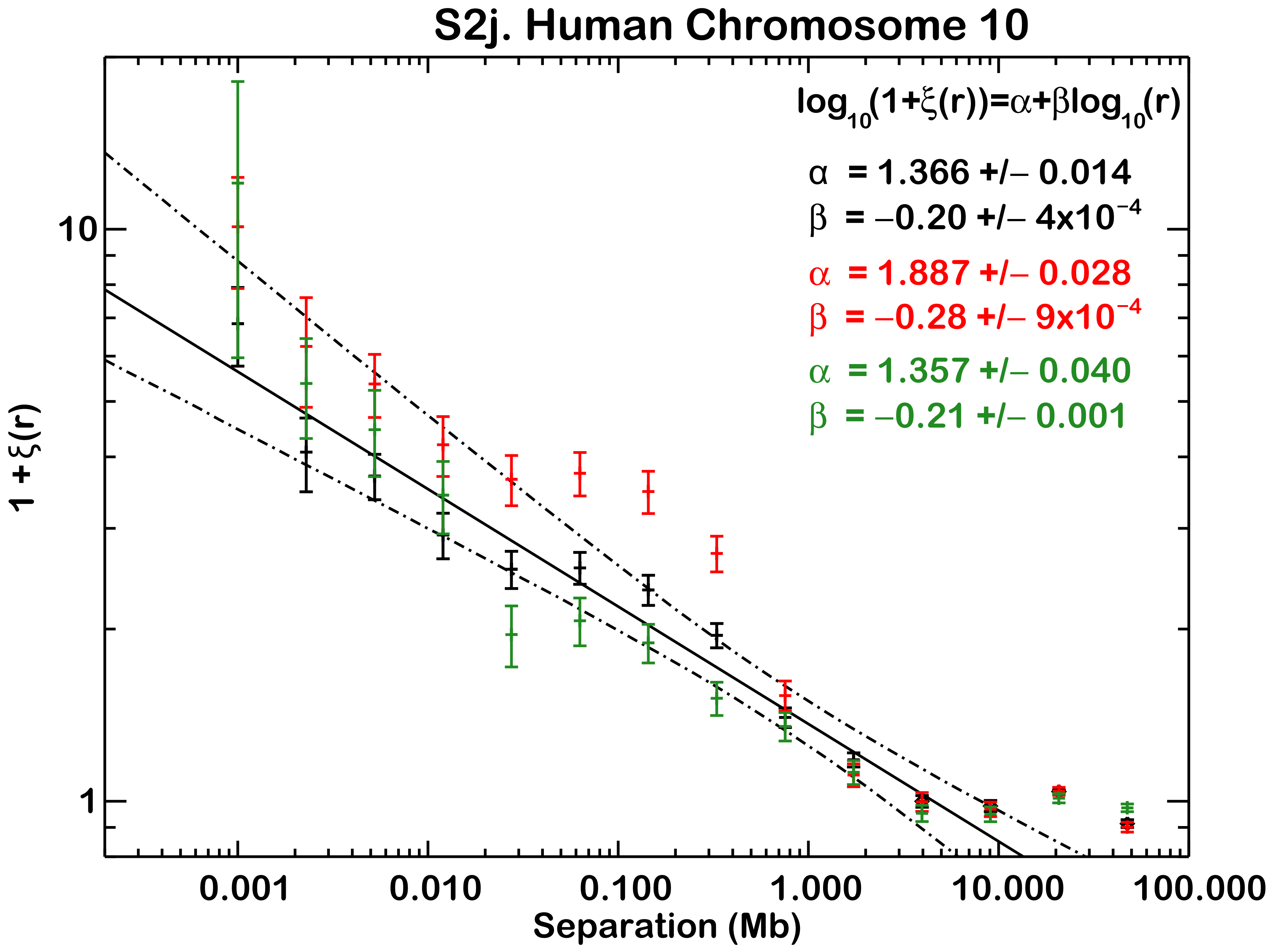
S2j. Human Chromosome 10
log10(1+ξ(r))=α+βlog10(r)
α = 1.366 +/– 0.014
β = –0.20 +/– 4x10^-4
α = 1.887 +/– 0.028
β = –0.28 +/– 9x10^-4
α = 1.357 +/– 0.040
β = –0.21 +/– 0.001
10
1
1 + ξ(r)
0.001
0.010
0.100
1.000
10.000
100.000
Separation (Mb)

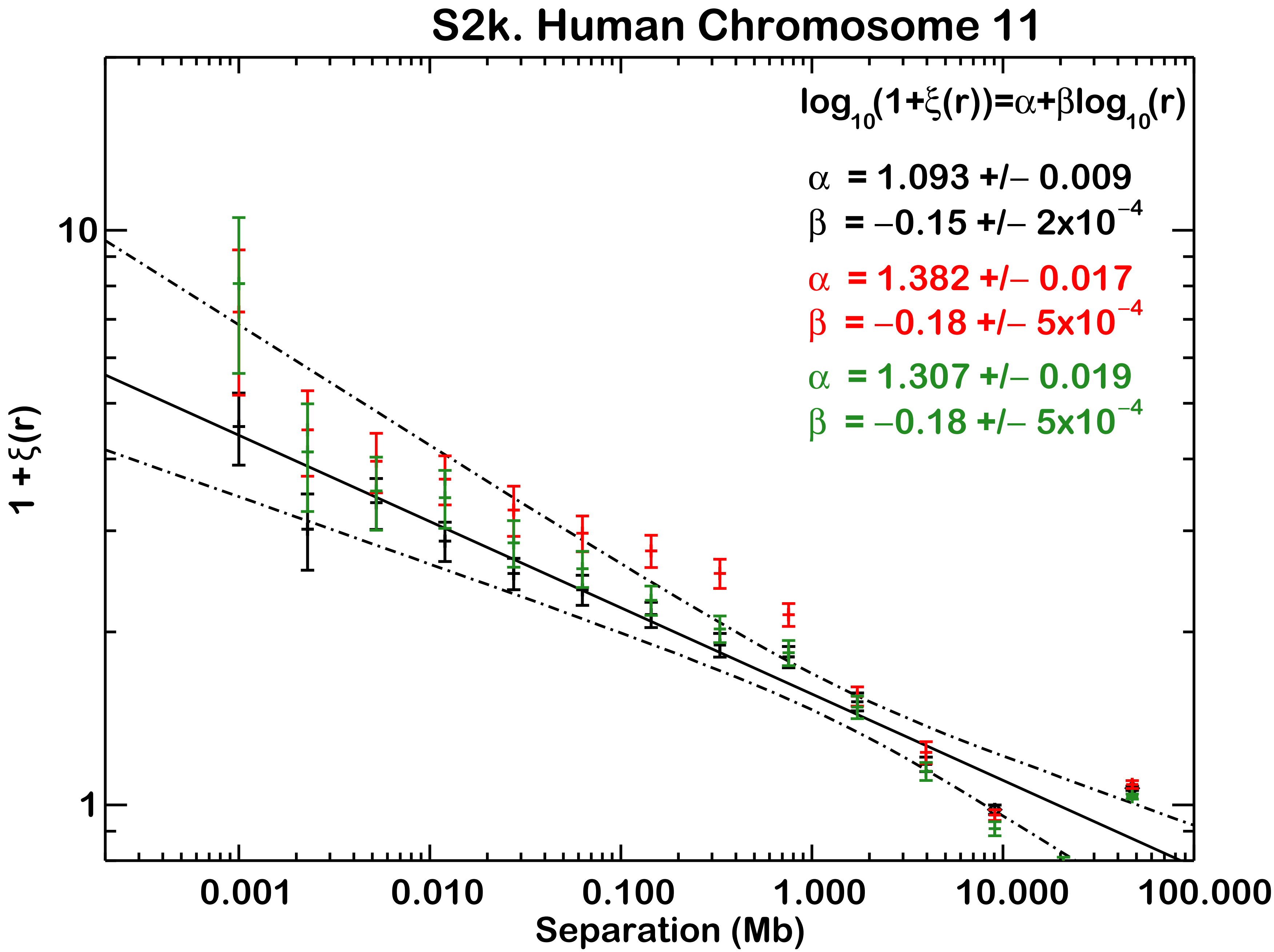

S2k. Human Chromosome 11
log10(1+ξ(r))=α+βlog10(r)
α = 1.093 +/– 0.009
β = –0.15 +/– 2x10–4
α = 1.382 +/– 0.017
β = –0.18 +/– 5x10–4
α = 1.307 +/– 0.019
β = –0.18 +/– 5x10–4
1 + ξ(r)
10
1
0.001
0.010
0.100
1.000
10.000
100.000
Separation (Mb)

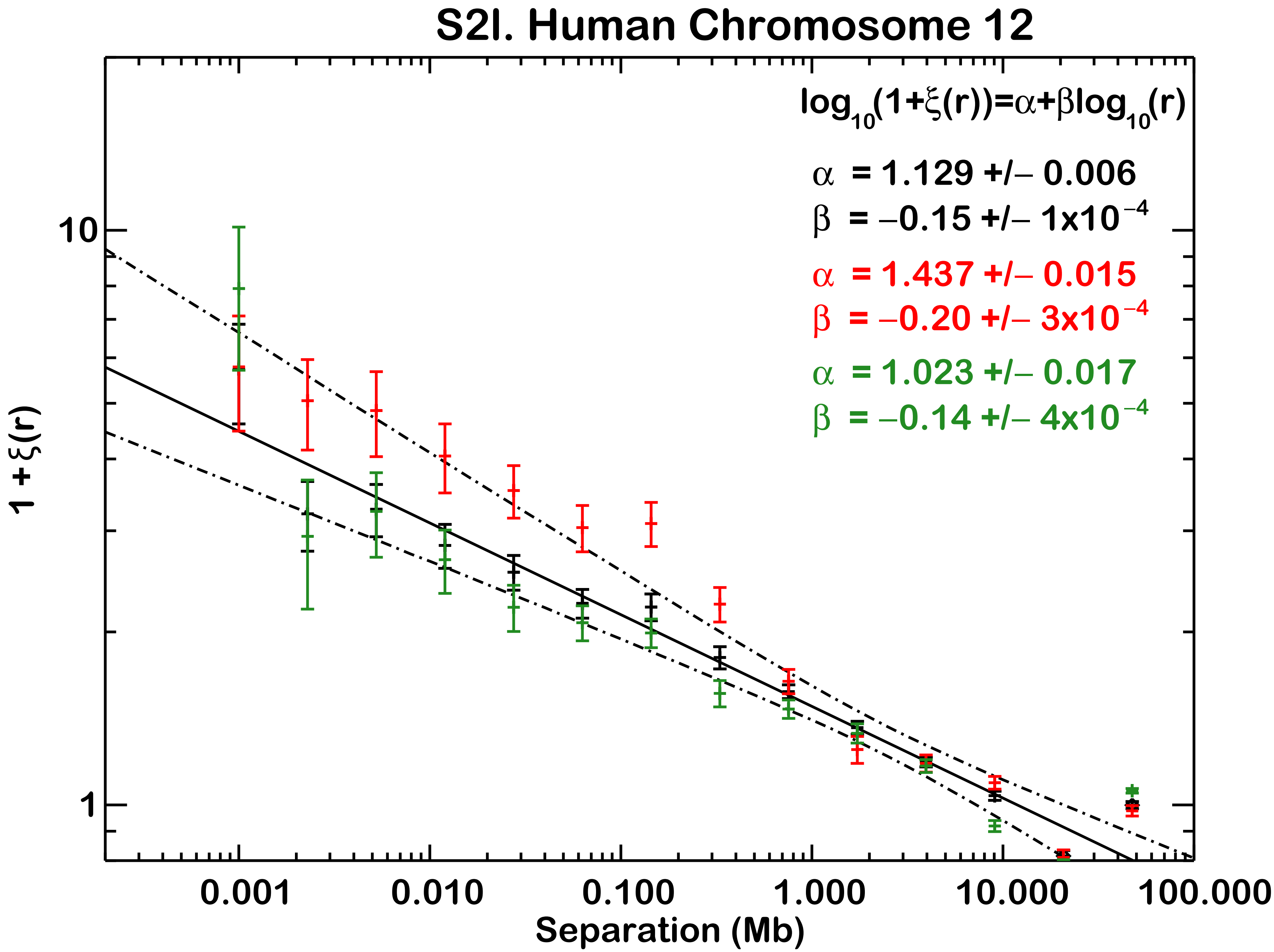

S2I. Human Chromosome 12
$\log_{10}(1+\xi(r))=\alpha+\beta\log_{10}(r)$
α = 1.129 +/– 0.006
β = –0.15 +/– 1×10^{-4}
α = 1.437 +/– 0.015
β = –0.20 +/– 3×10^{-4}
α = 1.023 +/– 0.017
β = –0.14 +/– 4×10^{-4}
10
1
1 + ξ(r)
0.001
0.010
0.100
1.000
10.000
100.000
Separation (Mb)

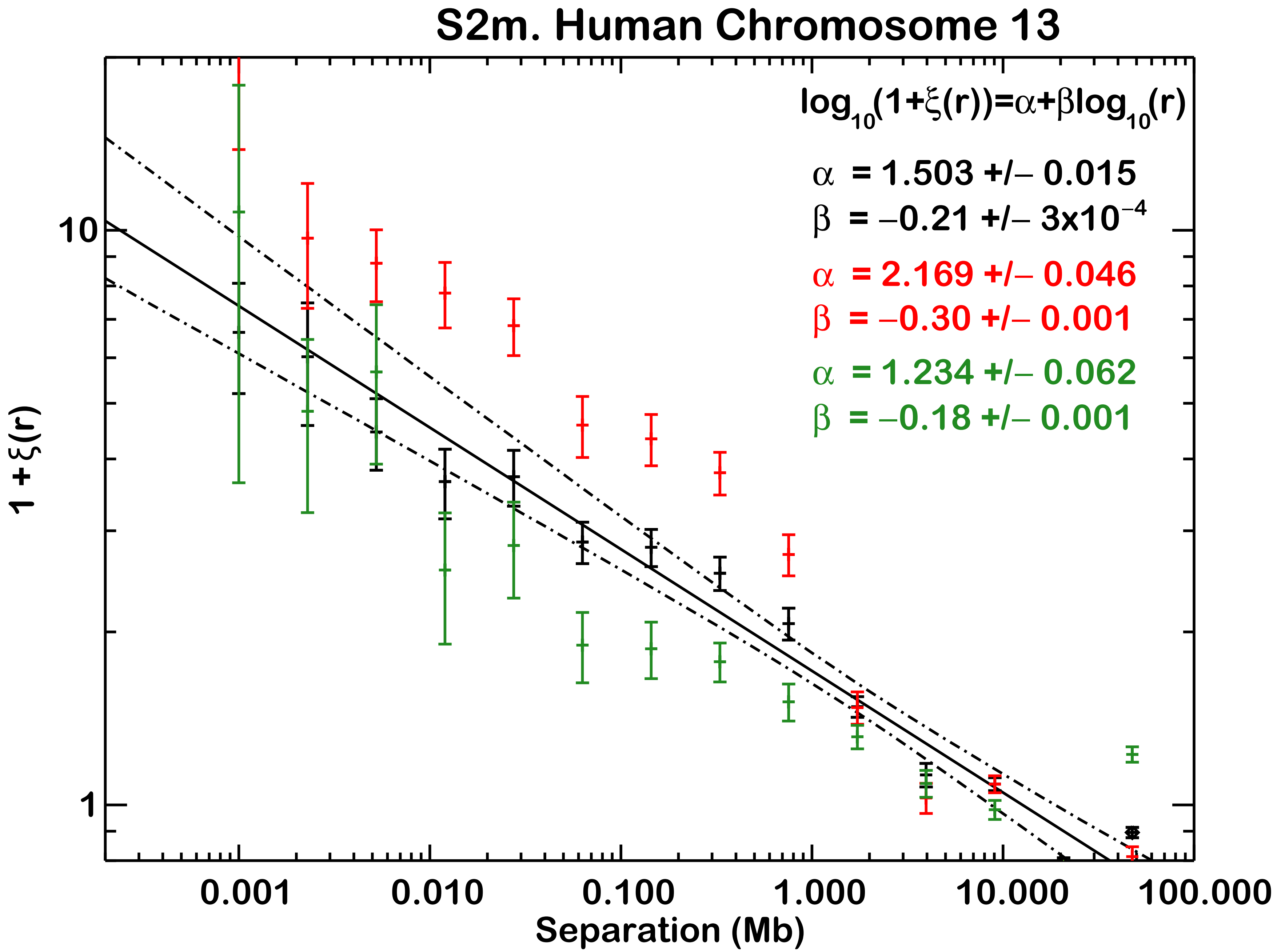

S2m. Human Chromosome 13
log10(1+ξ(r))=α+βlog10(r)
α = 1.503 +/– 0.015
β = –0.21 +/– 3x10–4
α = 2.169 +/– 0.046
β = –0.30 +/– 0.001
α = 1.234 +/– 0.062
β = –0.18 +/– 0.001
10
1
1 +ξ(r)
0.001
0.010
0.100
1.000
10.000
100.000
Separation (Mb)

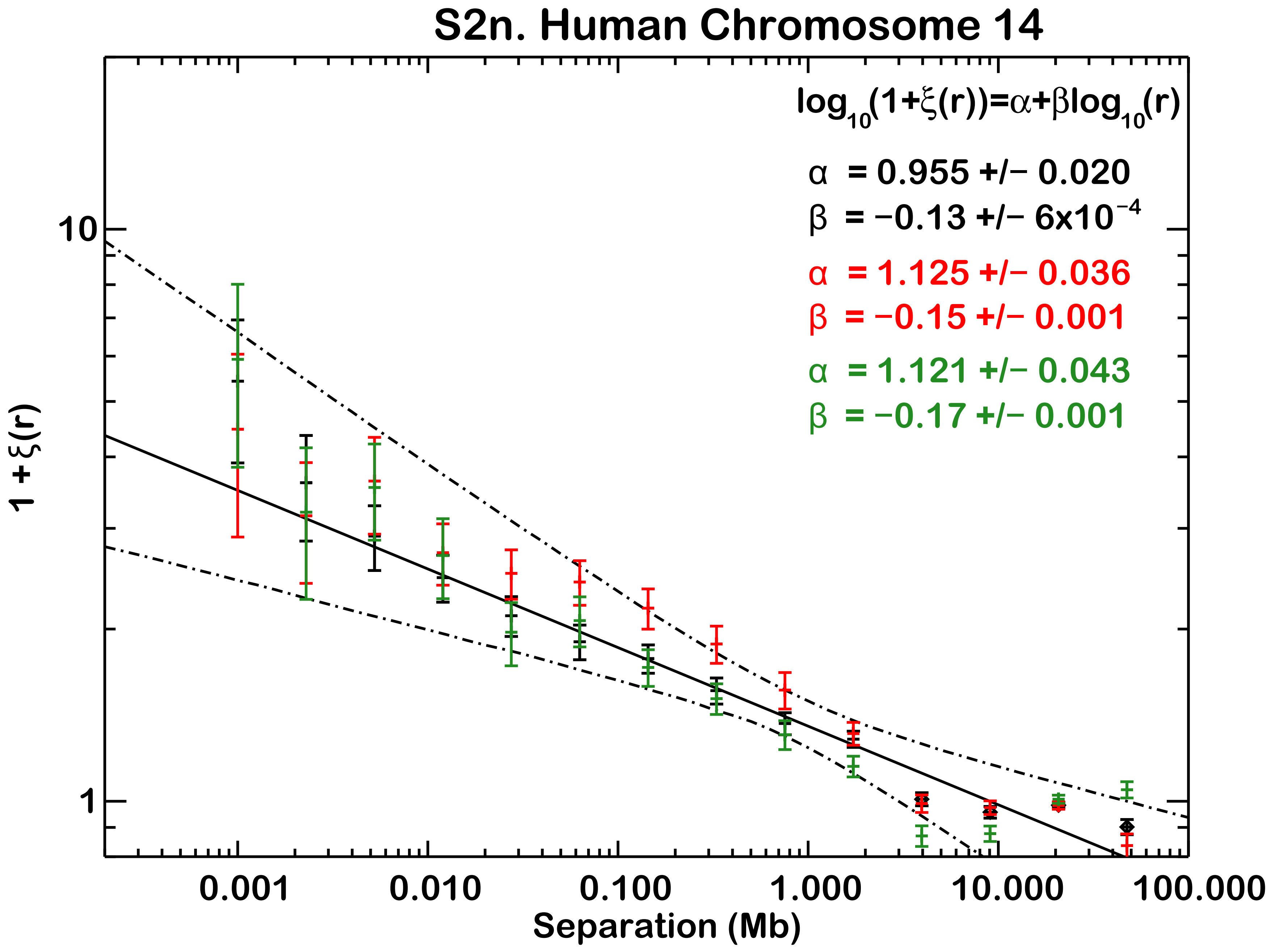
S2n. Human Chromosome 14
log10(1+ξ(r))=α+βlog10(r)
α = 0.955 +/− 0.020
β = −0.13 +/− 6x10−4
α = 1.125 +/− 0.036
β = −0.15 +/− 0.001
α = 1.121 +/− 0.043
β = −0.17 +/− 0.001
10
1
1 + ξ(r)
0.001
0.010
0.100
1.000
10.000
100.000
Separation (Mb)

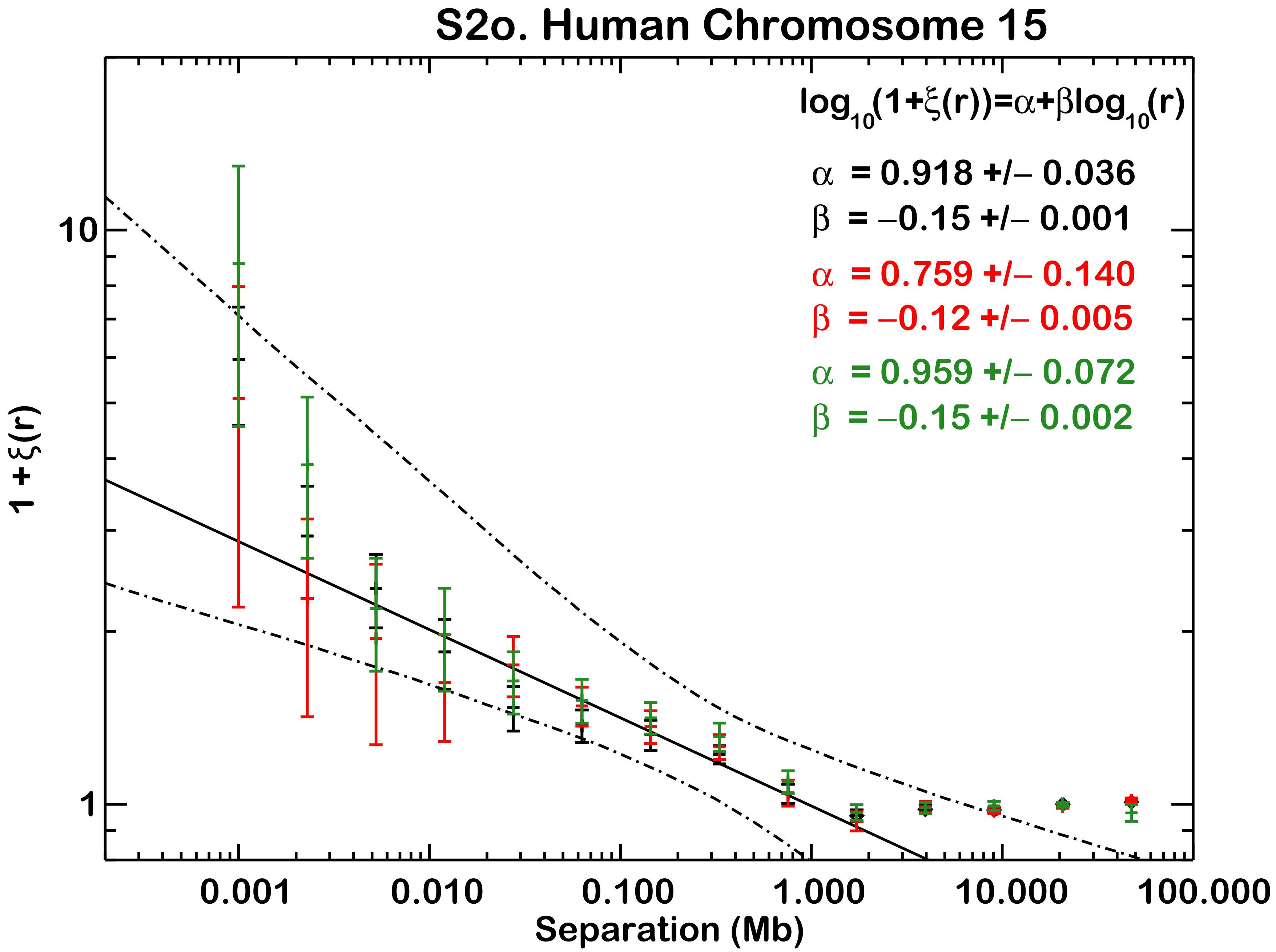

S2o. Human Chromosome 15
log10(1+ξ(r))=α+βlog10(r)
α = 0.918 +/– 0.036
β = –0.15 +/– 0.001
α = 0.759 +/– 0.140
β = –0.12 +/– 0.005
α = 0.959 +/– 0.072
β = –0.15 +/– 0.002
1 + ξ(r)
10
1
0.001
0.010
0.100
1.000
10.000
100.000
Separation (Mb)

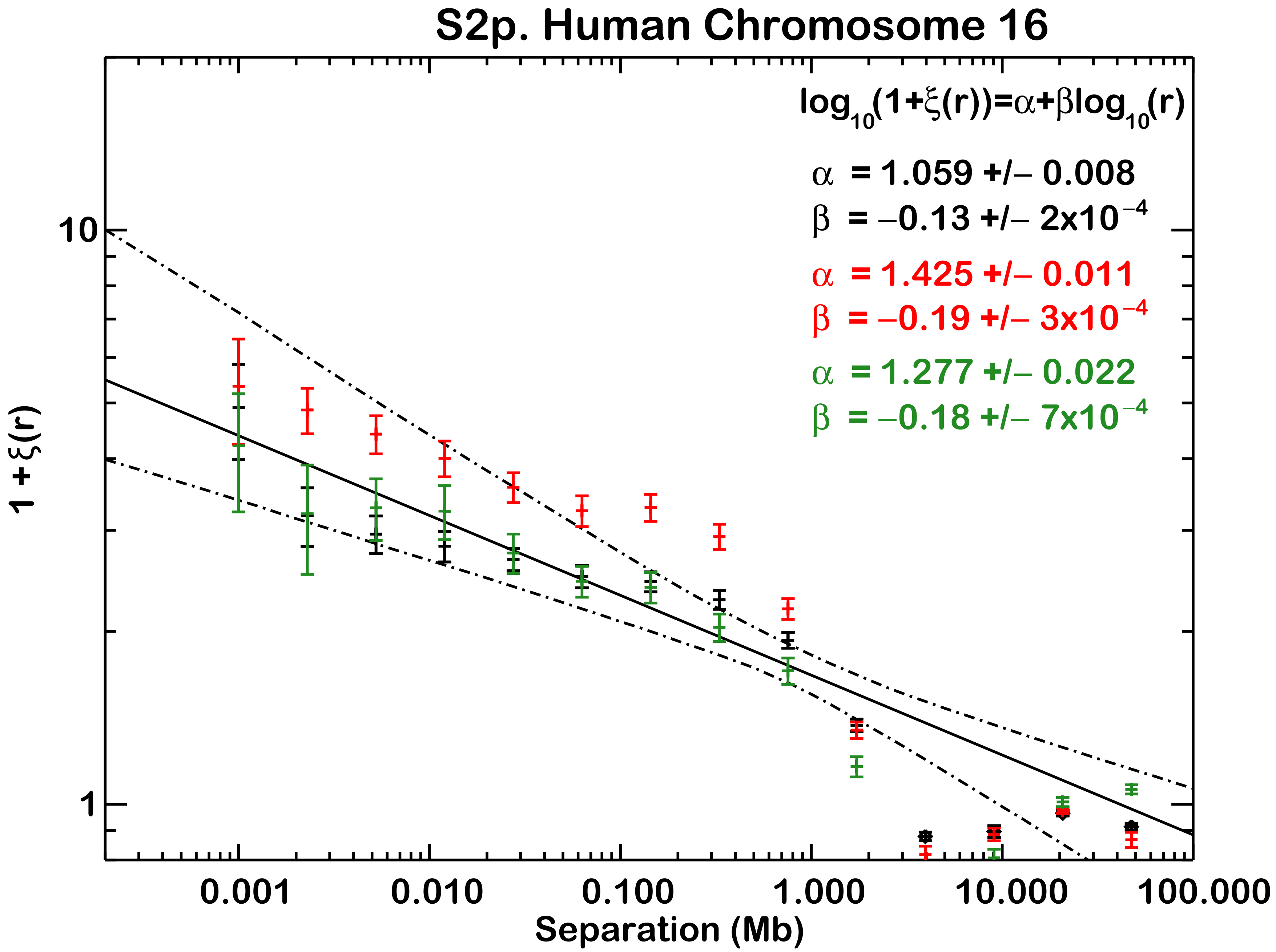

S2p. Human Chromosome 16
log10(1+ξ(r))=α+βlog10(r)
α = 1.059 +/– 0.008
β = –0.13 +/– 2x10^–4
α = 1.425 +/– 0.011
β = –0.19 +/– 3x10^–4
α = 1.277 +/– 0.022
β = –0.18 +/– 7x10^–4
10
1
1 + ξ(r)
0.001
0.010
0.100
1.000
10.000
100.000
Separation (Mb)

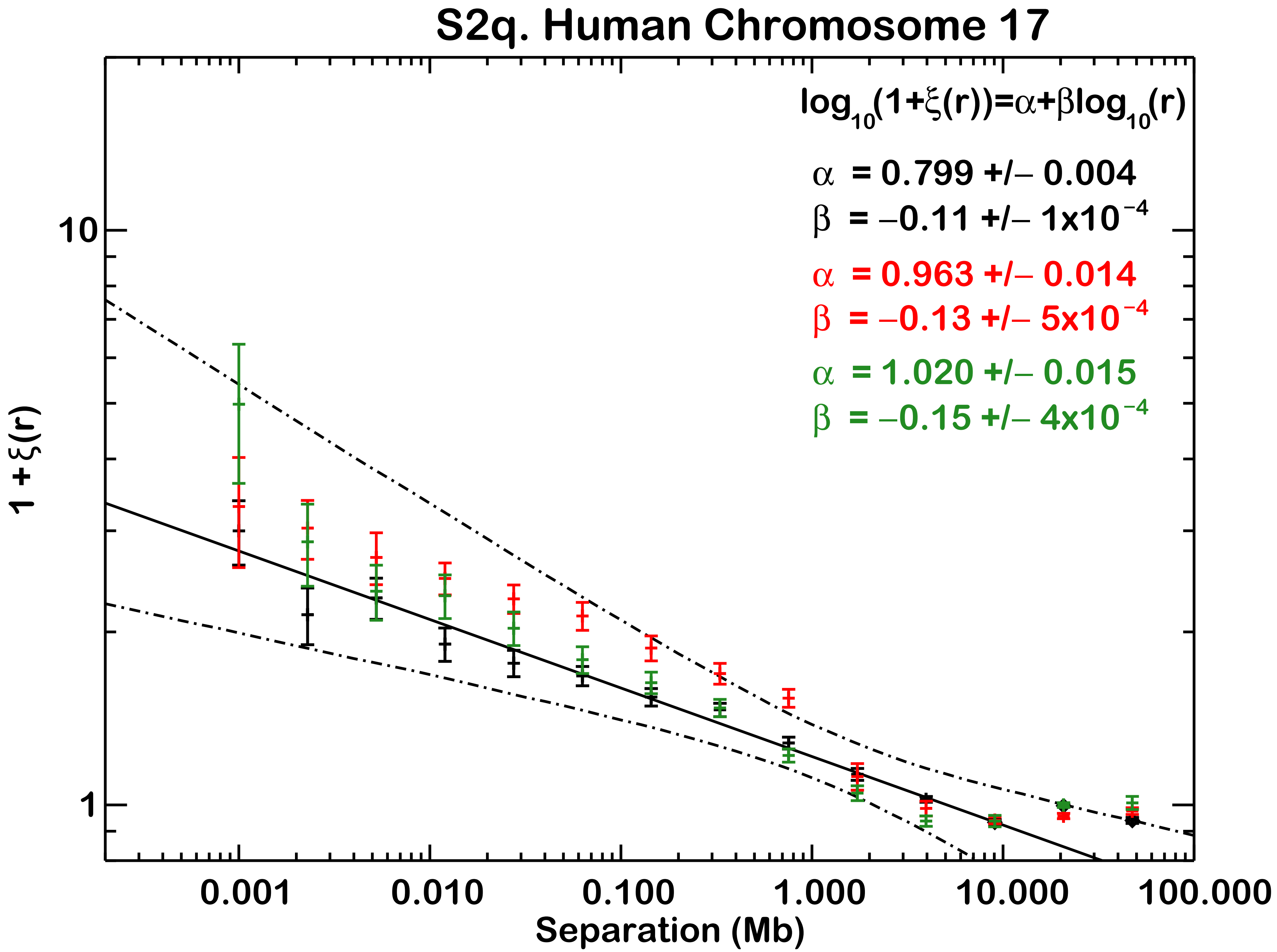

S2q. Human Chromosome 17
log10(1+ξ(r))=α+βlog10(r)
α = 0.799 +/– 0.004
β = –0.11 +/– 1x10^–4
α = 0.963 +/– 0.014
β = –0.13 +/– 5x10^–4
α = 1.020 +/– 0.015
β = –0.15 +/– 4x10^–4
10
1
1 + ξ(r)
0.001
0.010
0.100
1.000
10.000
100.000
Separation (Mb)

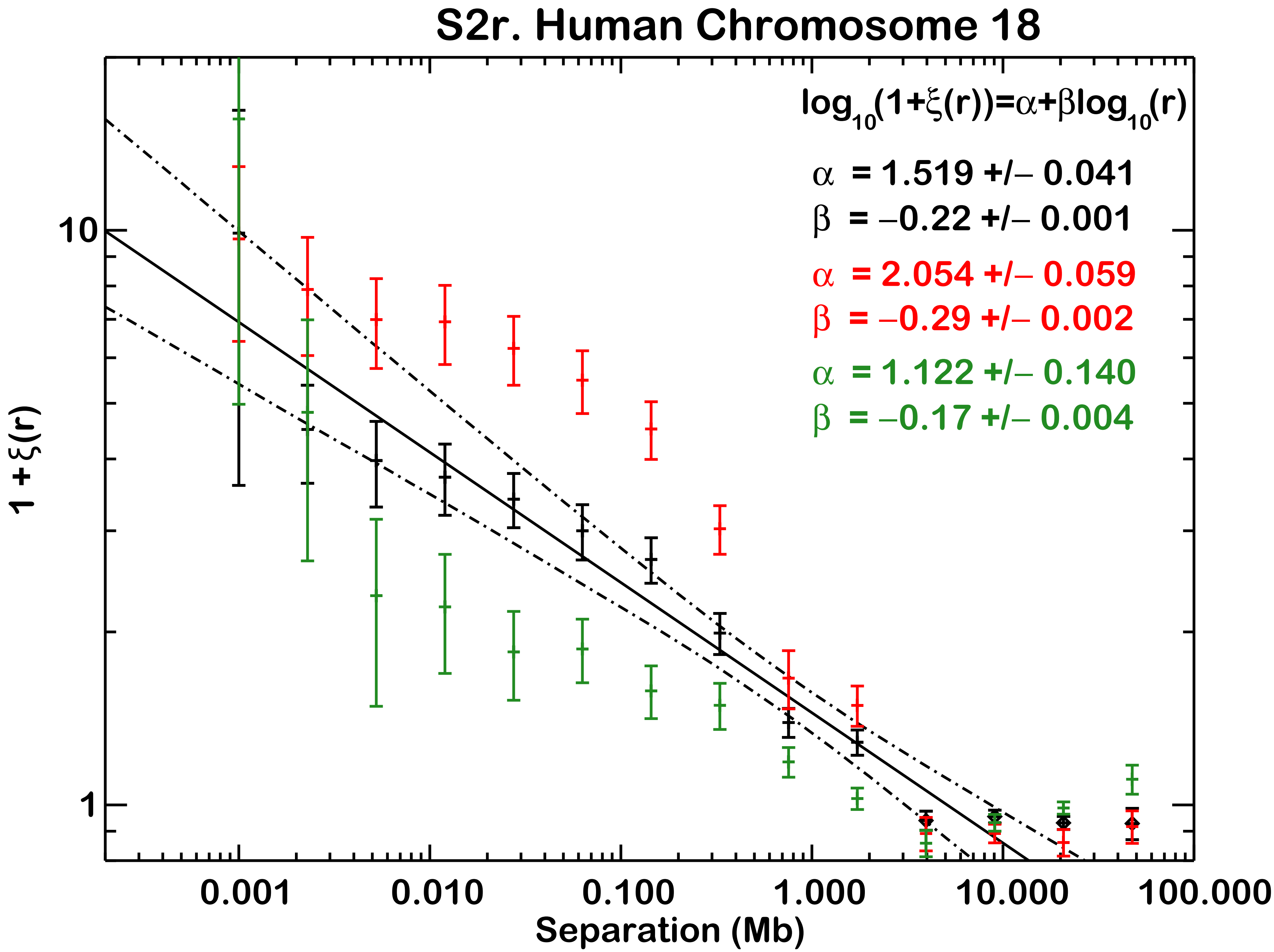

S2r. Human Chromosome 18
log10(1+ξ(r))=α+βlog10(r)
α = 1.519 +/– 0.041
β = –0.22 +/– 0.001
α = 2.054 +/– 0.059
β = –0.29 +/– 0.002
α = 1.122 +/– 0.140
β = –0.17 +/– 0.004
10
1
1 +ξ(r)
0.001
0.010
0.100
1.000
10.000
100.000
Separation (Mb)

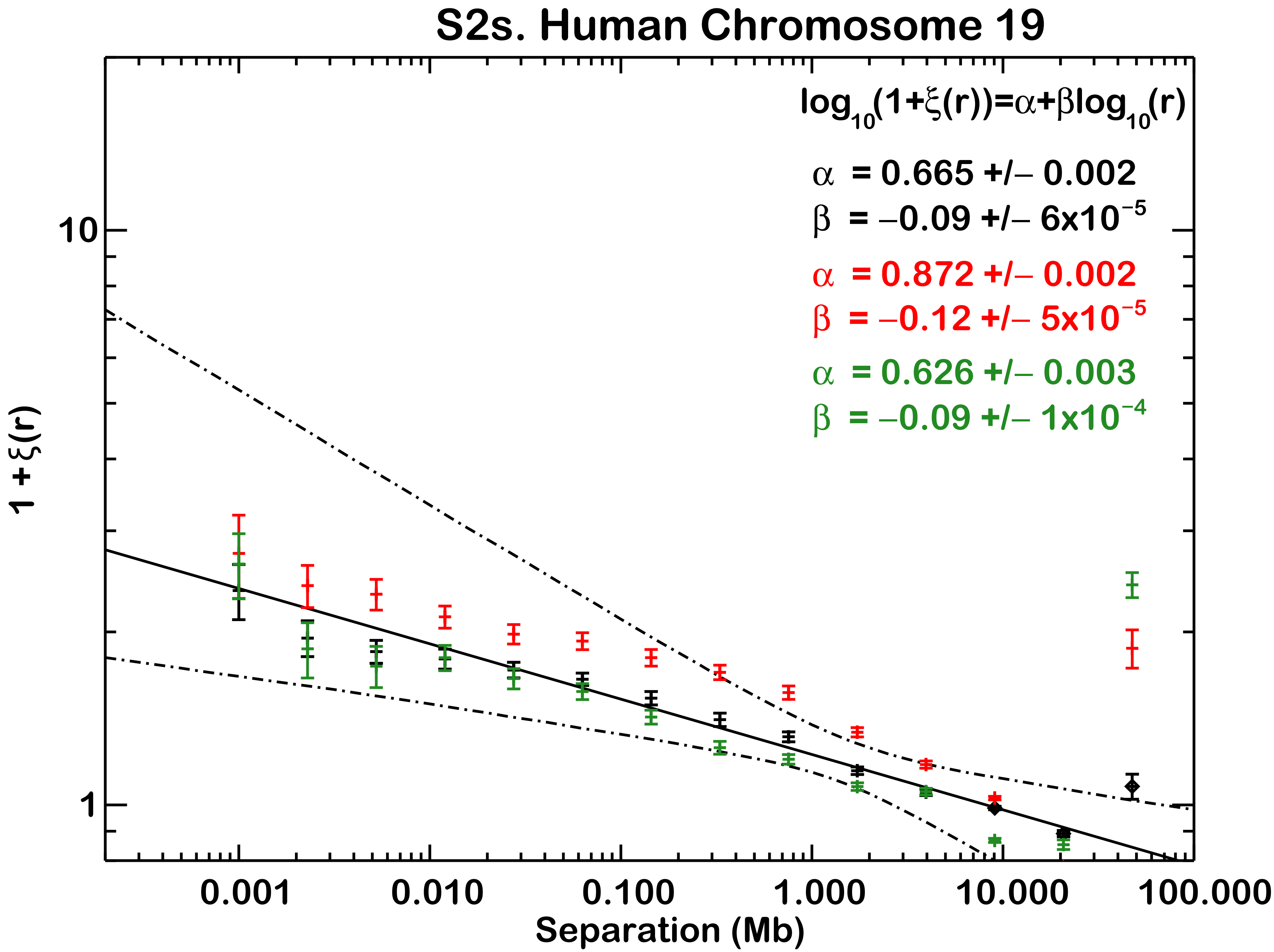

S2s. Human Chromosome 19
log10(1+ξ(r))=α+βlog10(r)
α = 0.665 +/– 0.002
β = –0.09 +/– 6x10–5
α = 0.872 +/– 0.002
β = –0.12 +/– 5x10–5
α = 0.626 +/– 0.003
β = –0.09 +/– 1x10–4
10
1
1 + ξ(r)
0.001
0.010
0.100
1.000
10.000
100.000
Separation (Mb)

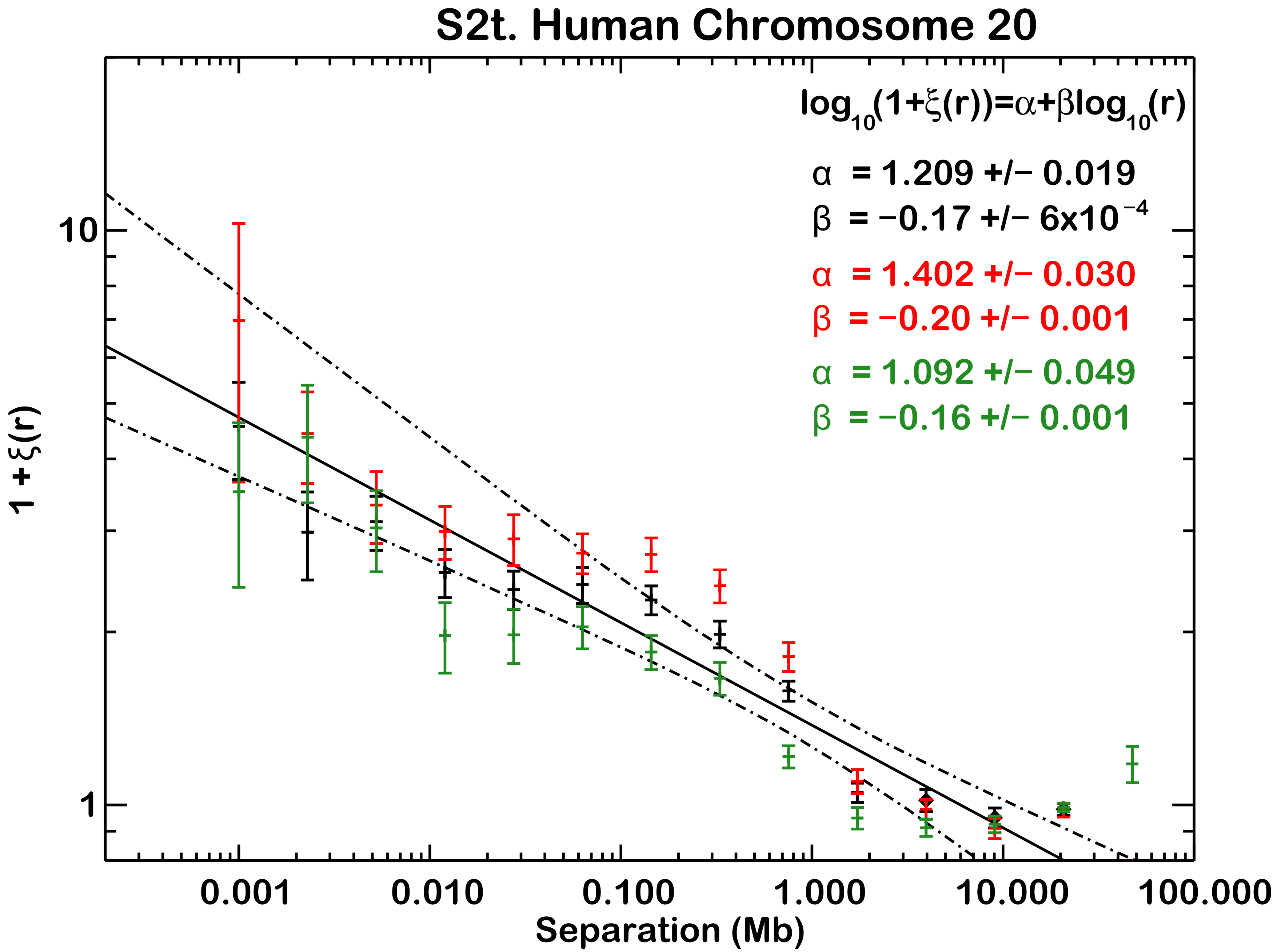

S2t. Human Chromosome 20
log10(1+ξ(r))=α+βlog10(r)
α = 1.209 +/− 0.019
β = −0.17 +/− 6x10−4
α = 1.402 +/− 0.030
β = −0.20 +/− 0.001
α = 1.092 +/− 0.049
β = −0.16 +/− 0.001
10
1
1 +ξ(r)
0.001
0.010
0.100
1.000
10.000
100.000
Separation (Mb)

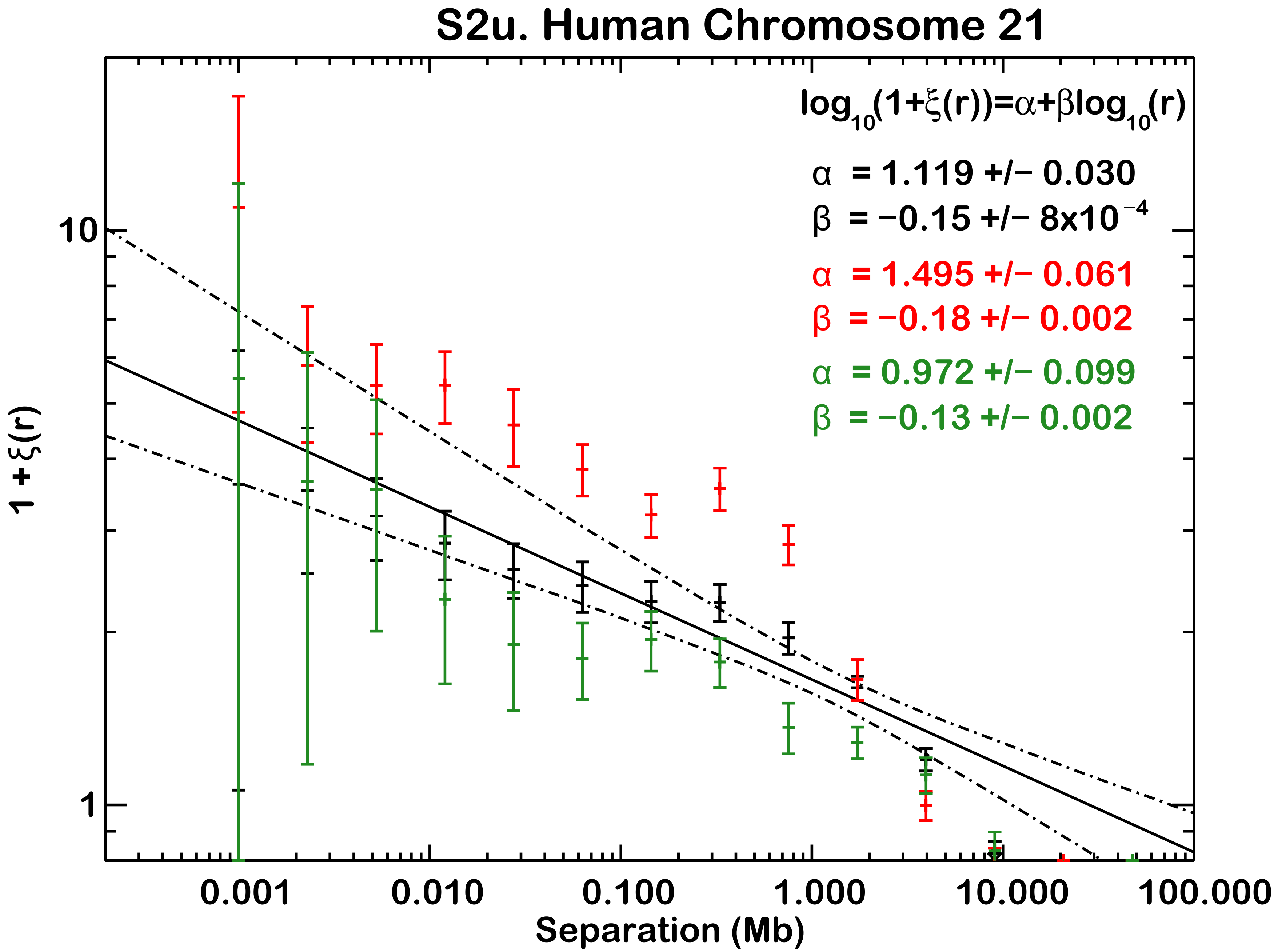

S2u. Human Chromosome 21
$\log_{10}(1+\xi(r))=\alpha+\beta\log_{10}(r)$
α = 1.119 +/− 0.030
β = −0.15 +/− 8×10^{-4}
α = 1.495 +/− 0.061
β = −0.18 +/− 0.002
α = 0.972 +/− 0.099
β = −0.13 +/− 0.002
10
1
$1+\xi(r)$
0.001
0.010
0.100
1.000
10.000
100.000
Separation (Mb)

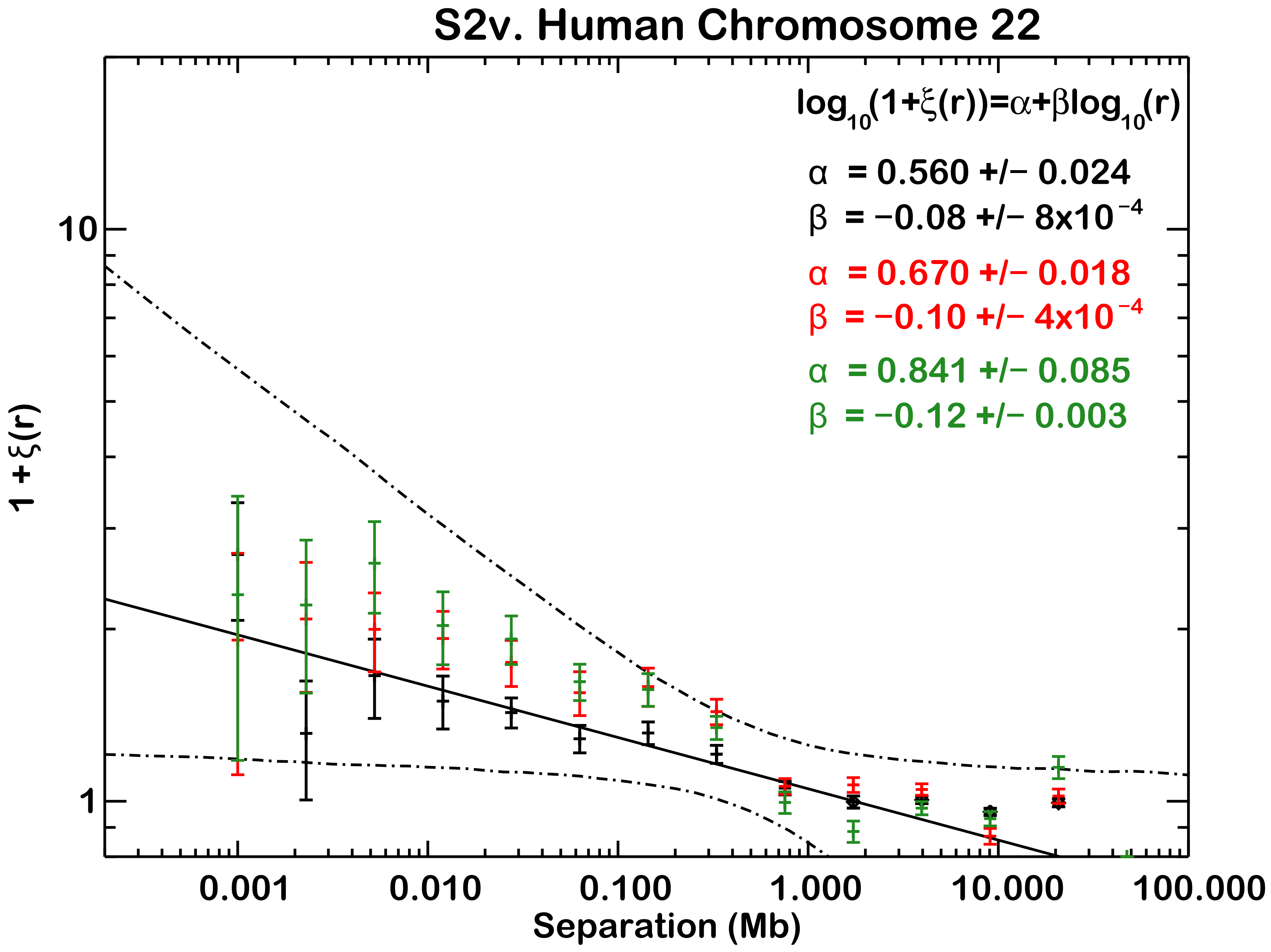

S2v. Human Chromosome 22
$\log_{10}(1+\xi(r))=\alpha+\beta\log_{10}(r)$
α = 0.560 +/− 0.024
β = −0.08 +/− 8×10^{-4}
α = 0.670 +/− 0.018
β = −0.10 +/− 4×10^{-4}
α = 0.841 +/− 0.085
β = −0.12 +/− 0.003
10
1
$1+\xi(r)$
0.001
0.010
0.100
1.000
10.000
100.000
Separation (Mb)

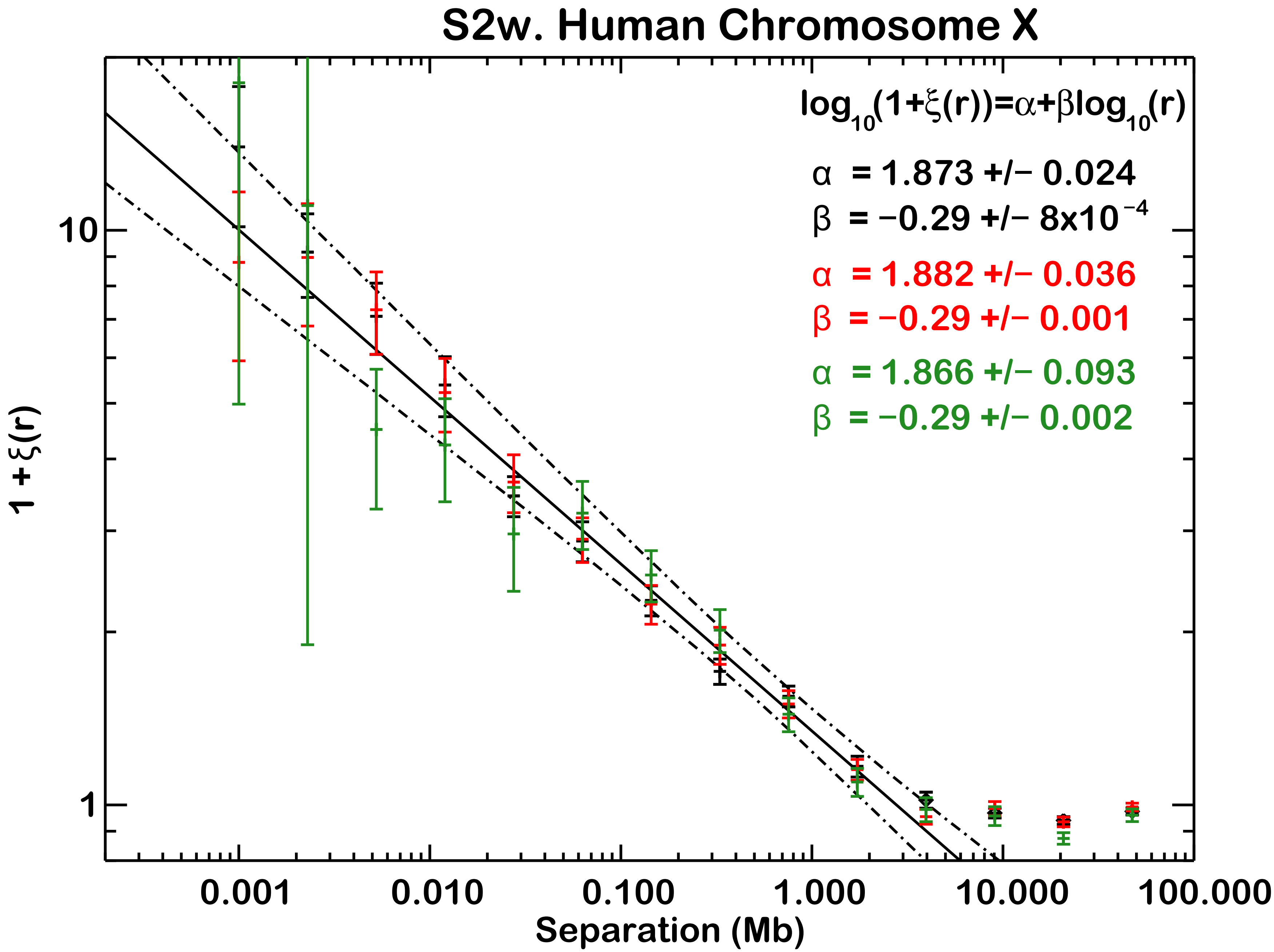

S2w. Human Chromosome X
$\log_{10}(1+\xi(r))=\alpha+\beta\log_{10}(r)$
α = 1.873 +/− 0.024
β = −0.29 +/− 8x10^{-4}
α = 1.882 +/− 0.036
β = −0.29 +/− 0.001
α = 1.866 +/− 0.093
β = −0.29 +/− 0.002
1 + ξ(r)
10
1
0.001
0.010
0.100
1.000
10.000
100.000
Separation (Mb)

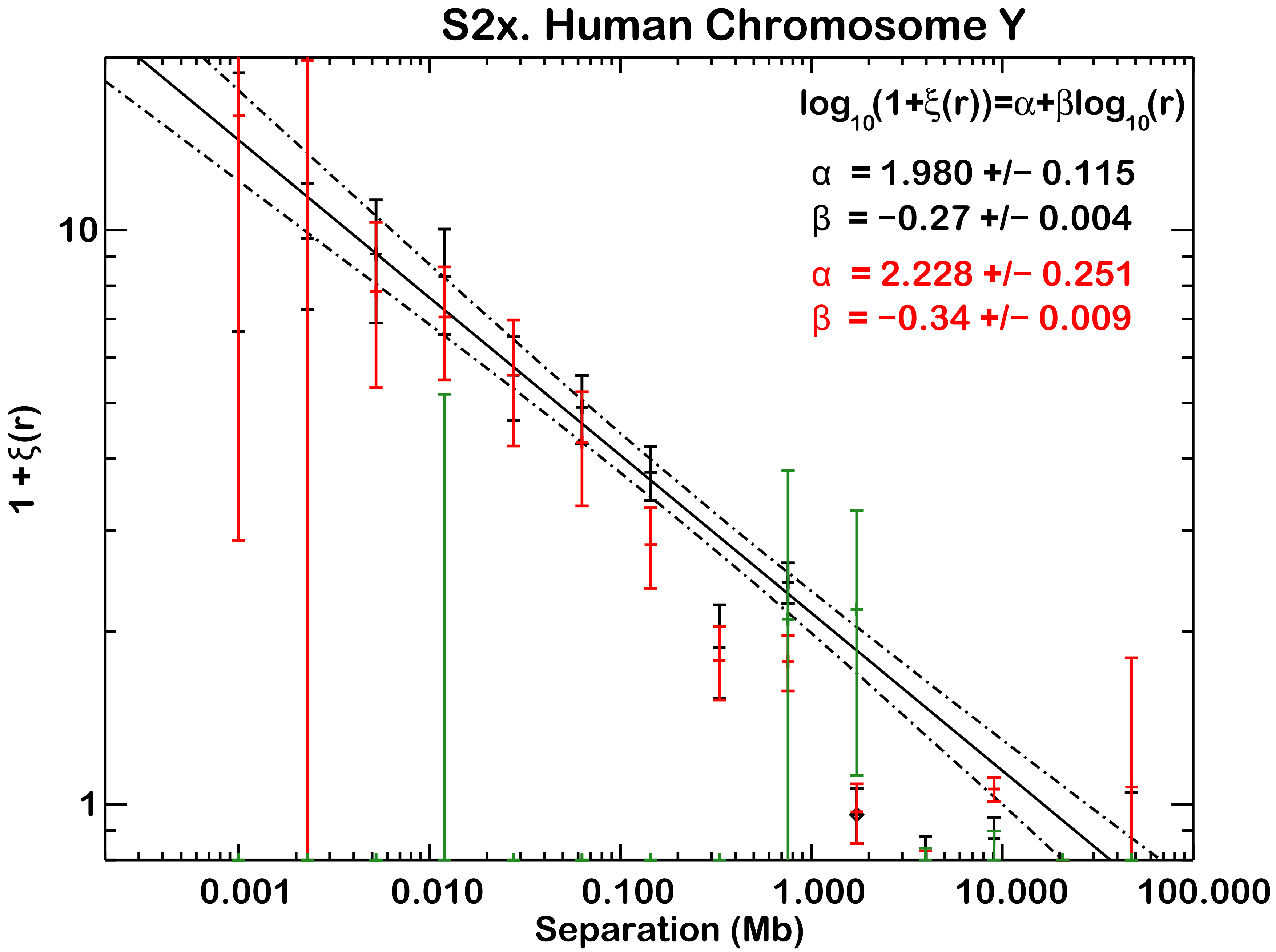

S2x. Human Chromosome Y
log10(1+ξ(r))=α+βlog10(r)
α = 1.980 +/− 0.115
β = −0.27 +/− 0.004
α = 2.228 +/− 0.251
β = −0.34 +/− 0.009
10
1
0.001
0.010
0.100
1.000
10.000
100.000
Separation (Mb)
1 + ξ(r)
